# Radical Formation by Direct Single Electron Transfer between Nitrobenzene and Anionic Organo Bases

**DOI:** 10.1021/acsomega.5c02989

**Published:** 2025-06-02

**Authors:** Shivaprasad Achary Balahoju, Nicholus Bhattacharjee, Luis Lezama, Xabier Lopez, Pablo Salcedo-Abraira, Antonio Rodríguez-Diéguez, Daniel Reta

**Affiliations:** † 226245 Donostia International Physics Centre (DIPC), Paseo Manuel Lardizabal 4, Donostia 20018, Euskadi, Spain; ‡ Department of Polymers and Advanced Materials, Faculty of Chemistry, 16402 The University of the Basque Country, UPV/EHU, Paseo Manuel Lardizabal 3, Donostia 20018, Euskadi, Spain; § Inorganic Chemistry, University of the Basque Country, Barrio Sarriena, s/n, 48940 Leioa, Bizkaia, Spain; ∥ Department of Inorganic Chemistry, 16741 University of Granada. Av. Fuente Nueva s/n, 18071 Granada, Spain; ⊥ IKERBASQUE, Basque Foundation for Science, Bilbao 48011, Euskadi, Spain

## Abstract

The presence of unpaired electrons, i.e., radicals, equips organic molecules with unique magnetic and reactivity properties. However, due to the reactive nature of radicals and the nontrivial chemistry required for their preparation, strict structural and electronic limitations are imposed on the available systems, limiting their potential applications. Thus, developing mechanisms that enable facile radical formation in simple reaction conditions, employing available and inexpensive reactants and applicable to general types of molecules, holds the key to capitalize on the extraordinary properties that radicals have to offer. Here, combining electron paramagnetic resonance spectroscopy and ab initio calculations, we uncover an unprecedented single electron transfer from multiple anionic organic bases (B^–^X^+^) to nitrobenzene [**1**], leading to the formation of stable nitrobenzenide radical ion-pair [**1** ^**•–**^]­[**X** ^**+**^] (X = Li, Na, K) and transient oxidized, radical bases B^•^. Our results establish nitroarenes as versatile radical precursors, providing a broadly applicable protocol for generating heteroatom-centered radicals and enabling radical transformations under mild conditions from inexpensive and readily available starting materials. Finally, we propose the [**1**]–[B^–^X^+^] couple as an unexplored platform with the potential to advance the field of frustrated radical pairs.

## Graphical abstract

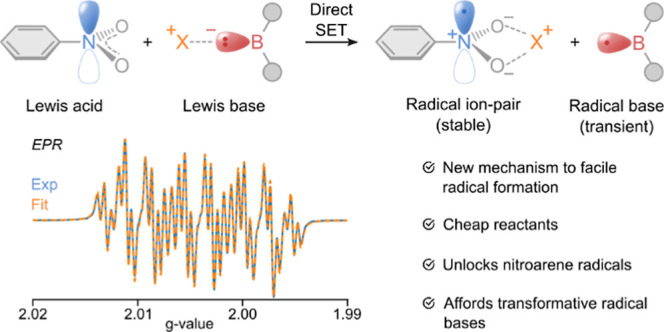

## 1 Introduction

Nitrogen-centered radicals (NCRs) offer a very versatile toolkit in organic chemistry. Thanks to their varying degree of stability^1^ and multiple generation schemes,^2^ they have found a wide range of applications as intermediates in novel synthetic pathways,^3–5^ platforms for obtaining persistent high-spin (poly)­radicals,^6–8^ frustrated Lewis pairs,^9,10^ and frustrated radical pairs.^11,12^ Moreover, their relevance goes well beyond chemistry, extending to medicine with NCRs being key drug components,^13^ and chemical biology, where reactive nitrogen species participate in a number of metabolic processes.^14^

Among NCRs, nitroarene radicals, with the unpaired electron centered in a nitro group that delocalizes in the π-system of an arene, are species of particular importance in multiple chemical transformations. For instance, they are involved as transient species in hydrogen atom transfer reactions;^15^ trans-annulation processes;^16^ photoinduced,^17^ electrochemical,^18^ and redox^19^ organic transformations; polymerizations;^20^ and metabolic processes with potential biological applications.^21^ As an example of their versatility, nitroarenes also support the formation of diradical transient species generated with visible light that can be used as anaerobic oxidants for obtaining carbonyl and imine^22^ and alkene^23^ derivatives and to promote oxygen atom transfer,^24^ C–H hydroxylation of aliphatic systems,^25^ C–C bond cleavage of olefins,^26^ and hydroxylation of olefins through C–N bond cleavage.^27^

The simplest nitroarene is nitrobenzene (**[1]**), where the arene group is a bare phenyl ring. Nitrobenzene radical anion ([**1** ^**•–**^] or nitrobenzenide) has received extensive attention in the past decades. The first stable [**1** ^**•–**^] was reported by Weissman and co-workers via sodium metal reduction and characterized using electron paramagnetic resonance (EPR), where they report “a spectrum of at least ten peaks, covering about 25 oersteds”, but no assignment was performed.^28^ This work showed that the nitro group could act as a Lewis acid, opening the possibility of hosting unpaired electrons and enacting radical chemistry in a commonly found organic molecule. Ever since, efforts have focused on understanding the electronic structure of [**1** ^**•–**^] in different conditions, mainly by means of EPR. An early example of the sensitivity of [**1** ^**•–**^] EPR spectra is Rieger’s computational study, who demonstrated that the solvent, in this case dimethylformamide and acetonitrile, plays a significant role in the hyperfine coupling constants (HFCs) of [**1** ^**•–**^].^29^ Some years later, Geske and Maki reported the in situ electrochemical formation and EPR characterization of [**1** ^**•–**^] via cathodic reduction in acetonitrile with *n*-Pr_4_N^+^ClO_4_^–^ as buffer in a mercury pool electrode.^30^ They were able to model the EPR spectra considering a monoradical (*g* = 2.0032) experiencing HFCs to one nitrogen and two ortho, two meta, and one para hydrogen atom (|*A*_N_| = 10.32, |*A*_Ho_| = 3.39, |*A*_Hm_| = 1.09, |*A*_Hp_| = 3.97 G, respectively—see eq 1 and Table S1). Ward expanded this work by showing that [**1** ^**•–**^] can also be formed using Na and K metals as reducing agents in 1,2-dimethoxyethane (DME)^31^ and that the spectra obtained with potassium was comparable to Geske’ electrochemical data. Continuing with the use of metals as reducing agents, Ling and Gendell first reported the formation of contact ion pairs [**1** ^**•–**^]­[**X** ^**+**^] (X = Li, Na, K, Cs) in DME,^32^ where the HFCs to the nuclear spins of the alkali metals (I = 3/2 for ^7^Li, ^23^Na, and ^39^K, I = 7/2 for ^133^Cs) were resolved. They found sizeable variations of all HFCs with temperature and a combination of separated and contact ion pairs in mixed solutions of DME and dimethyl sulfoxide (DMSO). Smentowski then reported [**1** ^**•–**^] formation using Lithium, Sodium, and Potassium in liquid ammonia solution at −50 °C,^33^ whose HFCs were comparable to those measured in other solvents electrochemically.^30^ Gross et al. performed a detailed study of the effect of the solvent polarity on the contact (separated) ion pair formation and the HFCs of [**1** ^**•–**^]­[**X** ^**+**^] (X = Li, Na, K, Rb, Cs).^34,35^ They conclude that the measured EPR line widths are directly proportional to the ionic radius of the alkali counterion and again found large variations depending on the media used. This is consistent with what was found by Stevenson and Echegoyen, who used the very polar hexamethyl phosphoramide solvent and found that it favors separated ion pair formation.^36^ Structural characterization was advanced by Mason et al., who reported the principal values of the *g*- and *A*_N_ tensors in co-crystallization studies.^37^ Along these lines, Kochi et al. resolved the single-crystal structures of [**1** ^**•–**^]­[**X** ^**+**^] (X = K, Rb, Cs)^38^ using metal reduction of **[1]** and 18-crown-6 and [2.2.2] cryptand as chelating ligands—HFCs to all nuclear spin active atoms were assigned, clearly correlating structure to either separated or contact ion pair formation and its impact on EPR spectra. For clarity and from now on, the possibility of having separated or contact ion pairs is denoted as [**1** ^**•–**^]­([**X** ^**+**^]), indicating the absence ([**1** ^**•–**^]···[**X** ^**+**^], distance ∼ 6 Å) or presence ([**1** ^**•–**^]­[**X** ^**+**^], distance ∼ 3 Å) of a HFC to the counterion X^+^. Finally, a much less studied way of obtaining [**1** ^**•–**^] is by single electron transfer processes using anions,^39^ where reduction of **[1]** proceeds from deprotonated nitro-compounds and/or carbanions—these early studies^40–44^ have only been recently exploited to unlock new reactivity patterns on nitrostilbenes,^19^ highlighting the untapped potential that achieving [**1** ^**•–**^]­([**X** ^**+**^]) formation in milder conditions has to offer.

Here, we report on a novel and facile way to form [**1** ^**•–**^]­([**X** ^**+**^]) (X = Li, Na, K) by employing a wide variety of simple anionic organic bases in different solvents at room temperature (Scheme 1). Exhaustive EPR characterization coupled to detailed density functional theory (DFT) calculations support the reduction of **[1]** via single electron transfer (SET) from the anionic organic base—while SET is calculated to be slightly endothermic for most cases, further transformations of the resulting oxidized bases render the overall process thermodynamically favorable. To the best of our knowledge, this is the first time that nitrobenzenide [**1** ^**•–**^]­([**X** ^**+**^]) is shown to occur thermally via direct SET from anionic organic bases, echoing frustrated radical pairs.^11,12^ Our results present the added value of unlocking the extensive family of nitroarenes as platforms to implement radical-based chemistry, conveniently and directly from commercially available reagents, showing that these commonplace molecules still have a lot to offer.^45^

**Scheme 1:**
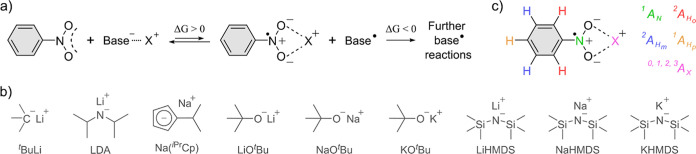
(a) Schematic Representation of [**1** ^**•–**^]­(**[X** ^**+**^ **]**) (X = Li, Na, K) Ion Pair Formation, Performed at Room Temperature and Inert Conditions with (b) Different Anionic Organo-Alkali Bases. (c) Number of Equivalent HFCs for Each Spin Active Nuclei Employed to Fit the EPR Data Using eq 1—Zero HFC to the X^+^ Counterion (*A*_X_) Signifies a separated Ion Pair, and • Denotes an Unpaired Electron

## 2 Results and Discussion

Given the abundant literature precedent showing [**1** ^**•–**^]­([**X** ^**+**^]) formation using alkali metals (X = Li, Na, K, Rb, Cs) and the fact that the nitro group can act as a Lewis acid,^46^ we wondered whether we could induce nitrobenzene reduction using other types of reactants. The choice of anionic organic bases (B^–^X^+^) seemed appropriate, given that they provide the alkali-metal counterion (X^+^) to form the needed ion-pair while also offering a readily available lone pair as a source of electrons (B^–^). In particular, we used *tert*-butyllithium (^*t*^BuLi), lithium diisopropylamide (LDA), lithium *tert*-butoxide (LiO^*t*^Bu), sodium *tert*-butoxide (NaO^*t*^Bu), potassium *tert*-butoxide (KO^*t*^Bu), lithium bis­(trimethylsilyl)­amide (LiHMDS), sodium bis­(trimethylsilyl)­amide (NaHMDS), potassium bis­(trimethylsilyl)­amide (KHMDS), and *sodium isopropyl cyclopentadienide* (Na^*i*Pr^Cp) bases in pure polar (tetrahydrofuran, THF) and apolar (benzene, C_6_H_6_) solvents as well as their combination with DMSO in varying mixing ratios (Scheme 1).

### 2.1 **[1** ^**•–**^ **]­([X** ^**+**^ **])** Electronic and Structural Characterization

Admittedly, to our surprise, this approach worked, and we could get clear continuous wave (CW) EPR signals using a wide range of anionic organic bases in different solvents at room temperature in fluid solution (Figure 1). For a bare [**1** ^**•–**^], one would expect a g-value slightly larger than 2.0023 because the unpaired electron is promoted to a previously unoccupied orbital in the nitro group.^47^ The electron spin then interacts with the nuclear spin of ^14^ *N* (I = 1), resulting in a 1:1:1 triplet, which is further split by the nuclear spins (I = 1/2) of one para and two sets of two equivalent meta and ortho hydrogen atoms, leading to a theoretical maximum number of 54 transitions. Then, the presence of alkali metals [**1** ^**•‑**^]­[**X** ^**+**^] (X = Li, Na, K) with nuclear spins I = 3/2 catapults these to a possible 216 peaks. Depending on the relative strength of the HFCs *A* (eq 1) and spectral resolution, these might overlap, simplifying the spectrum. Our results exemplify this, with spectra spanning from a single unresolved isotropic transition to extremely fine-structured signals with +190 peaks (Figures S23–S31).

**Figure 1:**
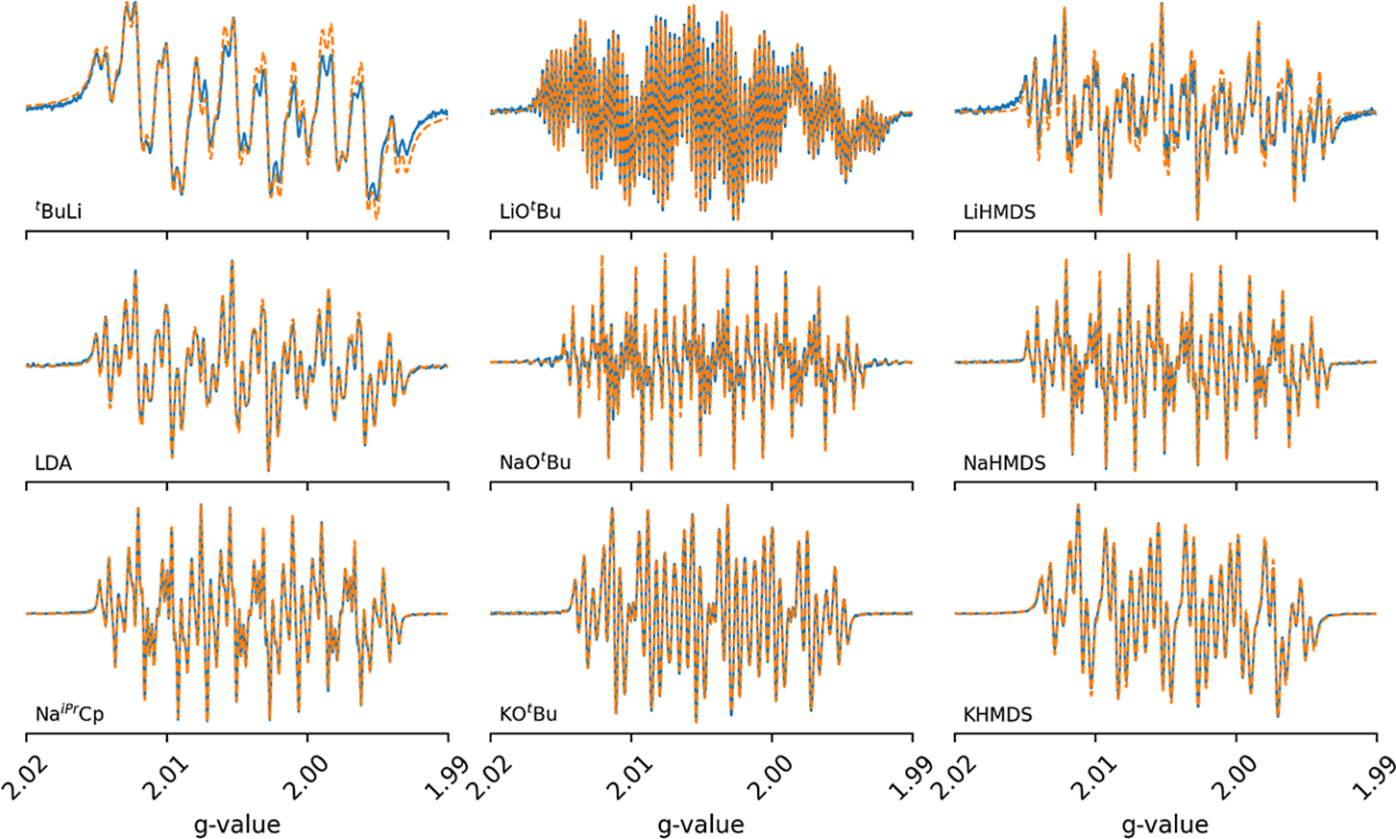
Comparison of measured (solid line) and fitted (dashed line) CW X-band EPR spectra of **[1** ^**•–**^ **]**(**[X** ^**+**^ **]**) obtained with different anionic organic bases at 298 K. The parameters of each fit are given in Table 1. More details are available in Table S1.

Despite this variation, regardless of the employed conditions, we always observe an EPR signal that can be assigned to an unpaired electron (*g* = 2.004) interacting with at least one nuclear spin of multiplicity I = 1 with a HFC of *ca* 11 G, pointing to the consistent presence of a nitro radical. The broad signals correlate with the appearance of a suspension in solution, and in an effort to avoid aggregation and gain spectral resolution, we tried one, or a combination, of the following: (i) decreasing the concentration of nitrobenzene, (ii) changing the molar ratio between nitrobenzene and anionic organic base (**[1]**:[B^–^X^+^]), and (iii) increasing the polarity of the medium by mixing THF and benzene with DMSO. This resulted in much more resolved signals, as exemplified nicely by LDA (Figure S24), whose spectrum with 25% of DMSO could be perfectly fitted to **[1** ^**•–**^ **]**, i.e., using one nuclear spin of I = 1, assigned to the nitrogen of the nitro, plus one and two sets of two equivalent nuclear spins of I = 1/2, assigned to the para, meta, and ortho hydrogen atoms, respectively (Figure 1, Table 1)—a similar behavior is observed with ^*t*^BuLi and LiHMDS, using even smaller amounts of DMSO (Figures S23 and S26).

**Table 1:** Comparison of Model Spin Hamiltonian Parameters Obtained by Fitting the EPR Spectra of **[1** ^**•–**^ **]**(**[X** ^**+**^]) under Different Conditions^a^

base	solvent	[**1**]	[**1**]:[B^–^X^+^]	chelate	*g*	| ***A***_**N**_|	| ***A***_**Hp**_|	| ***A***_**Ho**_|	| ***A***_**Hm**_|	| ***A***_**X**_|
^*t*^BuLi	C_6_H_6_/DMSO (85:15)	40	1:1	n/a	2.0038	11.55	3.83	3.41	1.12	n/a
LDA	THF/DMSO (75:25)	40	1:1	n/a	2.0040	11.41	3.87	3.42	1.12	n/a
Na^*i*Pr^Cp	THF	40	1:0.25	L_2_	2.0041	10.83	3.95	3.41	1.10	0.19
LiO^*t*^Bu	C_6_H_6_	40	1:5	n/a	2.0041	12.82	3.77	3.44	1.14	0.52
NaO^*t*^Bu	THF	40	1:0.5	L_2_	2.0041	10.83	3.98	3.40	1.09	0.19
KO^*t*^Bu	THF	10	1:1	L_1_	2.0042	9.44	4.07	3.35	1.05	n/a
LiHMDS	C_6_H_6_/DMSO (95:5)	40	1:0.5	n/a	2.0040	11.46	3.89	3.43	1.12	n/a
NaHMDS	THF	40	1:0.5	L_2_	2.0041	10.83	3.96	3.41	1.10	0.19
KHMDS	THF	10	1:1	L_1_	2.0041	9.43	4.07	3.34	1.04	n/a
Calculated										
**[1** ^**•–**^ **][Li** ^**+**^ **]**					2.004	9.012	3.975	3.696	1.491	1.124
**[1** ^**•–**^ **][Na** ^**+**^ **]**					2.005	8.325	3.880	3.537	1.392	1.547
**[1** ^**•–**^ **][K** ^**+**^ **]**					2.005	7.881	3.912	3.521	1.388	0.362

a When two solvents are given, values in parentheses indicate mixture %. Nitrobenzene concentration ([**1**]) and hyperfine coupling constants (*A*) are given in millimolar and Gauss, respectively. “Molar ratio” refers to the relative molar proportion of **[1]** and base. *A*_X_ refers to the alkali metal—for LiO^*t*^Bu, three equivalent I = 3/2 are needed. For calculated values, see Methods section.

For the remaining anionic organo bases, gaining spectral resolution revealed additional peaks in the spectra. In the case of KO^*t*^Bu and KHMDS, these could be accounted for by including a hyperfine interaction to a single I = 3/2 nuclear spin (Figures S16 and S20, Table S1. ^39^K 93% abundance). In an attempt to force the formation of separated ion pairs and reveal the signature of bare **[1** ^**•–**^ **]**, we employed chelating agents. When adding the [2.2.2] cryptand (L_1_), both KO^*t*^Bu and KHMDS spectra simplified even further and no hyperfine to K^+^ was needed (Figure 1 and Table 1). This was confirmed by single-crystal X-ray diffraction (XRD) data, which revealed a distance between [K­(L_1_)]^+^ and **[1** ^**•–**^ **]** of ∼6 Å (Figure 2, Section S2, and Table S2), in agreement with Kochi’s data^38^ (CSD entry EWOCUO)—note that Kochi’s structure was obtained by metal reduction, yet we obtain virtually the same crystalline structure. However, employing 15-crown-5 ether (L_2_) with KHMDS does not result in a separated ion pair (Figure S21) since chelation is expected to occur in the plane perpendicular to nitrobenzene. The situation is more complicated for LiO^*t*^Bu and all the sodium-based Lewis bases, as either one (Figures S9, S12–S14), two (Figures S10 and S11), or three (Figures S3–S5 and S8) I = 3/2 nuclear spins are needed to reproduce the spectra. Still, some clarity is gained when using 15-crown as the number of Na^+^ ions is decreased from three to one for NaO^*t*^Bu (Figures S8 vs S9) and from two to one for NaHMDS (Figures S11 vs S12). The fact that at least one ion is always needed to describe these spectra is ascribed to the employed L_1_ and L_2_ not being efficient at disrupting **[1** ^**•–**^ **]**···**[Na** ^**+**^ **]** interactions.^48^ Finally, we note that for NaO^*t*^Bu, there remain some satellite peaks that cannot be fully reproduced with a single Na^+^ ion (Figure S9), which likely originated from small fractions of higher nuclearity compounds still present in solution. For LiO^*t*^Bu, using 12-Crown-4 (L_3_), which is the most appropriately sized crown for Li^+^, still yields spectra that need three ions, suggesting that the formation of [**1** ^**•–**^]­[**Li** ^**+**^] is favored over that of [Li­(L_3_)]^+^. Unfortunately, and in contrast to **[1** ^**•–**^ **]**:[K­(L_1_)]^+^, all efforts to obtain single crystals of **[1** ^**•–**^ **]**:[X­(L_1, 2, 3_)]^+^ (X = Li, Na) failed.

**Figure 2:**
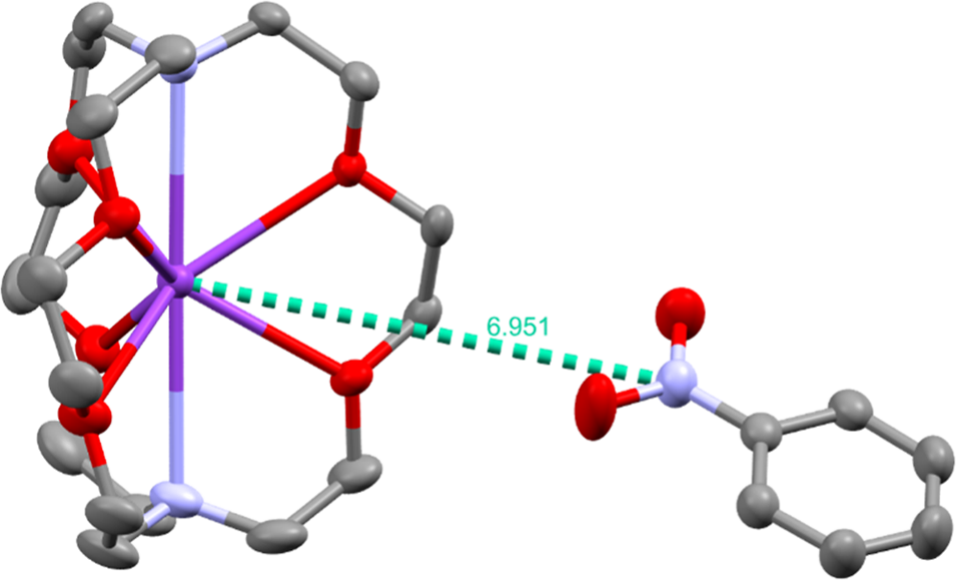
Structure of the separated ion pair in **[1** ^**•–**^ **]**:[K­(L_1_)]^+^ with typical interionic K^+^···NO_2_^–^ separation of ∼7 Å. Obtained by the mixture of **[1]**, KHMDS, and L_1_. The structure is virtually the same as the CSD entry EWOCUO, obtained by metal reduction.^38^ Potassium, carbon, oxygen, and nitrogen are represented in purple, gray, red, and blue, respectively. H atoms were omitted for clarity.

In summary, depending on the experimental conditions, all the EPR spectra obtained can be reproduced using a model spin Hamiltonian with parameters related to either **[1** ^**•–**^ **]** or [**1** ^**•–**^]­[**X**_***n***_^**+**^] (X = Li, Na, K, *n* = 1, 2, 3). The largest variation across all measurements is the HFC to the nitro nitrogen (Table 1), with its magnitude |*A*_N_| being inversely proportional to the ionic radius of the alkali metal, in good qualitative agreement with our calculations (vide infra). The trend in |*A*_N_| is also inversely correlated to the HFC to the alkali ion, as previously observed,^34,35^ reflecting that when the electron spin delocalized over more centers, their overall interaction weakens. We also note a large variation in the |*A*_N_| and |*A*_Hp_| parameters between the contact and separated ion pair for the potassium-containing bases. While |*A*_N_| decreases from 10.6 to 9.4 G, |*A*_Hp_| increases from 3.9 to 4.1 G for both KO^*t*^Bu and KHMDS (Figures S17, S18, and S20–S22). This is ascribed to the electron spin participating more effectively in all resonant forms of the aromatic ring when a distant ionic charge does not localize the spin density over the nitrogen atom. Finally, no other radicals have been measured in any of the experiments, suggesting that the lifetime of the oxidized species B^•^ are too short to be detected.

### 2.2 [**1** ^**•–**^]­(**[X** ^**+**^ **]**) Formation Mechanism

Having established that **[1** ^**•–**^ **]** is consistently obtained regardless of the employed conditions, we set out to understand its mechanism of formation by means of DFT calculations (see the Methods section). Given the homogeneity of the EPR data, we argue that the underlying mechanism must be common to all anionic organic bases.

#### 2.2.1 Direct SET from All Bases

A first reasonable guess would then be the reduction of nitrobenzene via single electron transfer (SET) from the anionic organic base. Such SET can happen following either an outer- or inner-sphere mechanism, that is, with the formation of the **[1X]** ^**+**^ cation adduct first and then the reduction from a dissociated salt (nonbonded) or the concomitant involvement of all species (bonded). The former process is predicted to be highly endothermic and is therefore discarded (Schemes S1–S5, Table S2). The latter is predicted to be exothermic for the ^*t*^BuLi and LDA, whereas it is slightly endothermic for the remaining anionic organic bases, which is further decreased if a fully dissociated base is considered (Figure 3a, Schemes S6–S11, Table S2). To assess these computational results, we performed comparative spin quantification experiments for **[1]** mixed with a solution of KHMDS and L_1_ in THF (Figure 3b). We chose this combination as it provided clear spectra where possible artifacts from the nuclear spin were not a concern (Figure 1). By keeping an equimolar ratio between **[1]** and KHMDS, while increasing that of L_1_ from 1:0.1 to 1:1, we observe a clear increase in the number of radicals (Figure 3c), confirming that a fully dissociated alkali salt undergoes a more favorable SET. Still, this is predicted to be endothermic with a transition state very close to the products ((1.1) and (1.2) in Figure 3a)—the transition state is approximated by scanning the broken symmetry electronic state with explicit THF molecules coordinating the cation (Scheme S6 bottom), otherwise, the reaction coordinate is not well-defined. This calculated potential energy surface (PES) would render the process highly skewed toward the reactants and therefore EPR silent, in contrast to what has been measured. Thus, we explored other options that would make the process thermodynamically viable.

**Figure 3:**
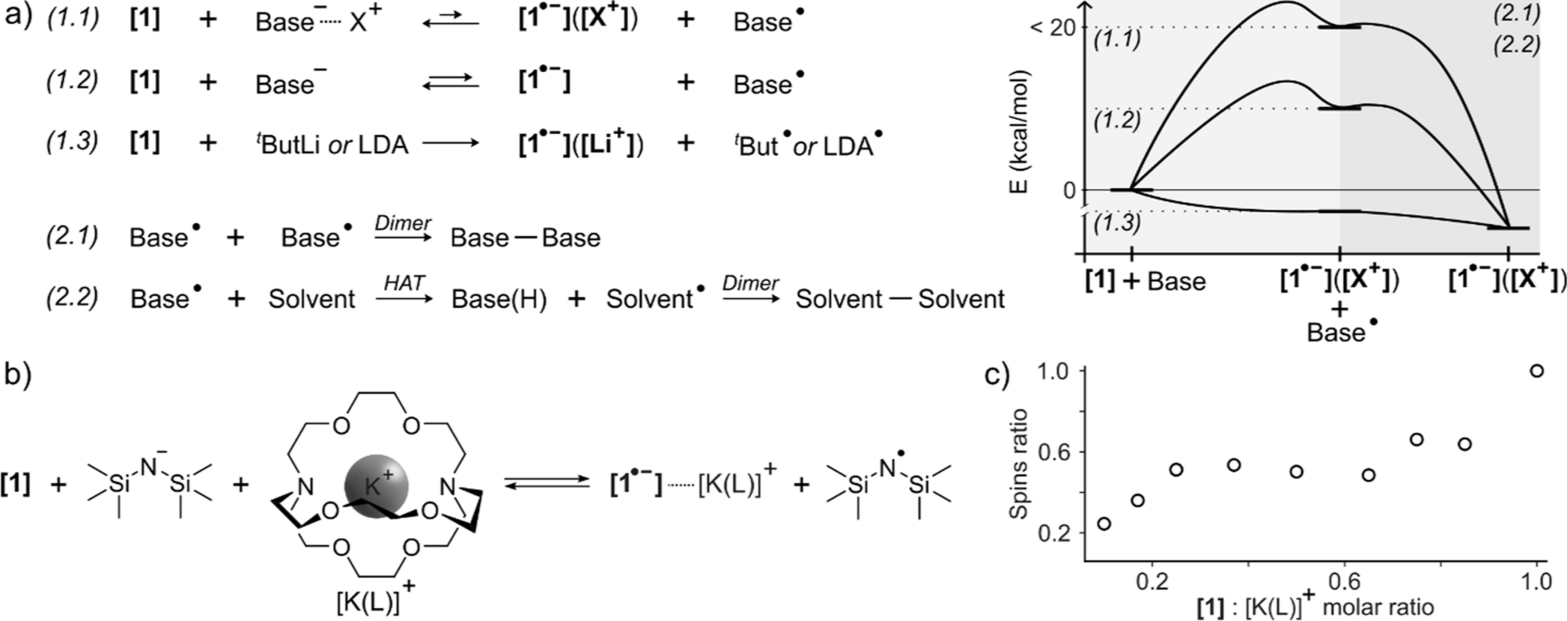
SET formation of **[1** ^**•–**^ **]**(**[X** ^**+**^ **]**). (a) Reaction mechanisms explored (left) and their schematic energy profile (right), differentiating SET and subsequent transformation of the oxidized base. (b) Scheme showing radical formation in the presence of fully dissociated KHMDS base by means of L_1_. (c) Comparative spin quantification experiments as a function of **[1]**:[K­(L_1_)]^+^ molar ratio, obtained by the reaction of 100 μL of **[1]** 10 mM, 12.5 μL of 80 mM KHMDS, and the corresponding μL of L_1_ 80 mM in THF.

#### 2.2.2 Transformations of the Oxidized Bases

First, we find that the dimerization of the oxidized bases is calculated to always be exothermic ((2.1) in Figure 3a, Schemes S12–S18, and Table S2), resulting in an overall reaction energy Δ*G* of −22, +4, 0, +1, −28, −26, and −24, kcal/mol for Na^*i*Pr^Cp, LiO^*t*^Bu, NaO^*t*^Bu, KO^*t*^Bu, LiHMDS, NaHMDS, and KHMDS, respectively. Radical dimerization of O^*t*^Bu^•^ and HMDS^•^ would yield di-*tert*-butyl peroxide^49^ and tetrakis­(trimethylsilyl)­hydrazine,^50,51^ which are relatively stable known compounds. However, comparative quantitative ^1^H NMR DOSY experiments in *d*-THF of NaO^*t*^Bu and KHMDS, before and after reaction with **[1]**, do not reveal large enough differences in the diffusion coefficients to account for dimerization (Figures S47 and S49). Additionally, these differences could be ascribed to the formation of aggregates of NaO^*t*^Bu^52,53^ and KHMDS^54^ in THF, which would be disrupted by the formation of [**1** ^**•–**^]­(**[X** ^**+**^ **]**). Thus, while this remains a predicted possible pathway, especially for the XHMDS bases, we were unable to experimentally validate it.

Because the dimerization of the alkoxide bases renders the overall process slightly endothermic, we also investigate hydrogen atom transfer (HAT) reactions from the solvent molecules ((2.2) in Figure 3a and Schemes S25–S33), as these are typical mechanisms invoked for O^*t*^Bu^•^ ^55^ and HMDS^•^ ^12^ radicals. The disappearance of the resulting radical-containing solvent molecule from the medium is assumed to occur via highly exothermic dimerization (Schemes S49–S51). We find that for both radical bases, THF preferentially favors HAT over benzene and that subsequent solvent dimerization favors the process (Table S2). However, as for the base dimerization, we were unable to detect the dimerized solvent molecules in either ^1^H DOSY NMR (Figures S47 and S49) or ^2^H NMR (Figure S50). Still, we believe that the presented computational results validate this mechanism.

Finally, O^*t*^Bu^•^ radicals have been reported to decompose and form acetone and methyl radicals,^56^ but we could not detect either species in our ^1^H NMR data (Figure S46). Mass spectroscopy was also not informative to assess the formation of these products.

#### 2.2.3 Other Mechanisms for XO^*t*^Bu Bases

There are several works in the literature reporting on the reducing capacity of *tert*-butoxides.^12,57–62^ Of particular relevance to our study, also dealing with XO^*t*^Bu and nitroarenes, are the early works of Janzen^40–43^ and Guthrie,^44^ together with the more recent study by Driver.^19^

Janzen et al. studied the reaction of *o*-and *p*-nitrotoluene with KO^*t*^Bu in *t*-butyl alcohol and DMSO and observed *p*,*p*′-dinitrobibenzyl dimer formation.^40–43^ *p*-Nitrotoluene radical anion was characterized by means of EPR and proposed to be the key intermediate in the formation of the dimers, but its mechanism of formation remained unexplained. Further studies by Buncel using UV–vis spectroscopy^63^ confirmed that the sequence consists of nitrotoluene deprotonation by the base, followed by a SET from nitrotoluene anion to nitrotoluene neutral. However, our DFT calculations predict all the steps to be endothermic for **[1]** (Schemes S52–S57, Table S2). We also looked at a potential reduction of **[1]** by a deprotonated solvent molecule, finding that while this SET is exothermic, the prior deprotonation is not favored (>25 kcal/mol, Schemes S34–S45, Table S2). Thus, we can confidently conclude that this mechanism does not operate the formation of [**1** ^**•–**^]­(**[X** ^**+**^ **]**) in the present case.

Guthrie and Nutter studied the reaction of **[1]** also with KO^*t*^Bu in THF and observed the formation of *tert*-butoxynitrobenzene.^44^ Following Janzen’s original reasoning, they propose that the obtained product proceeds via the formation of **[1** ^**•–**^ **]**. The process is initiated by a nucleophilic attack of the base to **[1]**, followed by the deprotonation of this anion intermediate by another base, and finalized by the reduction of two **[1]** molecules by the dianion, as summarized by reactions 1, 5, and 6 in their work. Note that their reasoning was based on indirect evidence, as no EPR characterization of the proposed species was provided. Our DFT calculations with NaO^*t*^Bu predict a barrier of 10 kcal/mol for the nucleophilic attack, followed by a highly impeded subsequent deprotonation by a second base molecule and a highly exothermic final reduction product (Scheme S58 top)—a similar profile, with smaller barriers, is maintained when the Na^+^ ion is removed (Scheme S58 bottom), but this would imply a full dissociation of the salt, in contrast to its known tendency to form clusters in THF.^52,53^ Additionally, this mechanism would result in the formation of *tert*-butoxynitrobenzene, but we did not observe the corresponding chemical shifts in NMR experiments (Figure S46). Finally, to assess whether this mechanism could be active for other bases, we studied the first nucleophilic attack with the ^*i*Pr^Cp^–^ anion, finding a barrier of >19 kcal/mol (Scheme S59), which is not competitive against the proposed direct SET at 3 kcal/mol. Thus, this mechanism is also discarded as a valid general option in our case.

More recenlty, Driver et al. studied the reaction of nitrostilbenes with XO^*t*^Bu in THF and observed the formation of *N*-hydroxyindoles or oxindoles when X = Na or K, respectively.^19^ EPR studies were employed to argue that **[1** ^**•–**^ **]**, in the case of NaO^*t*^Bu, and a combination of oxygen- and carbon-centered radicals, in the case of KO^*t*^Bu, were the key intermediates enabling product formation. However, we have shown that both alkoxides lead to the same species (Table 1). Despite Driver’s impressive results, this discrepancy might arise from their broad EPR line widths, which hamper the resolution of all HFCs, as well as a lack of mechanistic insight guided by computational studies. Another stark difference is the reported absence of reaction progress when using LiO^*t*^Bu, in contrast to our results that clearly demonstrate **[1** ^**•–**^ **]­[Li** ^**+**^ **]** formation under these conditions. To the best of our knowledge, this is the most closely related work to our results, but here we provide an unequivocal characterization and clear formation mechanism of **[1** ^**•–**^ **]**(**[X** ^**+**^ **]**) under a broader set of conditions.

## 3 Conclusion

By means of an exhaustive spectroscopic and computational characterization, we have revealed a direct SET from multiple anionic organic bases to nitrobenzene to afford the facile formation of **[1** ^**•–**^ **]**(**[X** ^**+**^ **]**) ion pairs—this is fundamentally different from previous reports where SET is mediated by an intermediate anion.^40–44^

For ^*t*^BuLi and LDA bases, direct SET is an exothermic process that occurs readily. For Na^*i*Pr^Cp, XO^*t*^Bu, and XHMDS (X = Li, Na, K), it is slightly endothermic, with further transformations of the oxidized base, such as dimerization and/or HAT of solvent molecules, rendering the whole process thermally accessible. This interpretation is further supported by the absence of other radical species in all our EPR spectra and the fact that reaction of **[1]** with the Bronsted base TBD does not yield any radical (Scheme S11, Table S2). For XO^*t*^Bu and XHMDS (X = Li, Na), and depending on the reaction conditions, we observe nonstoichiometric ratios between **[1** ^**•–**^ **]** and the number of equivalent I = 3/2 nuclear spins needed to fit the EPR data, which is tentatively assigned to aggregate formation.^64–66^ **[1** ^**•–**^ **]**(**[X** ^**+**^ **]**) is formed regardless of the conditions employed, but adding a chelating agent to force a separated ion pair has proven to be the most efficient strategy to gain spectral resolution and obtain reliable model spin Hamiltonian parameters. We have also shown that salt dissociation favors SET, offering a handle toward controlling the amount of generated radical species by an appropriate choice of the medium. Other reaction mechanisms for **[1** ^**•–**^ **]**(**[X** ^**+**^ **]**) formation were explored using DFT calculations but deemed thermodynamically less competitive than the direct SET.

Our work demonstrates that the nitrobenzenide radical—and, by extension, oxidized bases—can be accessed under a significantly broader and more straightforward range of conditions than previously reported. This provides a synthetically convenient protocol to obtain nitrobenzenide radical ion pairs, in a gram scale, which can then be stored under an inert atmosphere and used as starting material for subsequent reactions. Our results also present a significant resemblance to recent findings in the field of frustrated radical pairs,^11^ where SET enables novel synthetic pathways using some of the oxidized bases we report here (O^*t*^Bu^•^ and HMDS^•^).^12^ Thus, we propose the **[1]**–[B^–^X^+^] tandem as a versatile and untapped Lewis pair, with the potential to equip nitroarenes with radical properties and easily produce oxidized, radical bases; current efforts in our group focus on exploiting the radical reactivity offered by these molecules to access valuable organic transformations.

## 4 Methods

### 4.1 Materials

Nitrobenzene **[1]**, the anionic organic bases ^*t*^BuLi, LDA, Na^*i*Pr^Cp, LiO^*t*^Bu, NaO^*t*^Bu, KO^*t*^Bu, LiHMDS, NaHMDS, and KHMDS, as well as the nonnucleophilic base 1,5,7-triazabicyclo[4.4.0]­dec-5-ene (TBD) and the chelating ligands 4,7,13,16,21,24-hexaoxa-1,10-diazabicyclo[8.8.8] hexacosane (L_1_), 15-crown-5 (L_2_), and 12-crown-4 (L_3_) were purchased from Sigma-Aldrich and used directly. The employed solvents (tetrahydrofuran, benzene, and *n*-hexane) were high-performance liquid chromatography grade anhydrous solvents, purchased from Sigma-Aldrich and used without further purification. All sample manipulation and preparation were performed under a dry and inert atmosphere inside a glovebox.

### 4.2 Preparation of Nitrobenzene Radical Anions [**1** ^**•–**^]­(**[X** ^**+**^ **]**)

Two stock solutions of nitrobenzene 40 mM in THF and benzene were prepared in separate vials. 80 mM stock solutions of the different bases employed were also prepared in THF and benzene, except for ^*t*^BuLi 1.7 M in pentane, LDA 1.0 M in THF, LiO^*t*^Bu 1.0 M in THF, and LiHMDS 1.0 M in THF. 100 μL from the nitrobenzene stock solutions (4 × 10^–6^ mol) were added to each base solution containing either a 1:1 or 1:5 molar ratio. Immediately upon addition, we observe the solution turning from pale yellow to deep red or purples colors.

### 4.3 Spectroscopy

#### 4.3.1 EPR

Continuous-wave (CW) X-band (9.38 GHz) EPR spectra of liquid solutions of **[1** ^**•–**^ **]**(**[X** ^**+**^ **]**) were collected on a Bruker EMX Plus EPR spectrometer with a 0.6 T electromagnet. The samples were prepared inside the glovebox and transferred into a quartz EPR tube (4 mm outer diameter). For each sample, we performed a two-dimensional measurement of signal intensity against microwave power to ensure we are in the nonsaturating regime. Fitting and simulation of the recorded EPR spectra were performed using the Xepr Bruker software, assuming a model spin Hamiltonian that consists of a sum of electron-Zeeman and electron-nuclei hyperfine interactions

1 $$Ĥ={Ĥ}_{EZ}+{Ĥ}_{HF}=\mu_{B}{{\mathrm{B⃗}}_{0}}^{T}g_{k}{\vec{\hat{S}}}_{k}+\sum_{i}{{\vec{\hat{S}}}_{k}}^{T}A_{ki}{\vec{\hat{I}}}_{i}$$

where index k refers to electron spin, index i runs over all nuclear spins, and the symbol *T* denotes the transpose of a vector ( ${{\mathrm{B⃗}}_{0}}^{T}$ ) or vector operator ( ${{\vec{\hat{S}}}_{k}}^{T}$ ). Note that all the measurements have been performed in liquid solution, so the ***g***_**k**_ and ***A***_**ki**_ tensors can be simplified to diagonal matrices. All fittings were performed allowing for second order corrections and anisotropic line width variations.

#### 4.3.2 Optical Electronic Spectra

UV–vis spectroscopic measurements were performed with the JASCO V-730 spectrophotometer in the range of 200–500 nm, using quartz cuvettes (1 cm pathway length). All spectra were baseline corrected using the spectrum of the pure solvent.

6.0 mg of nitrobenzene was dissolved in 5.0 mL THF (9.75 mM). 102 μL of the stock solution was diluted into 2.0 mL THF to make a 0.5 mM solution, from which 200 μL were further dissolved in 1.8 mL of THF to achieve a final concentration of 5 × 10^–5^ M. This solution was measured to determine the absorption spectrum of neutral nitrobenzene, showing a 261 nm band with a molar absorption coefficient of ε = 8.9 × 10^3^ M^–1^ cm^–1^. Addition of NaO^*t*^Bu base equimolar solution (19.2 μL of 5.20 mM) inside the glovebox turned the solution into a pale brown color. The UV–vis absorption of this sample presents an extra band at 354 nm with a molar absorption coefficient ε = 1.0 × 10^3^ M^–1^ cm^–1^, along with the main band at 261 nm ε = 9.2 × 10^3^ M^–1^ cm^–1^ (Figure S32).

### 4.4 DFT Calculations

The reaction mechanisms of radical formation by various bases were investigated through DFT^31^ calculations, using the Gaussian16 software package.^67^ If not stated otherwise, all calculations were performed in the gas phase. The hybrid B3LYP functional^68–70^ and the augmented correlation-consistent polarized valence double-ζ (aug-cc-pVDZ) basis set were employed for H, C, N, and O,^71,72^ and Li and Na,^73^ and K,^74^ using Basis Set Exchange^75,76^ —the (un)­restricted formalism was adopted for open-shell and closed-shell species, respectively. Grimme’s D3 damping function was used to account for dispersion corrections.^77^ Initial molecular structures of all reactants and products were generated with GaussView software, and each reactant and product were optimized individually. Subsequent calculations of the Hessian were performed to confirm the presence of true stationary minima on the PES, with non-negative frequencies and converged forces. All of the reported reaction energies (Δ*G*) are in kcal/mol and have been calculated using the sum of electronic and thermal free energies. To assess the validity of our choice of functional to describe the SET process, we also employed a range of functionals, finding a small deviation in the Δ*G* values. In particular, we used CAM-B3LYP,^78^ LC-*w*PBE,^79^ and WB97XD,^80^ the latter two with and without dispersion (Table S5).

Model spin Hamiltonian parameters were calculated using Orca5.0.4 ^81^ at the optimized geometries of **[1** ^**•–**^ **]­[X** ^**+**^ **]** (X = Li, Na, K) in the doublet electronic state. The hybrid B3LYP functional and modified augmented version of the Def2-TZVP basis set (ma-Def2-TZVP)^82^ were employed. g-Tensor values for all electronic spins as well as isotropic Fermi contact (AISO), anisotropic dipolar contribution (ADIP), and the orbital hyperfine (AORB) terms for all nitrogen, sodium, and hydrogen nuclei were calculated. A polarizable continuum model (PCM) for solvation was included using THF as the solvent.

To compare with the experimental UV–vis spectra, comparative time-dependent density functional theory calculations on **[1]** and **[1** ^**•–**^ **]­[Na** ^**+**^ **]** were performed at the same level of theory used for geometry optimization. For better comparison to experiment, a PCM model for solvation was employed with THF as the solvent. Both for **[1]** and **[1** ^**•–**^ **]­[Na** ^**+**^ **]** systems, 100 roots were requested.

## Supplementary Material



## Data Availability

The data supporting this article have been included as part of the Supporting Information. Additional raw EPR and NMR spectra can be found at 10.5281/zenodo.14900603. All the inputs and outputs of the presented calculations can be found at 10.17172/NOMAD/2025.03.08-1, within the upload ID https://nomad-lab.eu/prod/v1/gui/upload/id/ue41kCiESJCubexAzsoAGw.

## Notes

EPR spectra, single-crystal XRD data, DFT calculations and reaction pathways, UV–vis absorption spectra, and NMR spectra (PDF)

## References

[1] Hioe J., Šakić D., Vrček V., Zipse H. (2015). The Stability of Nitrogen-Centered
Radicals. Org. Biomol. Chem..

[2] Yu X. Y., Zhao Q. Q., Chen J., Xiao W. J., Chen J. R. (2020). When Light
Meets Nitrogen-Centered Radicals: From Reagents to Catalysts. Acc. Chem. Res..

[3] Xiong T., Zhang Q. (2016). New Amination Strategies Based on
Nitrogen-Centered Radical Chemistry. Chem. Soc.
Rev..

[4] Bjørsvik H. R., Liguori L., Minisci F. (2001). New Selective Oxidation Reactions
by Nitroarenes in Basic Medium Involving Electron-Transfer Processes. Org. Process Res. Dev..

[5] Pratley C., Fenner S., Murphy J. A. (2022). Nitrogen-Centered Radicals in Functionalization
of Sp2 Systems: Generation, Reactivity, and Applications in Synthesis. Chem. Rev..

[6] Rajca A., Olankitwanit A., Wang Y., Boratyński P. J., Pink M., Rajca S. (2013). High-Spin s = 2 Ground State Aminyl
Tetraradicals. J. Am. Chem. Soc..

[7] Boratyński P., Pink M., Rajca S., Rajca A., Boratyn P. J., Rajca S., Rajca A., Pink M. (2010). Isolation of the Triplet
Ground State Aminyl Diradical. Angew. Chem.,
Int. Ed..

[8] Rajca A., Shiraishi K., Pink M., Rajca S. (2007). Triplet (S = 1) Ground
State Aminyl Diradical. J. Am. Chem. Soc..

[9] van
der Zee L. J. C., Hofman J., van Gaalen J. M., Slootweg J. C. (2024). Mechanistic Studies on Single-Electron Transfer in
Frustrated Lewis Pairs and Its Application to Main-Group Chemistry. Chem. Soc. Rev..

[10] van
der Zee L. J. C., Pahar S., Richards E., Melen R. L., Slootweg J. C. (2023). Insights into Single-Electron-Transfer Processes in
Frustrated Lewis Pair Chemistry and Related Donor-Acceptor Systems
in Main Group Chemistry. Chem. Rev..

[11] Ju M., Lu Z., Novaes L. F. T., Martinez Alvarado J.
I., Lin S. (2023). Frustrated
Radical Pairs in Organic Synthesis. J. Am. Chem.
Soc..

[12] Lu Z., Ju M., Wang Y., Meinhardt J. M., Martinez Alvarado J. I., Villemure E., Terrett J. A., Lin S. (2023). Regioselective Aliphatic
C–H Functionalization Using Frustrated Radical Pairs. Nature.

[13] Nepali K., Lee H. Y., Liou J. P. (2019). Nitro-Group-Containing
Drugs. J. Med. Chem..

[14] Phaniendra A., Jestadi D. B., Periyasamy L. (2015). Free Radicals:
Properties, Sources,
Targets, and Their Implication in Various Diseases. Indian J. Clin. Biochem..

[15] Lu C., Su Z., Jing D., Jin S., Xie L., Li L., Zheng K. (2019). Intramolecular Reductive Cyclization of O-Nitroarenes
via Biradical
Recombination. Org. Lett..

[16] Shoberu A., Li C. K., Qian H. F., Zou J. P. (2021). Copper-Catalyzed,
N-Auxiliary Group-Controlled Switchable Transannulation/Nitration
Initiated by Nitro Radicals: Selective Synthesis of Pyridoquinazolones
and 3-Nitroindoles. Org. Chem. Front..

[17] Wise D. E., Gogarnoiu E. S., Duke A. D., Paolillo J. M., Vacala T. L., Hussain W. A., Parasram M. (2022). Photoinduced Oxygen Transfer Using
Nitroarenes for the Anaerobic Cleavage of Alkenes. J. Am. Chem. Soc..

[18] Wagenknecht J. H. (1977). Reaction
of Electrogenerated Nitrobenzene Radical Anion with Alkyl Halides. J. Org. Chem..

[19] Zhao Y., Zhu H., Sung S., Wink D. J., Zadrozny J. M., Driver T. G. (2021). Counterion
Control of T-BuO-Mediated Single Electron Transfer to Nitrostilbenes
to Construct N-Hydroxyindoles or Oxindoles. Angew. Chem., Int. Ed..

[20] Encinas M. V., Rufs A. M., Norambuena E., Giannotti C. (2000). Polymerization
of Vinyl Monomers Photoinitiated by P-Nitroaniline: Photoinitiation
Mechanism. J. Polym. Sci., Part A:Polym. Chem..

[21] Penning T. M., Su A. L., El-Bayoumy K. (2022). Nitroreduction:
A Critical Metabolic
Pathway for Drugs, Environmental Pollutants, and Explosives. Chem. Res. Toxicol..

[22] Mitchell J. K., Hussain W. A., Bansode A. H., O’Connor R. M., Wise D. E., Choe M. H., Parasram M. (2023). Photoinduced
Nitroarenes
as Versatile Anaerobic Oxidants for Accessing Carbonyl and Imine Derivatives. Org. Lett..

[23] Paolillo J. M., Saleh M. R., Junk E. W., Parasram M. (2025). Merging Photoexcited
Nitroarenes with Lewis Acid Catalysis for the Anti-Markovnikov Oxidation
of Alkenes. Org. Lett..

[24] Parasram M., Parasram M., Wise D. E., Parasram M. (2023). Photoexcited Nitroarenes
as Anaerobic Oxygen Atom Transfer Reagents. Synlett.

[25] Paolillo J. M., Duke A. D., Gogarnoiu E. S., Wise D. E., Parasram M. (2023). Anaerobic
Hydroxylation of C­(Sp3)-H Bonds Enabled by the Synergistic Nature
of Photoexcited Nitroarenes. J. Am. Chem. Soc..

[26] Wise D. E., Gogarnoiu E. S., Duke A. D., Paolillo J. M., Vacala T. L., Hussain W. A., Parasram M. (2022). Photoinduced Oxygen Transfer Using
Nitroarenes for the Anaerobic Cleavage of Alkenes. J. Am. Chem. Soc..

[27] Hampton C., Simonetti M., Leonori D. (2023). Olefin Dihydroxylation Using Nitroarenes
as Photoresponsive Oxidants. Angew. Chem., Int.
Ed..

[28] Chu T. L., Pake G. E., Paul D. E., Townsend J., Weissman S. I. (1953). Paramagnetic
Resonance Absorption of Free Radicals. J. Phys.
Chem..

[29] Rieger P. H., Fraenkel G. K. (1963). Analysis of the
Electron Spin Resonance Spectra of
Aromatic Nitrosubstituted Anion Radicals. J.
Chem. Phys..

[30] Geske D. H., Maki A. H. (1960). Electrochemical
Generation of Free Radicals and Their
Study by Electron Spin Resonance Spectroscopy; the Nitrobenzene Anion
Radical. J. Am. Chem. Soc..

[31] Ward R. L. (1961). An Electron
Spin Resonance Study of Nitro Group-Alkali Metal Interactions in Aromatic
Hydrocarbons. J. Am. Chem. Soc..

[32] Ling C. Y., Gendell J. (1967). ESR Studies of Nitrobenzene,
O-Dinitrobenzene, and
M-Dinitrobenzene Alkali-Metal Salts. J. Chem.
Phys..

[33] Smentowski F. J., Stevenson C. D. (1968). Anion Radicals
in Liquid Ammonia. J. Am. Chem. Soc..

[34] Gross J. M., Barnes J. D., Pillans G. N. (1969). Radical
Intermediates. Part I. Spectroscopic
Studies on the Alkali-Metal Nitrobenzenides. J. Chem. Soc. A.

[35] Gross J. M., Barnes J. D. (1970). Radical Intermediates. IV. Electron Spin Resonance
Studies on the Alkali Metal Nitrobenzenides in Nitrilic Solvents. J. Phys. Chem..

[36] Stevenson C. D., Echegoyen L., Lizardi L. R. (1972). Equilibrium studies by electron spin
resonance. I. Free nitrobenzene anion radical. J. Phys. Chem..

[37] Mason R. P., Harriman J. E. (1976). ESR Investigation of the Nitrobenzene Anion Radical
in Single Crystals of Benzoate Salts. J. Chem.
Phys..

[38] Davlieva M.
G., Lü J. M., Lindeman S. V., Kochi J. K. (2004). Crystallographic
Distinction between “Contact” and “Separated”
Ion Pairs: Structural Effects on Electronic/ESR Spectra of Alkali-Metal
Nitrobenzenides. J. Am. Chem. Soc..

[39] Buck P. (1969). Reactions
of Aromatic Nitro Compounds with Bases. Angew
Chem. Int. Ed. Engl..

[40] Russell G. A., Janzen E. G., Strom E. T. (1962). The Formation of Radical-Anions by
Electron Transfer Between Anions and Their Unsaturated Analogs in
Dimethyl Sulfoxide Solution. J. Am. Chem. Soc..

[41] Russell G. A., Janzen E. G. (1962). Spontaneous Formation
of Radical-Anions from Nitroaromatics
in Basic Solution. J. Am. Chem. Soc..

[42] Russell G. A., Janzen E. G., Strom E. T. (1964). Electron-Transfer
Processes. I. The
Scope of the Reaction between Carbanions or Nitranions and Unsaturated
Electron Acceptors. J. Am. Chem. Soc..

[43] Russell G. A., Janzen E. G. (1967). Electron Transfer
Processes. VI. Disproportionation
of o- and p-Nitrotoluenes in Basic Solution. J. Am. Chem. Soc..

[44] Guthrie R. D., Nutter D. E. (1982). Mechanism of the Apparent Electron-Transfer Reaction
between Tert-Butoxide Ion and Nitrobenzene. J. Am. Chem. Soc..

[45] Gkizis P. L., Triandafillidi I., Kokotos C. G. (2023). Nitroarenes The Rediscovery of Their
Photochemistry Opens New Avenues in Organic Synthesis. Chem..

[46] Pogoreltsev A., Tulchinsky Y., Fridman N., Gandelman M. (2017). Nitrogen Lewis
Acids. J. Am. Chem. Soc..

[47] Jeschke, G. Electron Paramagnetic Resonance. Eq. 1. 1. 5 in https://chem.libretexts.org/Bookshelves/Physical_and_Theoretical_Chemistry_Textbook_Maps/Electron_Paramagnetic_Resonance_(Jenschke)/01%3A_Introduction/1.01%3A_General_Remarks (accessed Feb 20, 2025).

[48] Fabbrizzi L. (2020). The Origins
of the Coordination Chemistry of Alkali Metal Ions. ChemTexts.

[49] RajanBabu, T. V. ; Simpkins, N. S. ; RajanBabu, T. V. 1,1-Di-Tert-Butyl Peroxide. In Encyclopedia of Reagents for Organic Synthesis; John Wiley & Sons, 2005; . 10.1002/047084289X.RD066.PUB2.

[50] Ru
Hwu J., Wang N. (1988). Counterattack Reagents:Hexamethyldisilane and 1,2-Dimethyl-1,1,2,2-Tetraphenyldisilane
in the Synthesis of Polysilylated Hydrazines. Tetrahedron.

[51] Brain P. T., Irving I. A., Rankin D. W. H., Robertson H. E., Leung W. P., Bühl M. (1997). Determination of the Molecular Structure
of Tetrakis­(Trimethylsilyl)­Hydrazine, N2­(SiMe3)­4, in the Gas Phase
by Electron Diffraction. J. Mol. Struct..

[52] Østreng E., Sønsteby H. H., Øien S., Nilsen O., Fjellvåg H. (2014). Atomic Layer
Deposition of Sodium and Potassium Oxides: Evaluation of Precursors
and Deposition of Thin Films. Dalton Trans..

[53] Nekola H., Olbrich F., Behrens U. (2002). Kristall-Und
Molekülstrukturen
von Lithium-Und Natrium-Tert-Butoxid Crystal and Molecular Structures
of Lithium and Sodium Tert-Butoxide. Z. anorg.
allg. Chem..

[54] Spivey J. A., Collum D. B. (2024). Potassium Hexamethyldisilazide
(KHMDS): Solvent-Dependent
Solution Structures. J. Am. Chem. Soc..

[55] Mayer J. M. (2011). Understanding
Hydrogen Atom Transfer: From Bond Strengths to Marcus Theory. Acc. Chem. Res..

[56] Williams A. L., Oberright E. A., Brooks J. W. (1956). The Abstraction of Hydrogen Atoms
from Liquid Hydrocarbons by T-Butoxy Radicals. J. Am. Chem. Soc..

[57] Barham J. P., Coulthard G., Emery K. J., Doni E., Cumine F., Nocera G., John M. P., Berlouis L. E. A., McGuire T., Tuttle T., Murphy J. A. (2016). KOtBu: A Privileged Reagent for Electron
Transfer Reactions?. J. Am. Chem. Soc..

[58] Nocera G., Young A., Palumbo F., Emery K. J., Coulthard G., McGuire T., Tuttle T., Murphy J. A. (2018). Electron Transfer
Reactions: KO TBu (but Not NaO TBu) Photoreduces Benzophenone under
Activation by Visible Light. J. Am. Chem. Soc..

[59] Emery K. J., Young A., Arokianathar J. N., Tuttle T., Murphy J. A. (2018). KOtBu as
a Single Electron Donor? Revisiting the Halogenation of Alkanes with
CBr4 and CCl4. Molecules.

[60] Mąkosza M. (2021). Does Nucleophilic
Substitution in Nitroarenes Proceed via Single Electron Transfer (SET)?. Eur. J. Org Chem..

[61] Zhou S., Anderson G. M., Mondal B., Doni E., Ironmonger V., Kranz M., Tuttle T., Murphy J. A. (2014). Organic Super-Electron-Donors:
Initiators in Transition Metal-Free Haloarene–Arene Coupling. Chem. Sci..

[62] Xu Y., Shi X., Wu L. (2019). TBuOK-Triggered Bond Formation Reactions. RSC Adv..

[63] Buncel E., Menon B. C. (1980). Proton-Transfer and Electron-Transfer Processes in
Reaction of p-Nitrotoluene with Bases. A Spectrophotometric Study. J. Am. Chem. Soc..

[64] Münch A., Knauer L., Ott H., Sindlinger C., Herbst-Irmer R., Strohmann C., Stalke D. (2020). Insight into the Bonding
and Aggregation of Alkyllithiums by Experimental Charge Density Studies
and Energy Decomposition Analyses. J. Am. Chem.
Soc..

[65] Davison N., Hemingway J. M., Waddell P. G., Lu E. (2024). Lithium, Sodium and
Potassium Enolate Aggregates and Monomers: Syntheses and Structures. Dalton Trans..

[66] Davison N., Lu E. .. (2023). The Quest for Organo-Alkali Metal Monomers: Unscrambling the Structure–Reactivity
Relationship. Dalton Trans..

[67] Frisch, M. J. ; Trucks, G. W. ; Schlegel, H. B. ; Scuseria, G. E. ; Robb, M. A. ; Cheeseman, J. R. ; Scalmani, G. ; Barone, V. ; Mennucci, B. ; Petersson, G. A. ; Nakatsuji, H. ; Caricato, M. ; Li, X. ; Hratchian, H. P. ; Izmaylov, A. F. ; Bloino, J. ; Zheng, G. ; Sonnenberg, J. L. ; Hada, M. ; Ehara, M. ; Toyota, K. ; Fukuda, R. ; Hasegawa, J. ; Ishida, M. ; Nakajima, T. ; Honda, Y. ; Kitao, O. ; Nakai, H. ; Vreven, T. ; Montgomery, J. A., Jr. ; Peralta, J. E. ; Ogliaro, F. ; Bearpark, M. ; Heyd, J. J. ; Brothers, E. ; Kudin, K. N. ; Staroverov, V. N. ; Kobayashi, R. ; Normand, J. ; Raghavachari, K. ; Rendell, A. ; Burant, J. C. ; Iyengar, S. S. ; Tomasi, J. ; Cossi, M. ; Rega, N. ; Millam, J. M. ; Klene, M. ; Knox, J. E. ; Cross, J. B. ; Bakken, V. ; Adamo, C. ; Jaramillo, J. ; Gomperts, R. ; Stratmann, R. E. ; Yazyev, O. ; Austin, A. J. ; Cammi, R. ; Pomelli, C. ; Ochterski, J. W. ; Martin, R. L. ; Morokuma, K. ; Zakrzewski, V. G. ; Voth, G. A. ; Salvador, P. ; Dannenberg, J. J. ; Dapprich, S. ; Daniels, A. D. ; Farkas, O. ¨. ; Foresman, J. B. ; Ortiz, J. V. ; Cioslowski, J. ; Fox, D. J. Gaussian 16; Gaussian Inc.: Wallingford CT, 2016.

[68] Becke A. D. (1988). Density-Functional
Exchange-Energy Approximation with Correct Asymptotic Behavior. Phys. Rev. A.

[69] Becke A. D. (1993). Density-functional
Thermochemistry. III. The Role of Exact Exchange. J. Chem. Phys..

[70] Lee C., Yang W., Parr R. G. (1988). Development of the Colle-Salvetti
Correlation-Energy Formula into a Functional of the Electron Density. Phys. Rev. B.

[71] Dunning T. H. (1989). Gaussian
Basis Sets for Use in Correlated Molecular Calculations. I. The Atoms
Boron through Neon and Hydrogen. J. Chem. Phys..

[72] Kendall R. A., Dunning T. H., Harrison R. J. (1992). Electron
Affinities of the First-row
Atoms Revisited. Systematic Basis Sets and Wave Functions. J. Chem. Phys..

[73] Prascher B. P., Woon D. E., Peterson K. A., Dunning T. H., Wilson A. K. (2011). Gaussian
Basis Sets for Use in Correlated Molecular Calculations. VII. Valence,
Core-Valence, and Scalar Relativistic Basis Sets for Li, Be, Na, and
Mg. Theor. Chem. Acc..

[74] Hill J. G., Peterson K. A. (2017). Gaussian Basis Sets
for Use in Correlated Molecular
Calculations. XI. Pseudopotential-Based and All-Electron Relativistic
Basis Sets for Alkali Metal (K-Fr) and Alkaline Earth (Ca-Ra) Elements. J. Chem. Phys..

[75] Schuchardt K. L., Didier B. T., Elsethagen T., Sun L., Gurumoorthi V., Chase J., Li J., Windus T. L. (2007). Basis Set Exchange:
A Community Database for Computational Sciences. J. Chem. Inf. Model..

[76] Pritchard B. P., Altarawy D., Didier B., Gibson T. D., Windus T. L. (2019). New Basis
Set Exchange: An Open, Up-to-Date Resource for the Molecular Sciences
Community. J. Chem. Inf. Model..

[77] Grimme S., Antony J., Ehrlich S., Krieg H. (2010). A Consistent and Accurate
Ab Initio Parametrization of Density Functional Dispersion Correction
(DFT-D) for the 94 Elements H-Pu. J. Chem. Phys..

[78] Yanai T., Tew D. P., Handy N. C. (2004). A New Hybrid Exchange–Correlation
Functional Using the Coulomb-Attenuating Method (CAM-B3LYP). Chem. Phys. Lett..

[79] Iikura H., Tsuneda T., Yanai T., Hirao K. (2001). A Long-Range
Correction
Scheme for Generalized-Gradient-Approximation Exchange Functionals. J. Chem. Phys..

[80] Chai J. D., Head-Gordon M. (2008). Long-Range Corrected Hybrid Density Functionals with
Damped Atom–Atom Dispersion Corrections. Phys. Chem. Chem. Phys..

[81] Neese F. (2022). Software Update:
The ORCA Program System—Version 5.0. Wiley Interdiscip. Rev.:Comput. Mol. Sci..

[82] Weigend F., Ahlrichs R. (2005). Balanced Basis Sets of Split Valence, Triple Zeta Valence
and Quadruple Zeta Valence Quality for H to Rn: Design and Assessment
of Accuracy. Phys. Chem. Chem. Phys..

