# Supplementary material for: Radical Formation by Direct Single Electron Transfer between Nitrobenzene and Anionic Organo Bases
Source: ACS Omega. 2025 Jun 2;10(22):23798–807. doi: 10.1021/acsomega.5c02989 (PMC12163852; doi:10.1021/acsomega.5c02989)
Supplement: Supplementary file 1 [file ao5c02989_si_001.pdf]

Radical Formation by Direct Single Electron Transfer between Nitrobenzene and Anionic Organo Bases

S. A. Balahoju,[a] N. Bhattacharjee,[a] L. Lezama,[c] X. Lopez,[b] P. Salcedo-Abraira,[d] A. Rodríguez-Diéguez,[d] D. Reta*[a,b,e]

[a] Donostia International Physics Centre (DIPC), Donostia, 20018, Euskadi, Spain.
[b] Faculty of Chemistry, The University of the Basque Country, UPV/EHU, Donostia, 20018, Euskadi, Spain
[c] Departamento de Química Orgánica e Inorgánica, Facultad de Ciencia y Tecnología, Universidad del País Vasco, Bº Sarriena s/n, 48940 Leioa, Spain
[d] Department of Inorganic Chemistry, University of Granada. Av. Fuente nueva s/n, 18071 Granada, Spain
[e] IKERBASQUE, Basque Foundation for Science, Bilbao, 48011, Euskadi, Spain

Table of Contents

Section S1: EPR spectra. 2
Section S2: Single crystal XRD data. 26
Section S3: DFT calculations & reaction pathways. 27
Section S4: Uv-Vis absorption spectra. 45
Section S5: NMR spectra. 50

Section S1: EPR spectra.

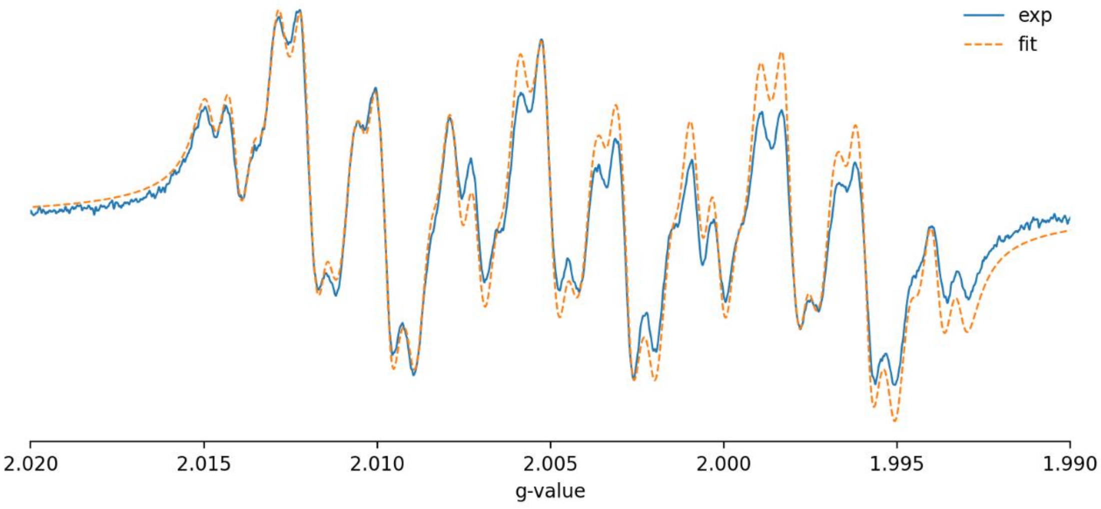

Figure S1. Comparison of normalised measured (solid line) and fitted (dashed line) cw-EPR spectra of [**1**$^{\bullet-}$], at room temperature. 100 μL (85 μL of benzene plus 15 μL of DMSO) of a 40 mM solution of nitrobenzene were mixed with 2.35 μL of tButLi 1.7M in *n*-pentane. Measured with 27 dB attenuation, 0.1 Gauss modulation amplitude and 7 scans. Fitting parameters are given in Table S1.

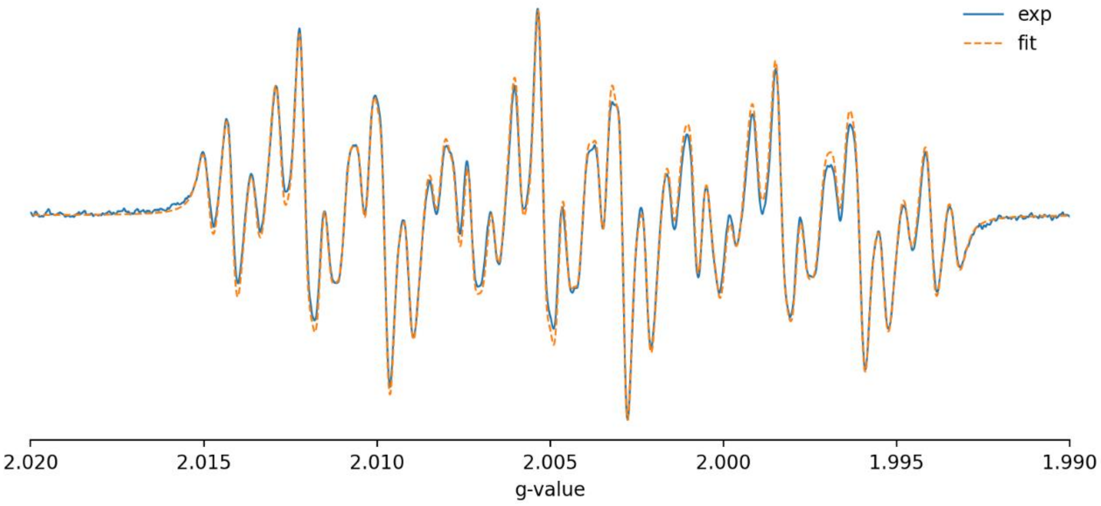

Figure S2. Comparison of normalised measured (solid line) and fitted (dashed line) cw-EPR spectra of [**1**$^{\bullet-}$], at room temperature. 100 μL (75 μL of THF plus 25 μL of DMSO) of a 40 mM solution of nitrobenzene were mixed with 4.0 μL of LDA 1.0 M in THF. Measured with 30 dB attenuation, 0.1 Gauss modulation amplitude and 35 scans. Fitting parameters are given in Table S1.

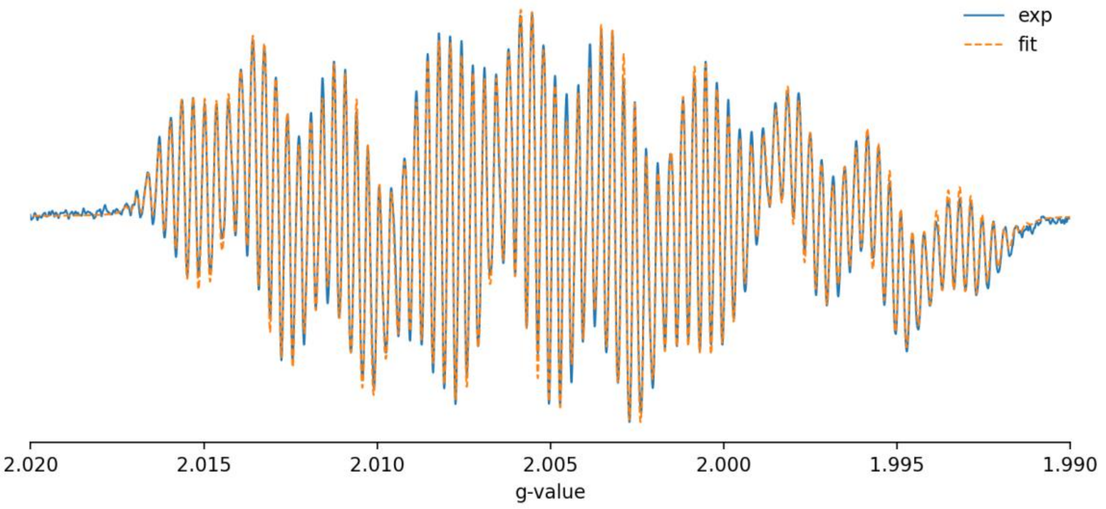

Figure S3. Comparison of normalised measured (solid line) and fitted (dashed line) cw-EPR spectra of [$\mathbf{1}^{\bullet-}$], at room temperature. 100 μL of a 40 mM solution of nitrobenzene in benzene were mixed with 20 μL of LiO^{t}Bu 1.0 M in THF (ratio 1:5). Measured with 20 dB attenuation, 0.1 Gauss modulation amplitude and 5 scans. Fitting parameters are given in Table S1. Note that the employed model required using three equivalent Li ions.

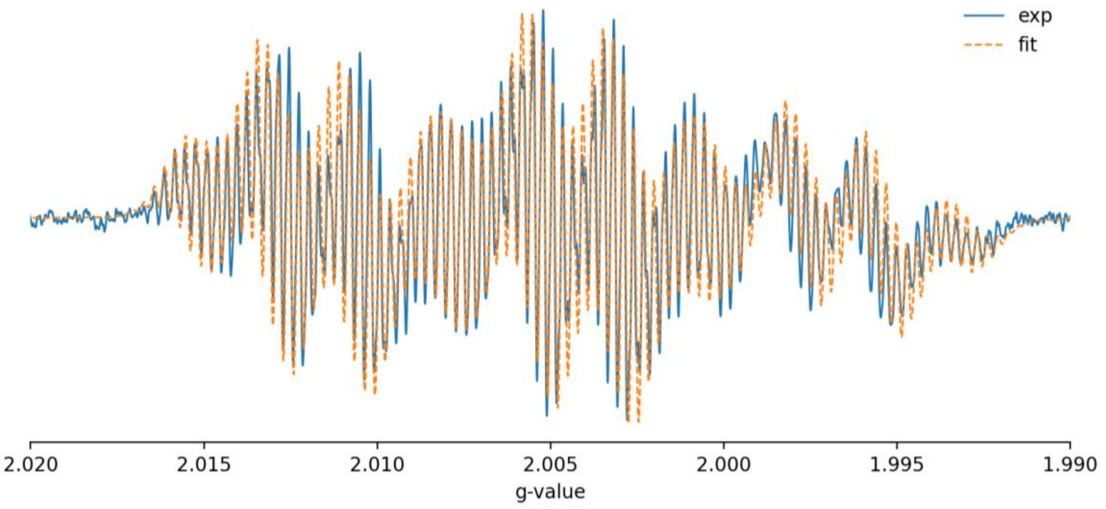

Figure S4. Comparison of normalised measured (solid line) and fitted (dashed line) cw-EPR spectra of [$\mathbf{1}^{\bullet-}$], at room temperature. 100 μL (95 μL of benzene plus 5 μL of DMSO) of a 40 mM solution of nitrobenzene were mixed with 4 μL of LiO^{t}Bu 1.0 M in THF (ratio 1:1). Measured with 20 dB attenuation, 0.1 Gauss modulation amplitude and 5 scans. Fitting parameters are given in Table S1. Note that the employed model required using three equivalent Li ions.

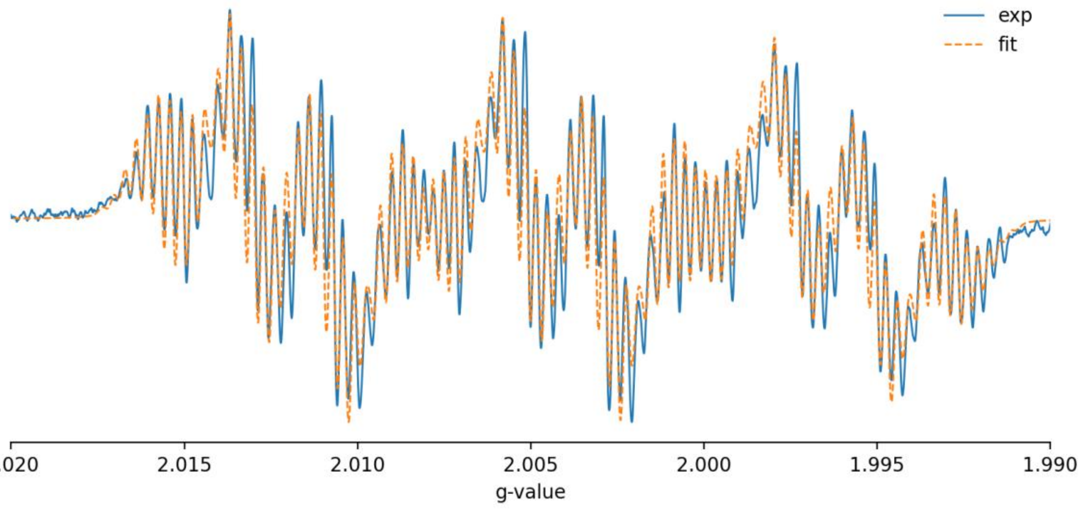

Figure S5. Comparison of normalised measured (solid line) and fitted (dashed line) cw-EPR spectra of [**1**$^{\bullet-}$], at room temperature. 100 μL of a 40 mM solution of nitrobenzene in benzene were mixed with 20 μL of LiHMDS 1.0 M in THF (ratio 1:5). Measured with 30 dB attenuation, 0.1 Gauss modulation amplitude and 25 scans. Fitting parameters are given in Table S1. Note that the employed model required using three equivalent Li ions.

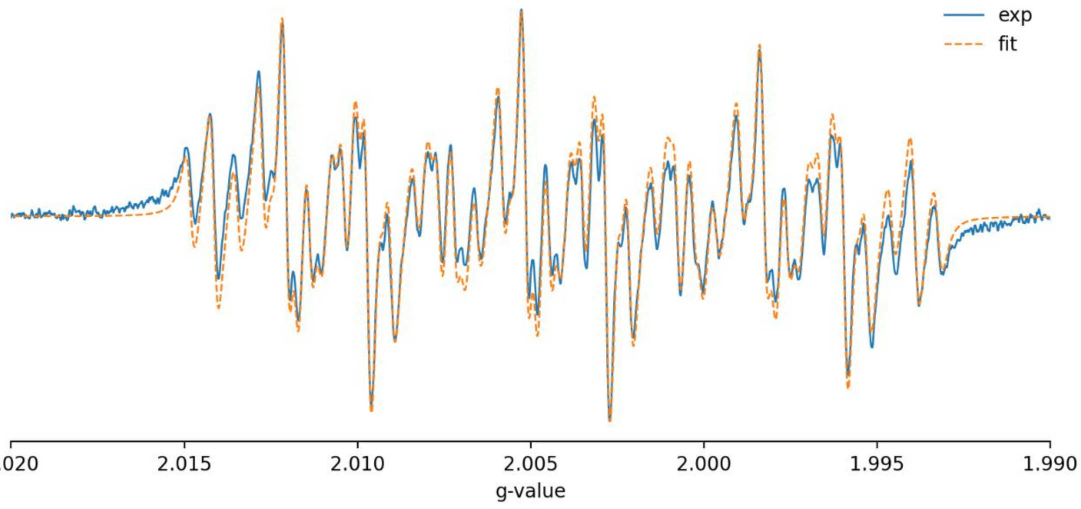

Figure S6. Comparison of normalised measured (solid line) and fitted (dashed line) cw-EPR spectra of [**1**$^{\bullet-}$], at room temperature. 100 μL (95 μL of THF plus 5 μL of DMSO) of a 40 mM solution of nitrobenzene were mixed with 2 μL of LiHMDS 1.0 M in THF (ratio 1:0.5). Measured with 25 dB attenuation, 0.1 Gauss modulation amplitude and 3 scans. Fitting parameters are given in Table S1.

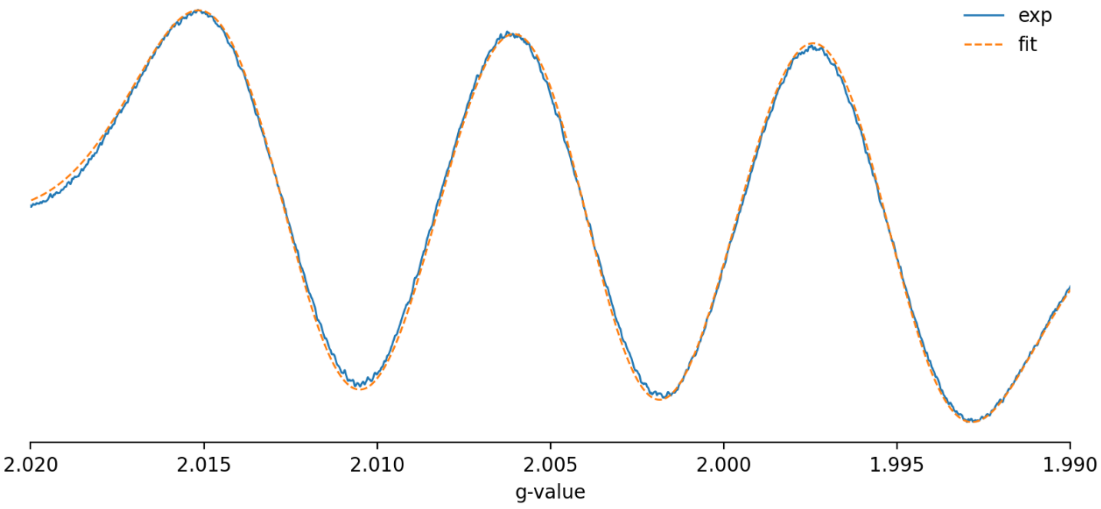

Figure S7. Comparison of normalised measured (solid line) and fitted (dashed line) cw-EPR spectra of [**1**$^{•-}$], at room temperature. 100 μL of a 40 mM solution of nitrobenzene in benzene were mixed with 50 μL of NaO^{t}Bu 80 mM in benzene. Measured with 20 dB attenuation, 0.1 Gauss modulation amplitude and 5 scans. Fitting parameters are given in Table S1.

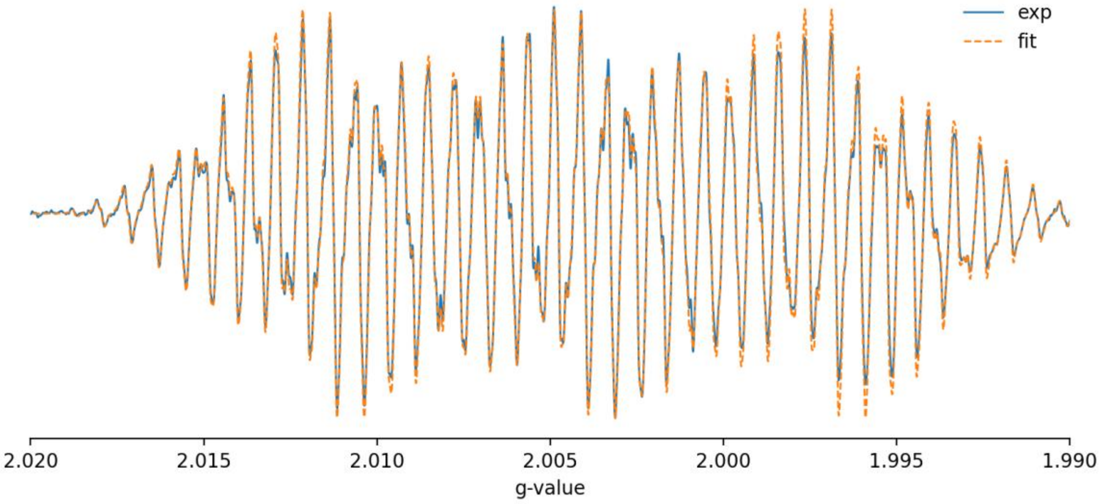

Figure S8. Comparison of normalised measured (solid line) and fitted (dashed line) cw-EPR spectra of [**1**$^{•-}$], at room temperature. 100 μL of a 40 mM solution of nitrobenzene in THF were mixed with 50 μL of NaO^{t}Bu 80 mM in THF. Measured with 15 dB attenuation, 0.1 Gauss modulation amplitude and 5 scans. Fitting parameters are given in Table S1. Note that the employed model required using three equivalent Na ions.

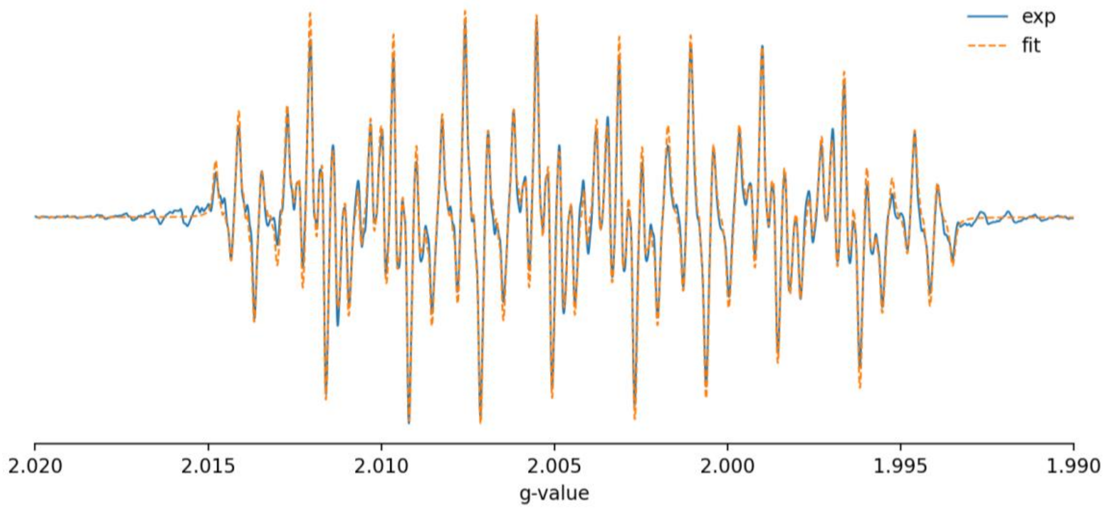

Figure S9. Comparison of normalised measured (solid line) and fitted (dashed line) cw-EPR spectra of [$\mathbf{1}^{\bullet-}$], at room temperature. 100 μL of a 40 mM solution of nitrobenzene in THF were mixed with 50 μL of NaO^{t}Bu 80 mM in THF and 50μL of 15-crown ether 80 mM in THF. Measured with 20 dB attenuation, 0.1 Gauss modulation amplitude and 8 scans. Fitting parameters are given in Table S1. Note that the employed model required using just one Na ion. Satellite peaks at both extremes indicate the presence of a minority component of higher nuclearity.

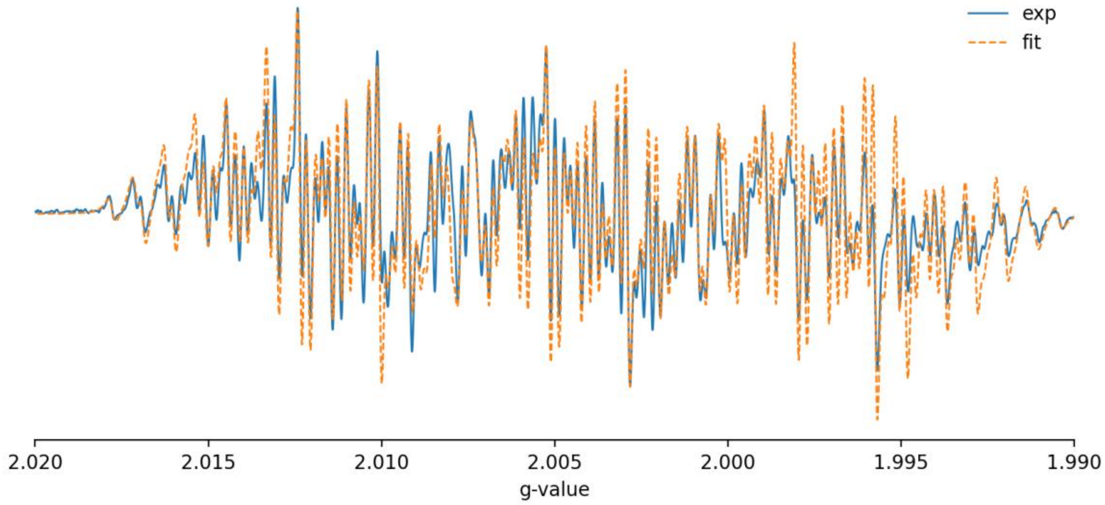

Figure S10. Comparison of normalised measured (solid line) and fitted (dashed line) cw-EPR spectra of [$\mathbf{1}^{\bullet-}$], at room temperature. 100 μL of a 40 mM solution of nitrobenzene in THF were mixed with 50 μL of NaHMDS 80 mM in THF. Measured with 20 dB attenuation, 0.1 Gauss modulation amplitude and 5 scans. Fitting parameters are given in Table S1. Note that the employed model required using two equivalent Na ions.

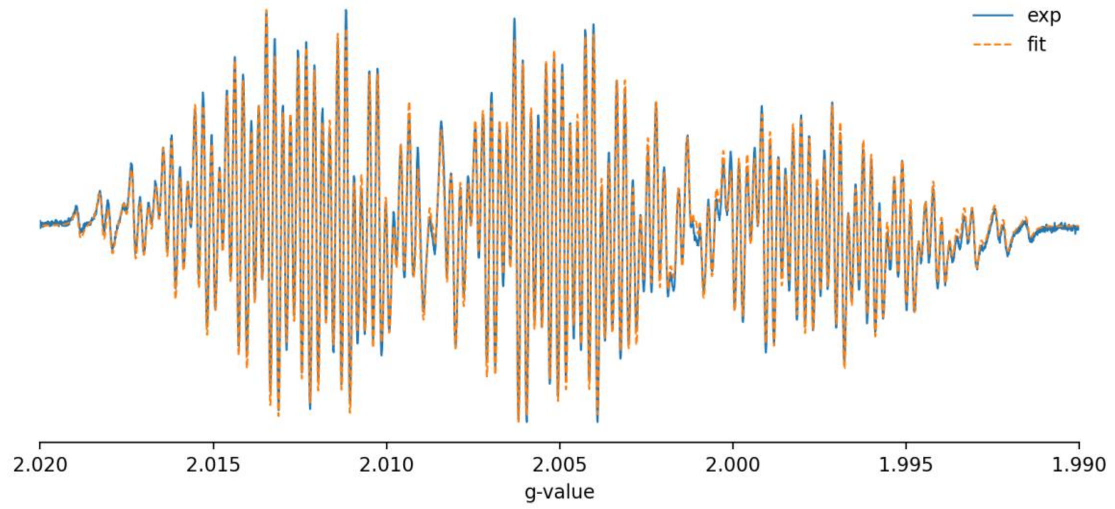

Figure S11. Comparison of normalised measured (solid line) and fitted (dashed line) cw-EPR spectra of [$\mathbf{1}^{\bullet-}$], at room temperature. 400 μL of a 15 mM solution of nitrobenzene in benzene were mixed with 300 μL of NaHMDS 100 mM in benzene. Measured with 30 dB attenuation, 0.1 Gauss modulation amplitude and 1 scan. Fitting parameters are given in Table S1. Note that the employed model required using two equivalent Na ions.

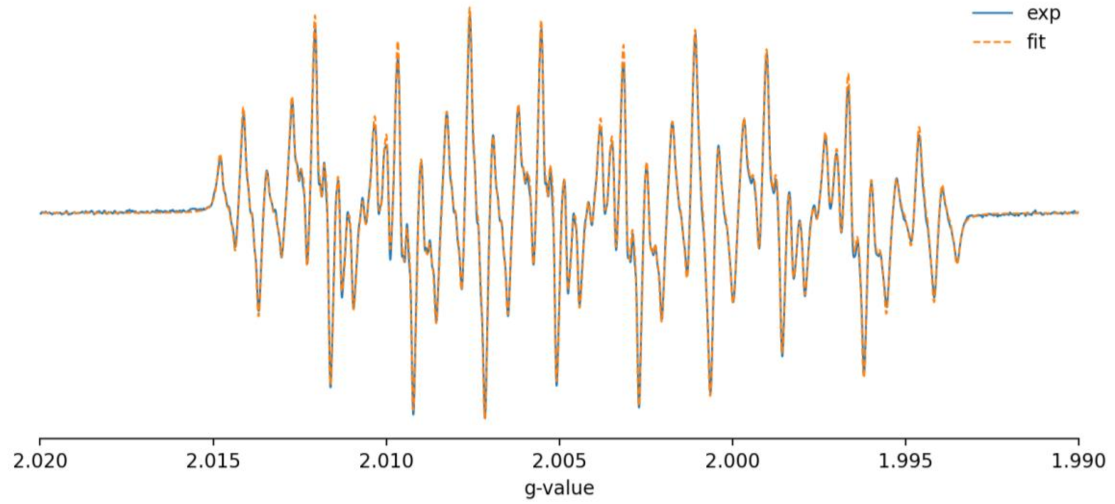

Figure S12. Comparison of normalised measured (solid line) and fitted (dashed line) cw-EPR spectra of [$\mathbf{1}^{\bullet-}$], at room temperature. 100 μL of a 40 mM solution of nitrobenzene in THF were mixed with 25 μL of NaHMDS 80 mM in THF and 50μL of 15-crown ether 80 mM in THF. Measured with 20 dB attenuation, 0.1 Gauss modulation amplitude and 3 scans. Fitting parameters are given in Table S1. Note that the employed model required using just one Na ion.

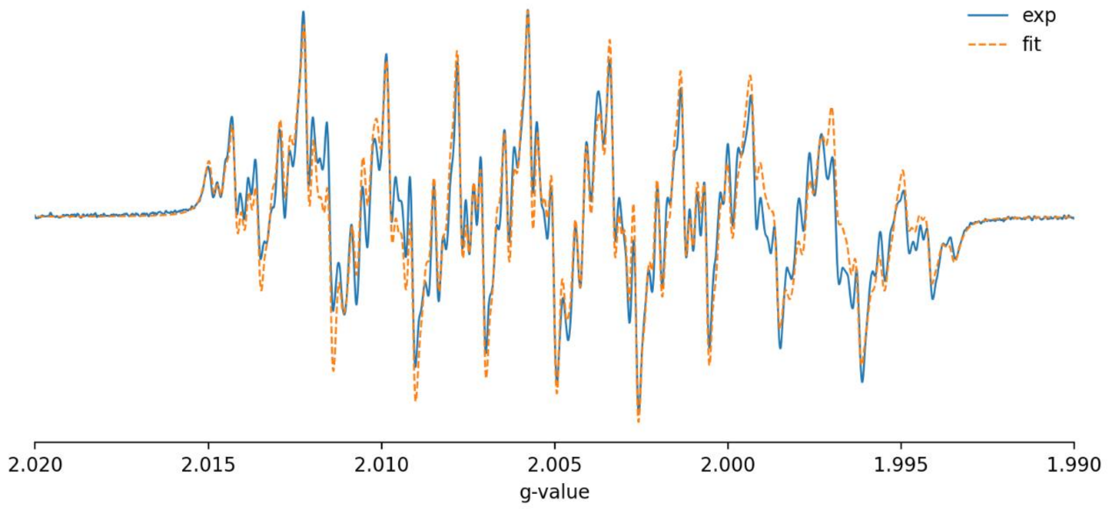

Figure S13. Comparison of normalised measured (solid line) and fitted (dashed line) cw-EPR spectra of [$\mathbf{1}^{\bullet-}$], at room temperature. 100 μL of a 40 mM solution of nitrobenzene in THF were mixed with 50 μL of NaHMDS 80 mM in THF and 50μL of [2.2.2.]-Cryptand 80 mM in THF. Measured with 20 dB attenuation, 0.1 Gauss modulation amplitude and 3 scans. Fitting parameters are given in Table S1. Note that the employed model required using just one Na ion, but the associated HFC is comparatively larger than in other cases. Attempts to fit the data with more than one Na ion failed.

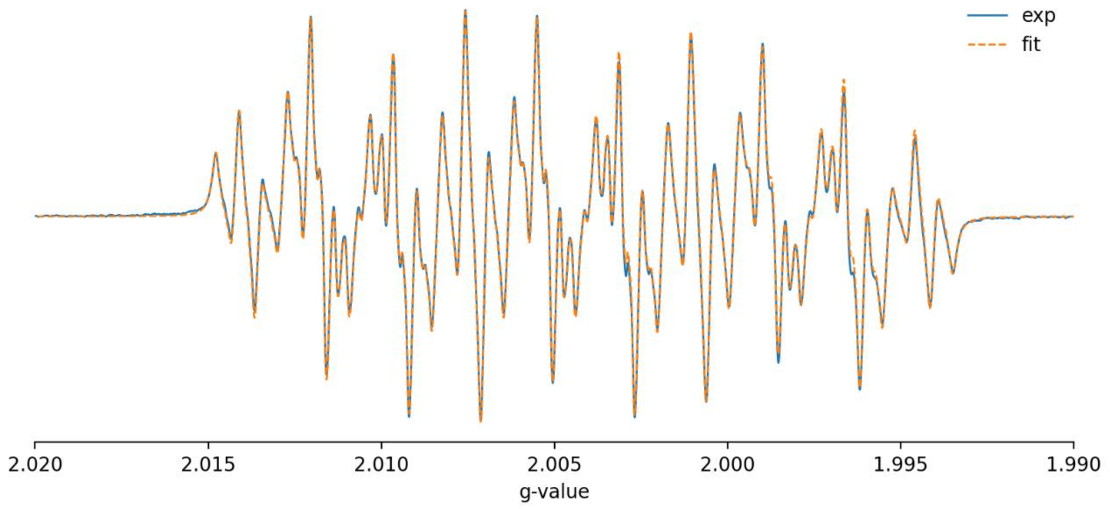

Figure S14. Comparison of normalised measured (solid line) and fitted (dashed line) cw-EPR spectra of [$\mathbf{1}^{\bullet-}$], at room temperature. 100 μL of a 40 mM solution of nitrobenzene in THF were mixed with 12.5 μL of Na(^{iPr}Cp) 80 mM in THF and 50μL of 15-crown ether 80 mM THF. Measured with 20 dB attenuation, 0.1 Gauss modulation amplitude and 3 scans. Fitting parameters are given in Table S1.

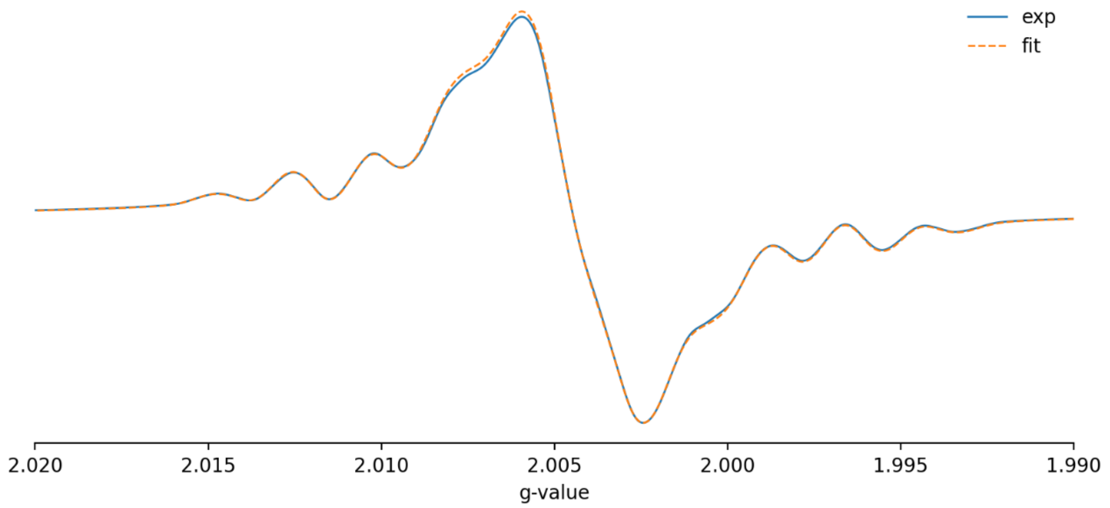

Figure S15. Comparison of normalised measured (solid line) and fitted (dashed line) cw-EPR spectra of [**1**$^{\bullet-}$], at room temperature. 100 μL of a 40 mM solution of nitrobenzene in THF were mixed with 50 μL of KO^{t}Bu 80 mM in THF. Measured with 20 dB attenuation, 1 G modulation amplitude and 1 scan. Fitting parameters are given in Table S1.

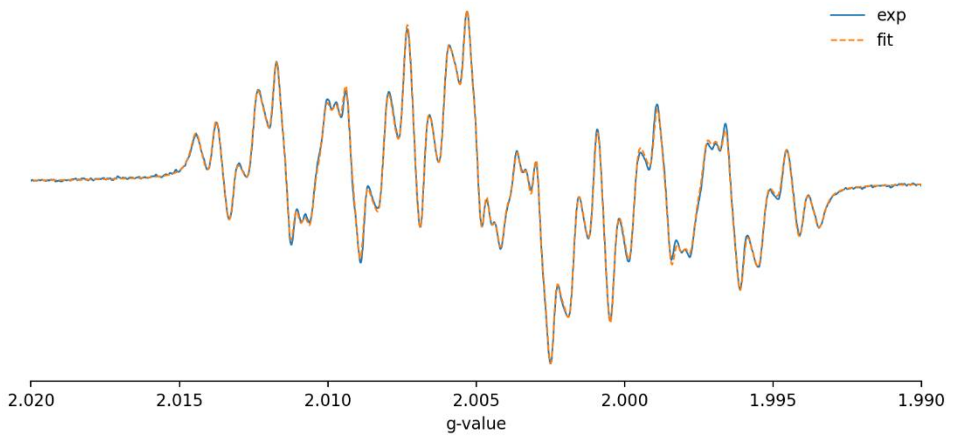

Figure S16. Comparison of normalised measured (solid line) and fitted (dashed line) cw-EPR spectra of [**1**$^{\bullet-}$], at room temperature. 100 μL (95 μL of THF plus 5 μL of DMSO) of a 10 mM solution of nitrobenzene were mixed with 50 μL of KO^{t}Bu 80 mM in THF. Measured with 25 dB attenuation, 0.1 G modulation amplitude and 15 scans. Fitting parameters are given in Table S1.

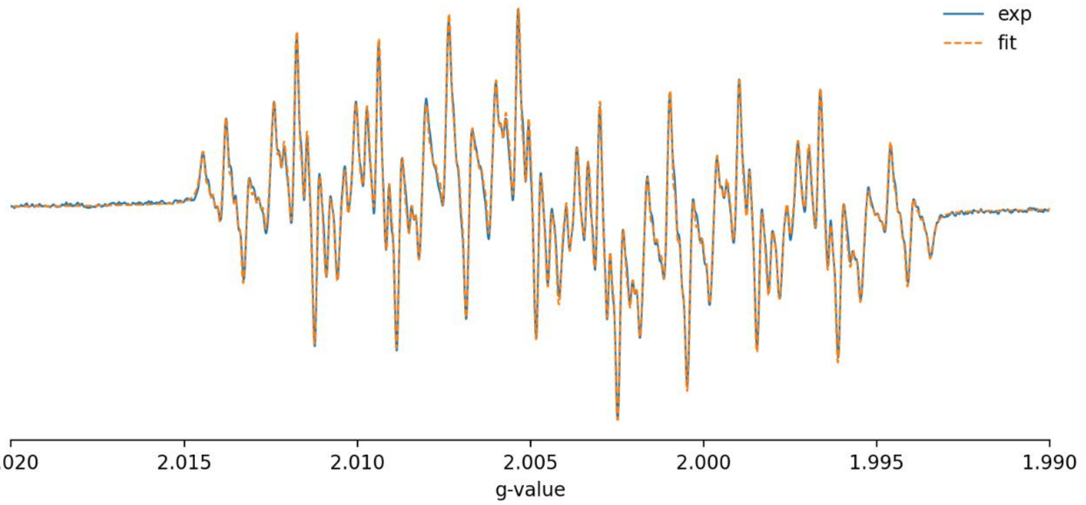

Figure S17. Comparison of normalised measured (solid line) and fitted (dashed line) cw-EPR spectra of [$\mathbf{1}^{\bullet-}$], at room temperature. 100 μL (98 μL of THF plus 2 μL of DMSO) of a 5 mM solution of nitrobenzene were mixed with 50 μL of KO^{t}Bu 80 mM in THF. Measured with 25 dB attenuation, 0.1 G modulation amplitude and 15 scans. Fitting parameters are given in Table S1.

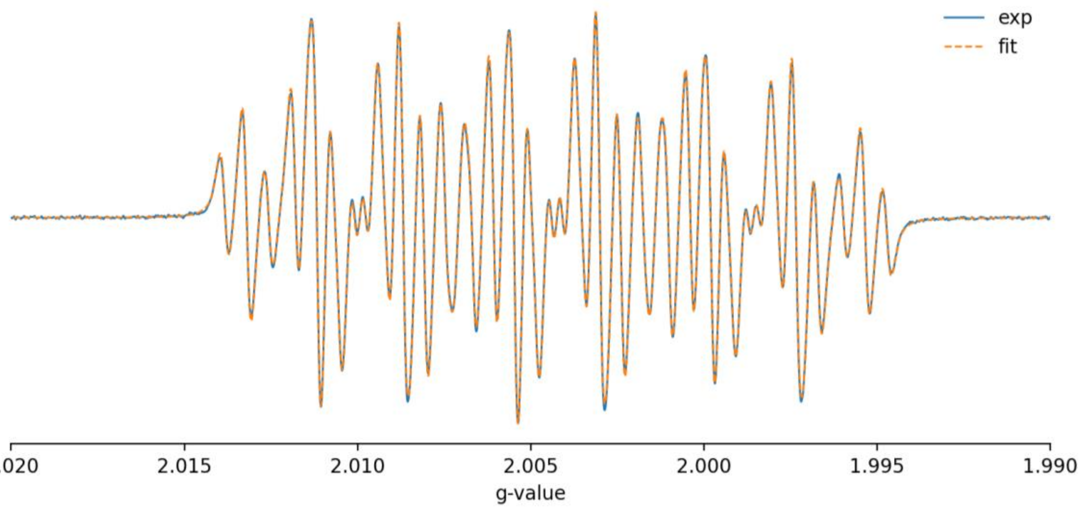

Figure S18. Comparison of normalised measured (solid line) and fitted (dashed line) cw-EPR spectra of [$\mathbf{1}^{\bullet-}$], at room temperature. 100 μL of a 10 mM solution of nitrobenzene in THF were mixed with 12.5 μL of KO^{t}Bu 80 mM in THF and 12.5 μL of [2.2.2.]-Cryptand 80 mM in THF. Measured with 20 dB attenuation, 0.1 G modulation amplitude and 1 scan. Fitting parameters are given in Table S1.

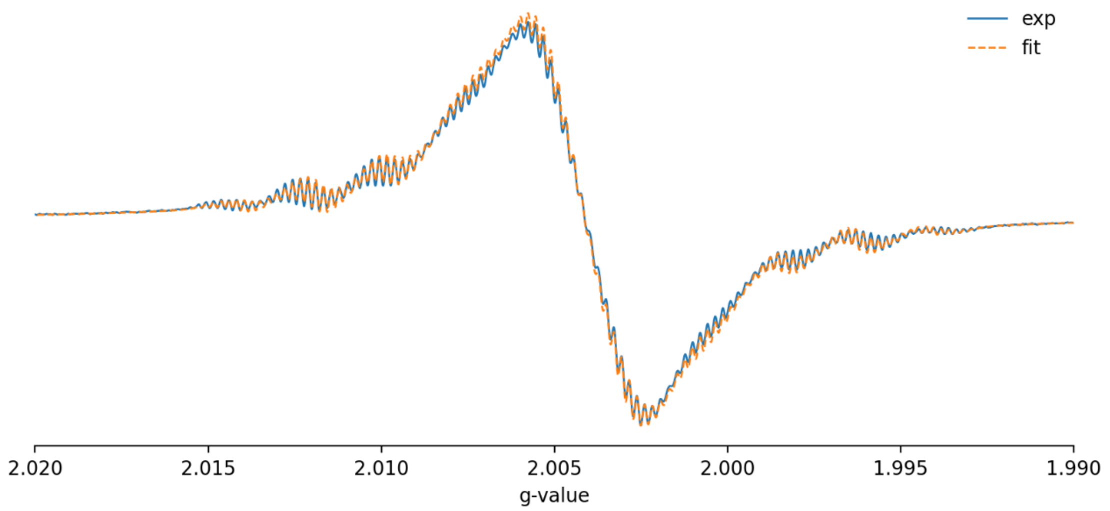

Figure S19. Comparison of normalised measured (solid line) and fitted (dashed line) cw-EPR spectra of [**1**$^{•-}$], at room temperature. 100 μL of a 40 mM solution of nitrobenzene in THF were mixed with 50 μL of KHMDS 80 mM in THF. Measured with 20 dB attenuation, 0.1 G modulation amplitude and 5 scan. Fitting parameters are given in Table S1. Note that the HFC to K^+ ion is comparatively large, but this is assigned to the broad spectral resolution.

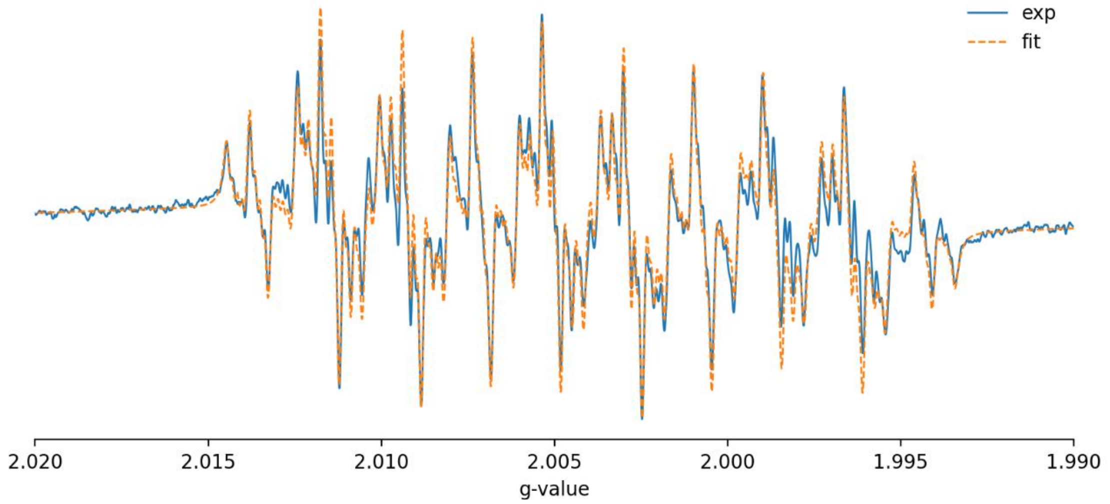

Figure S20. Comparison of normalised measured (solid line) and fitted (dashed line) cw-EPR spectra of [**1**$^{•-}$], at room temperature. 100 μL of a 5 mM solution of nitrobenzene in THF were mixed with 6.25 μL of KHMDS 80 mM in THF. Measured with 25 dB attenuation, 0.1 G modulation amplitude and 30 scans. Fitting parameters are given in Table S1.

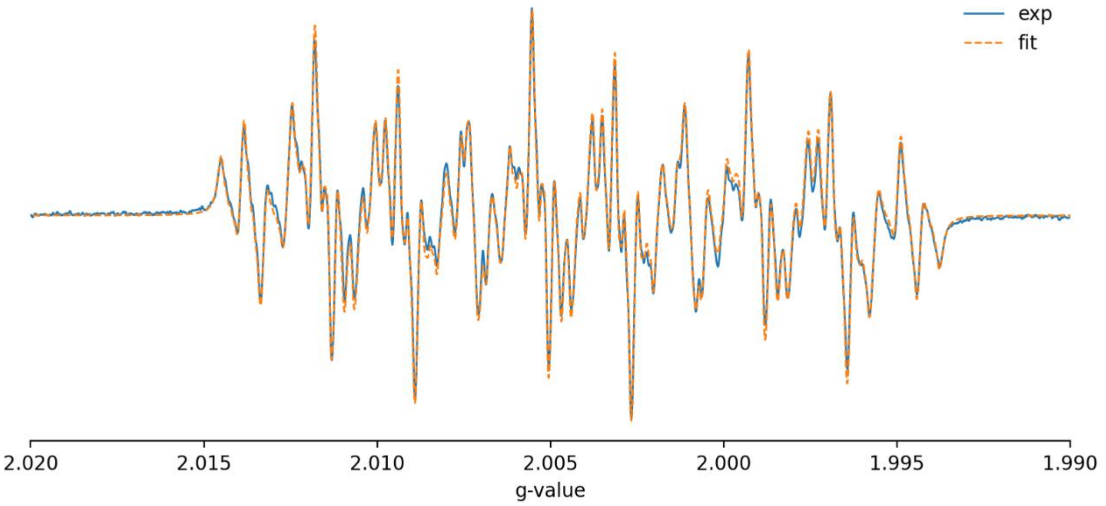

Figure S21. Comparison of normalised measured (solid line) and fitted (dashed line) cw-EPR spectra of [**1**$^{•-}$], at room temperature. 100 μL of a 10 mM solution of nitrobenzene in THF were mixed with 12.5 μL of KHMDS 80 mM in THF and 12.5 μL of 15-Crown ether 80 mM in THF. Measured with 20 dB attenuation, 0.1 G modulation amplitude and 3 scans. Fitting parameters are given in Table S1.

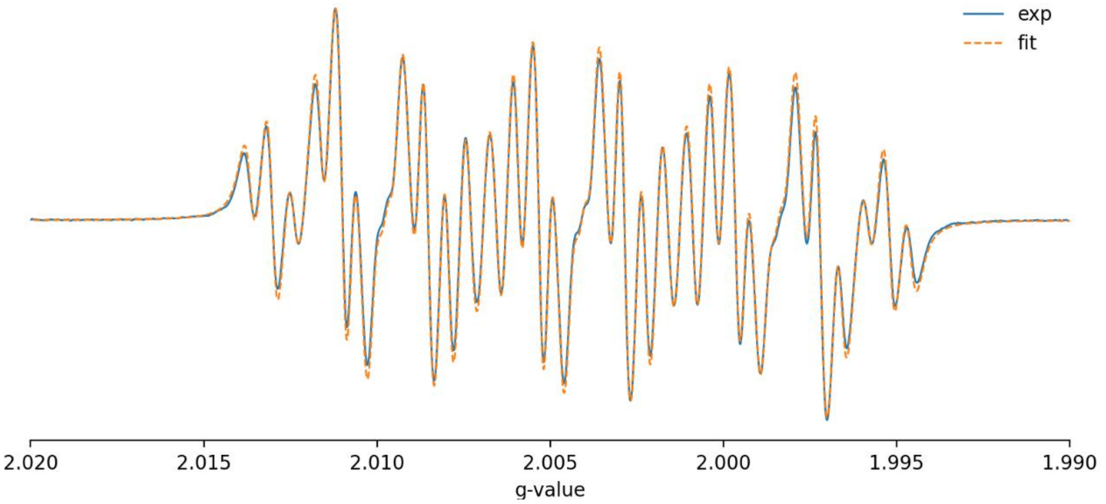

Figure S22. Comparison of normalised measured (solid line) and fitted (dashed line) cw-EPR spectra of [**1**$^{•-}$], at room temperature. A solution used to obtain crystals, which consisted of a 35 mM solution of **[1]** mixed in an equimolar ratio with 80 mM Crypt-222, and 80 mM KHMDS 80, was diluted into 10 mM. From this, we took 100 μL. Measured with 25 dB attenuation, 0.1 G modulation amplitude and 30 scans. Fitting parameters are given in Table S1.

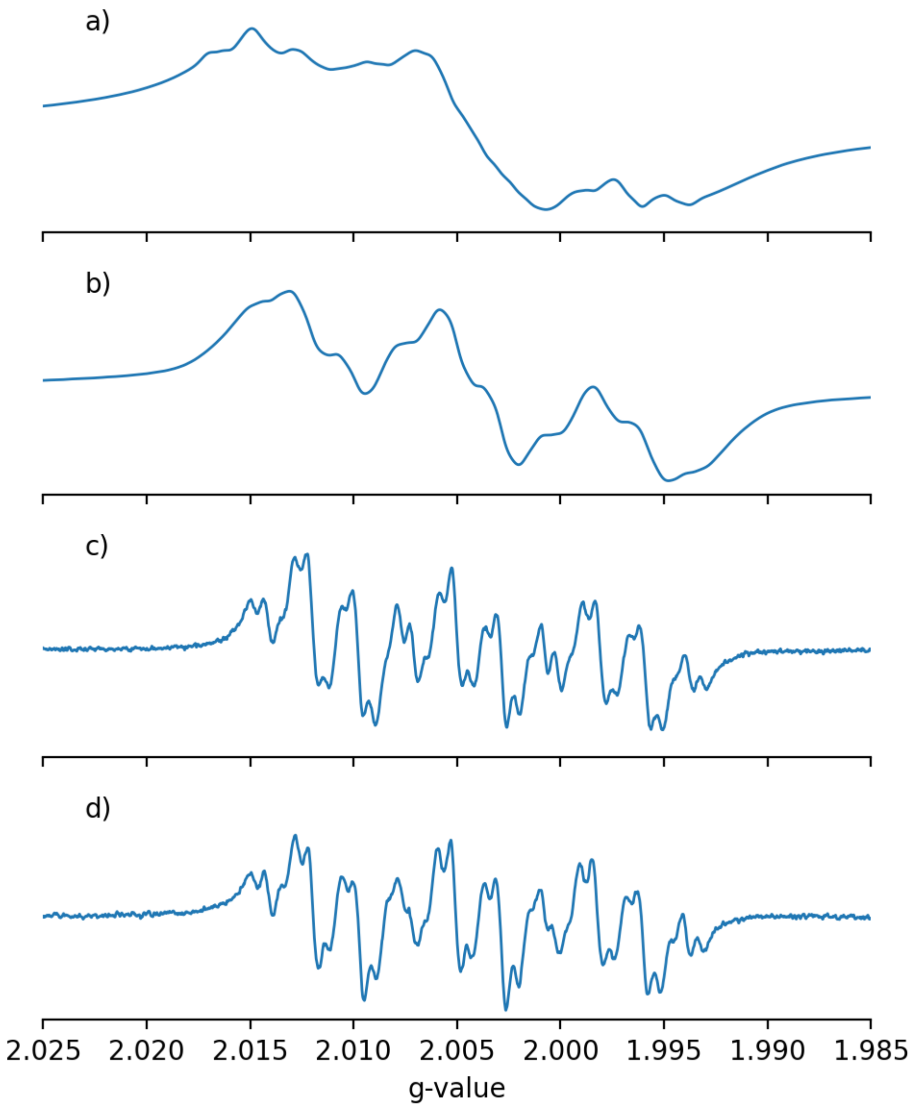

Figure S23. Comparison of selected normalised cw-EPR spectra of [$\mathbf{1}^{\bullet-}$] formed with ^{t}BuLi, at room temperature. a) 100 μL of a 40 mM solution of nitrobenzene in benzene were mixed with 2.35 μL of ^{t}BuLi 1.7M in *n*-pentane – measured with 20 dB attenuation, 1 G modulation amplitude and 1 scan. b) 100 μL of a 40 mM solution of nitrobenzene in THF were mixed with 2.35 μL of ^{t}BuLi 1.7M in *n*-pentane – measured with 20 dB attenuation, 1 G modulation amplitude and 1 scan. c) 100 μL (85 μL of benzene plus 15 μL of DMSO) of a 40 mM solution of nitrobenzene were mixed with 2.35 μL of ^{t}BuLi 1.7M in *n*-pentane – measured with 27 dB attenuation, 0.1 G modulation amplitude and 7 scans. d) 100 μL (85 μL of THF plus 15 μL of DMSO) of a 40 mM solution of nitrobenzene were mixed with 2.35 μL of ^{t}BuLi 1.7M in *n*-pentane – measured with 30 dB attenuation, 0.1 G modulation amplitude and 20 scans. The fit of this spectrum is presented in Figure S1.

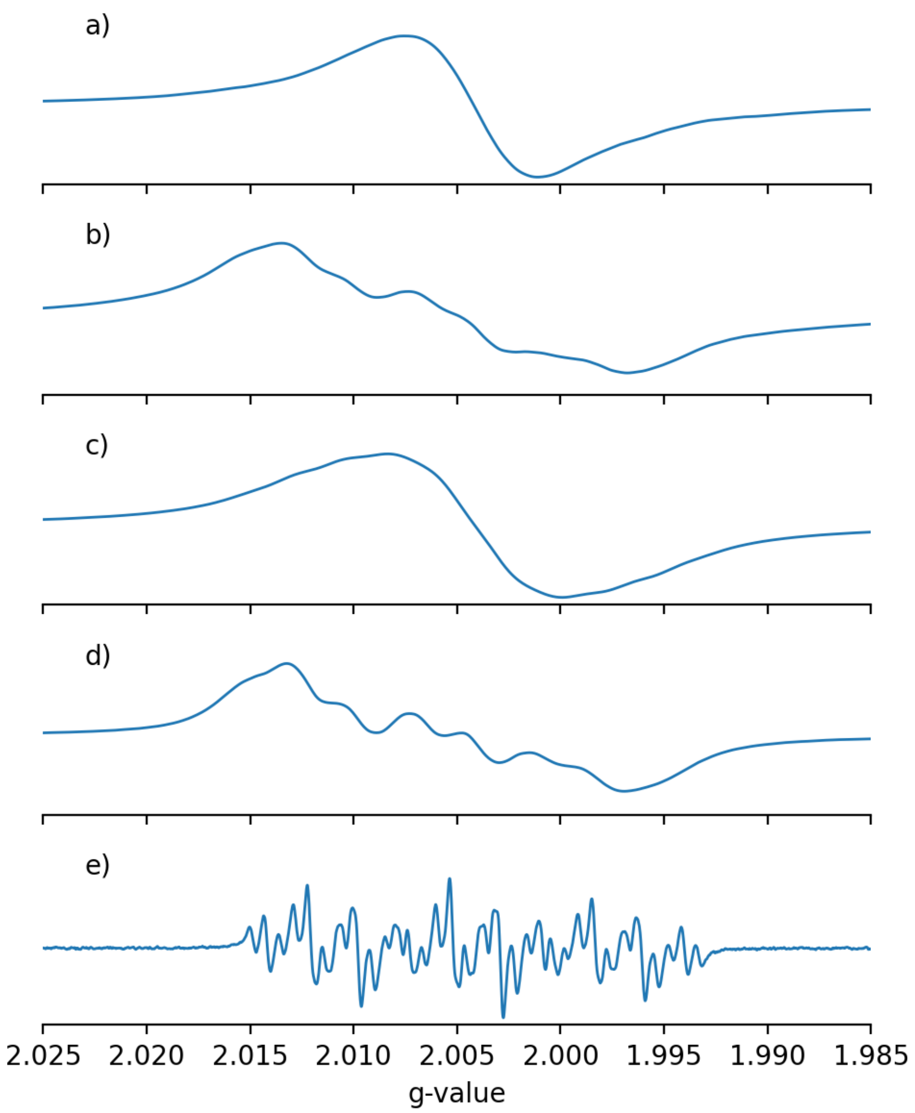

Figure S24. Comparison of selected normalised cw-EPR spectra of [**1**$^{•-}$] formed with LDA, at room temperature. a) 100 μL of a 40 mM solution of nitrobenzene in benzene were mixed with 50 μL of LDA 80 mM in benzene (molar ratio 1:1) – measured with 20 dB attenuation, 1 G modulation amplitude and 1 scan. b) 100 μL of a 40 mM solution of nitrobenzene in benzene were mixed with 250 μL of LDA 80 mM in benzene (molar ratio 1:5) – measured with 20 dB attenuation, 1 G modulation amplitude and 1 scan. c) 100 μL of a 40 mM solution of nitrobenzene in THF were mixed with 50 μL of LDA 80 mM in THF (molar ratio 1:1) – measured with 20 dB attenuation, 1 G modulation amplitude and 1 scan. d) 100 μL of a 40 mM solution of nitrobenzene in THF were mixed with 250 μL of LDA 80 mM in THF (molar ratio 1:5) – measured with 20 dB attenuation, 1 G modulation amplitude and 1 scan. e) 100 μL (75 μL of THF plus 25 μL of DMSO) of a 40 mM solution of nitrobenzene were mixed with 50 μL of LDA 80 mM in THF – measured with 30 dB attenuation, 0.1 G modulation amplitude and 35 scans. The fit of this spectrum is presented in Figure S2.

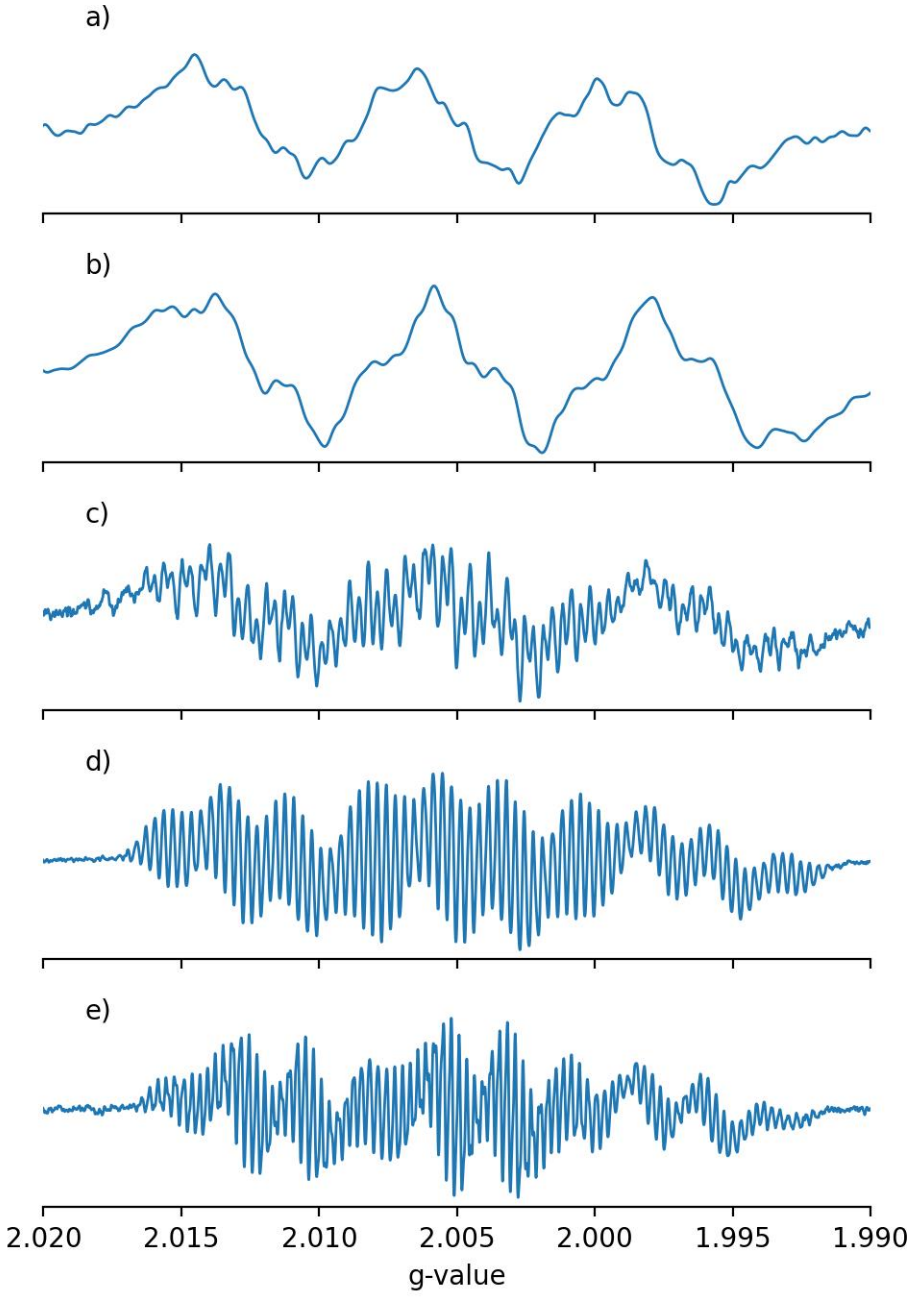

Figure S25. Comparison of selected normalised cw-EPR spectra of [**1**$^{\bullet-}$] formed with LiO^{t}Bu, at room temperature. a) 100 μL of a 40 mM solution of nitrobenzene in THF were mixed with 4 μL of LiO^{t}Bu 1.0 M in THF (molar ratio 1:1) – measured with 20 dB attenuation, 1 G modulation amplitude and 5 scans. b) 100 μL of a 40 mM solution of nitrobenzene in benzene were mixed with 4 μL of LiO^{t}Bu 1.0 M in THF (molar ratio 1:1) – measured with 20 dB attenuation, 1 G modulation amplitude and 5 scans. c) 100 μL of a 40 mM solution of nitrobenzene in benzene were mixed with 4 μL of LiO^{t}Bu 1.0 M in THF (molar ratio 1:1) – measured with 20 dB attenuation, 0.1 G modulation amplitude and 10 scans. d) 100 μL of a 40 mM solution of nitrobenzene in benzene were mixed with 20 μL of LiO^{t}Bu 1.0 M in THF (molar ratio 1:5) – measured with 20 dB attenuation, 0.1 G modulation amplitude and 5 scans. The fit of this spectrum is presented in Figure S3 e) 100 μL (95 μL of benzene plus 5 μL of DMSO) of a 40 mM solution of nitrobenzene were mixed with 4 μL of LiO^{t}Bu 1.0 M in THF (molar ratio 1:1) – measured with 20 dB attenuation, 0.1 G modulation amplitude and 5 scans. The fit of this spectrum is presented in Figure S4.

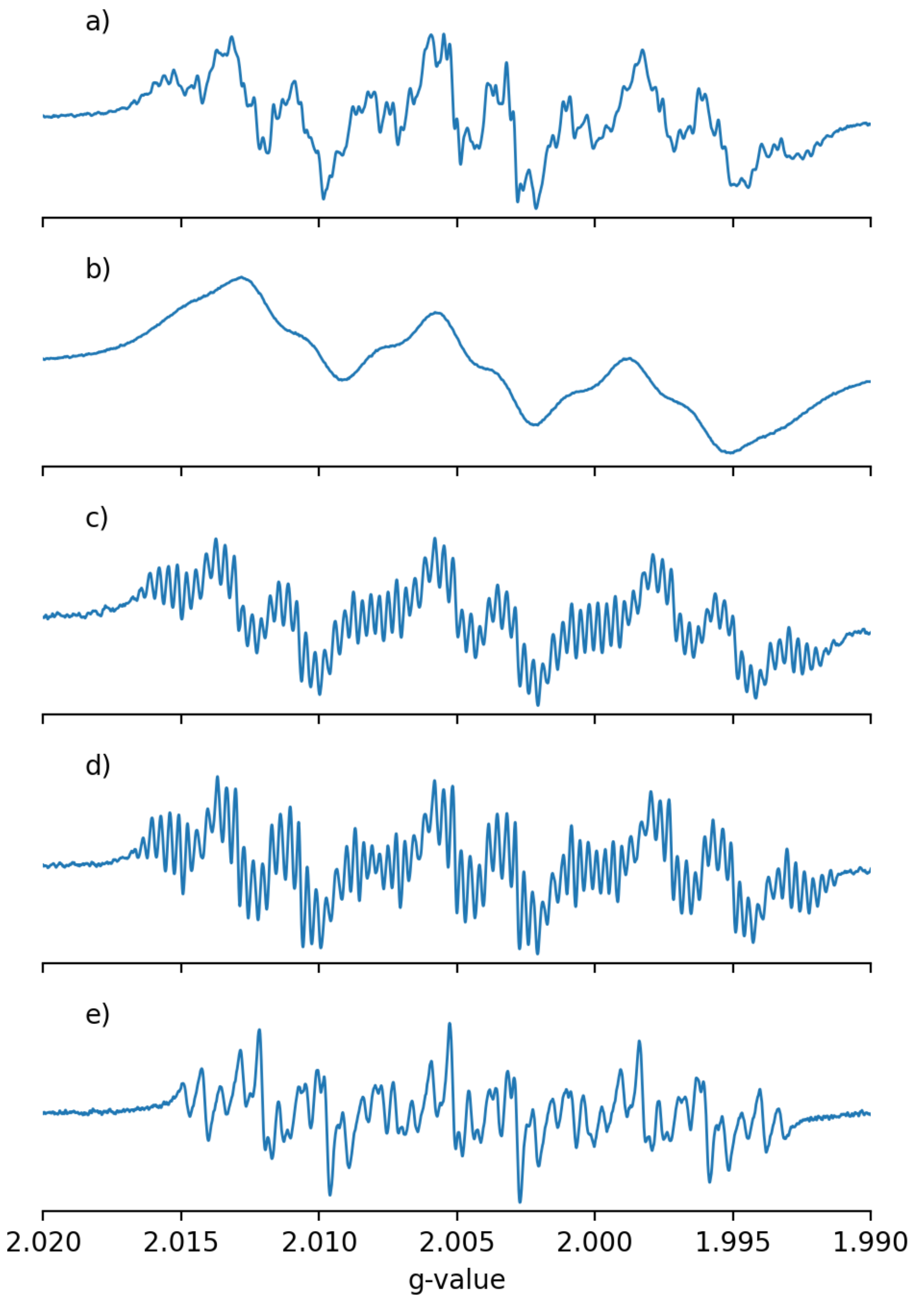

Figure S26. Comparison of selected normalised cw-EPR spectra of [**1**$^{\bullet-}$] formed with LiHMDS, at room temperature. a) 100 μL of a 40 mM solution of nitrobenzene in THF were mixed with 4 μL of LiHMDS 1.0 M in THF (molar ratio 1:1) – measured with 20 dB attenuation, 0.1 G modulation amplitude and 10 scans. b) 100 μL (95 μL of THF plus 5 μL of DMSO) of a 40 mM solution of nitrobenzene were mixed with 4 μL of LiHMDS 1.0 M in THF (molar ratio 1:1) – measured with 20 dB attenuation, 0.1 G modulation amplitude and 4 scans. c) 100 μL of a 40 mM solution of nitrobenzene in benzene were mixed with 4 μL of LiHMDS 1.0 M in THF (molar ratio 1:1) – measured with 20 dB attenuation, 0.1 G modulation amplitude and 30 scans. d) 100 μL of a 40 mM solution of nitrobenzene in benzene were mixed with 20 μL of LiHMDS 1.0 M in THF (molar ratio 1:5) – measured with 30 dB attenuation, 0.1 G modulation amplitude and 25 scans. The fit of this spectrum is presented in Figure S5. e) 100 μL (95 μL of THF plus 5 μL of DMSO) of a 40 mM solution of nitrobenzene were mixed with 2 μL of LiHMDS 1.0 M in THF (molar ratio 1:0.5) – measured with 25 dB attenuation, 0.1 G modulation amplitude and 3 scans. The fit of this spectrum is presented in Figure S6.

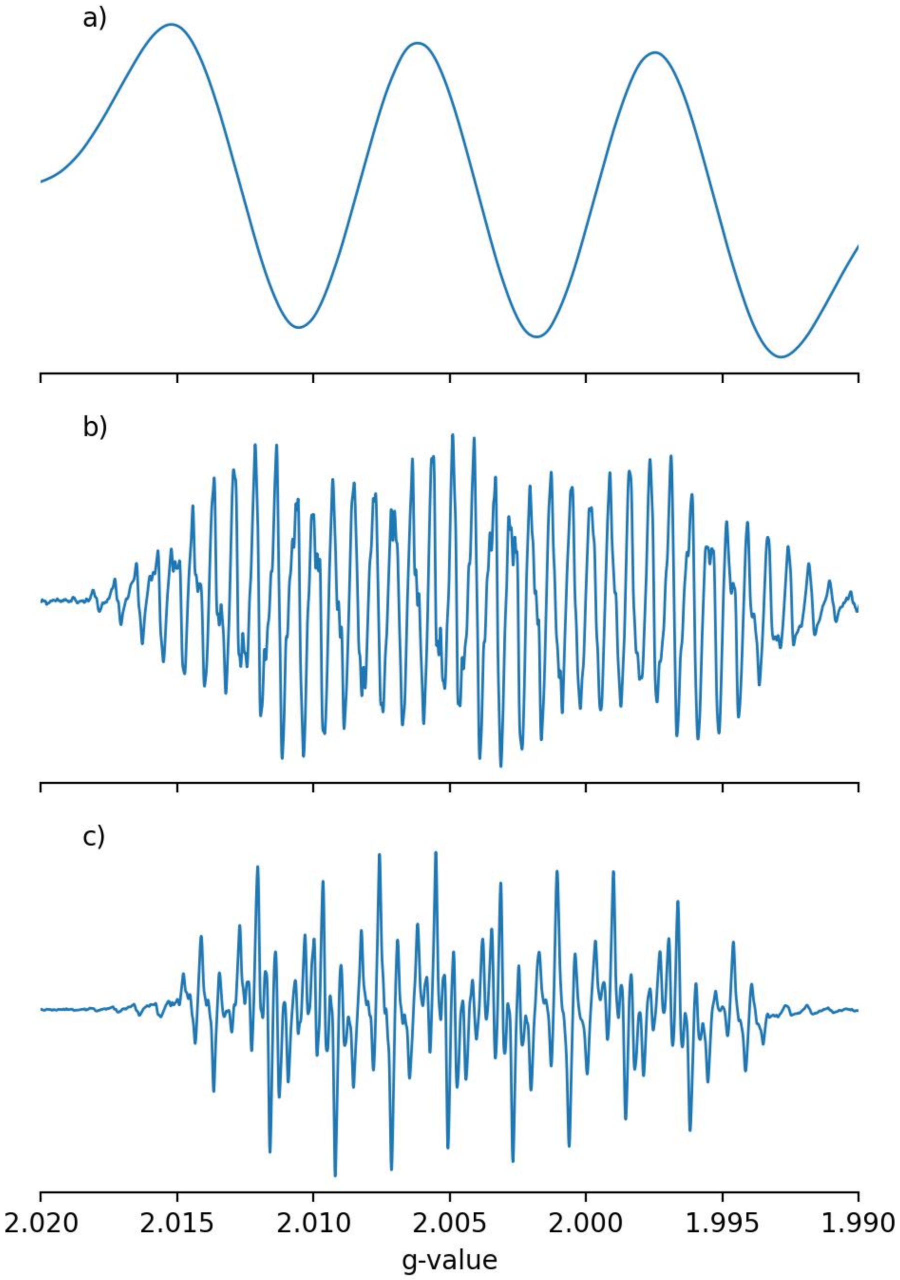

Figure S27. Comparison of selected normalised cw-EPR spectra of [**1**$^{•-}$] formed with NaO^{t}Bu, at room temperature. a) 100 μL of a 40 mM solution of nitrobenzene in benzene were mixed with 50 μL of NaO^{t}Bu 80 mM in benzene (molar ratio 1:1) – measured with 20 dB attenuation, 1 G modulation amplitude and 1 scan. The fit of this spectrum is presented in Figure **S7**. b) 100 μL of a 40 mM solution of nitrobenzene in THF were mixed with 50 μL of NaO^{t}Bu 80 mM in THF (molar ratio 1:1) – measured with 15 dB attenuation, 0.1 G modulation amplitude and 5 scans. The fit of this spectrum is presented in Figure **S8**. c) 100 μL of a 40 mM solution of nitrobenzene in THF were mixed with 25 μL of NaO^{t}Bu 80 mM in THF (molar ratio 1:0.5) and 50 μL of 15-crown ether 80 mM in THF – measured with 20 dB attenuation, 0.1 Gauss modulation amplitude and 8 scans. The fit of this spectrum is presented in Figure **S9**.

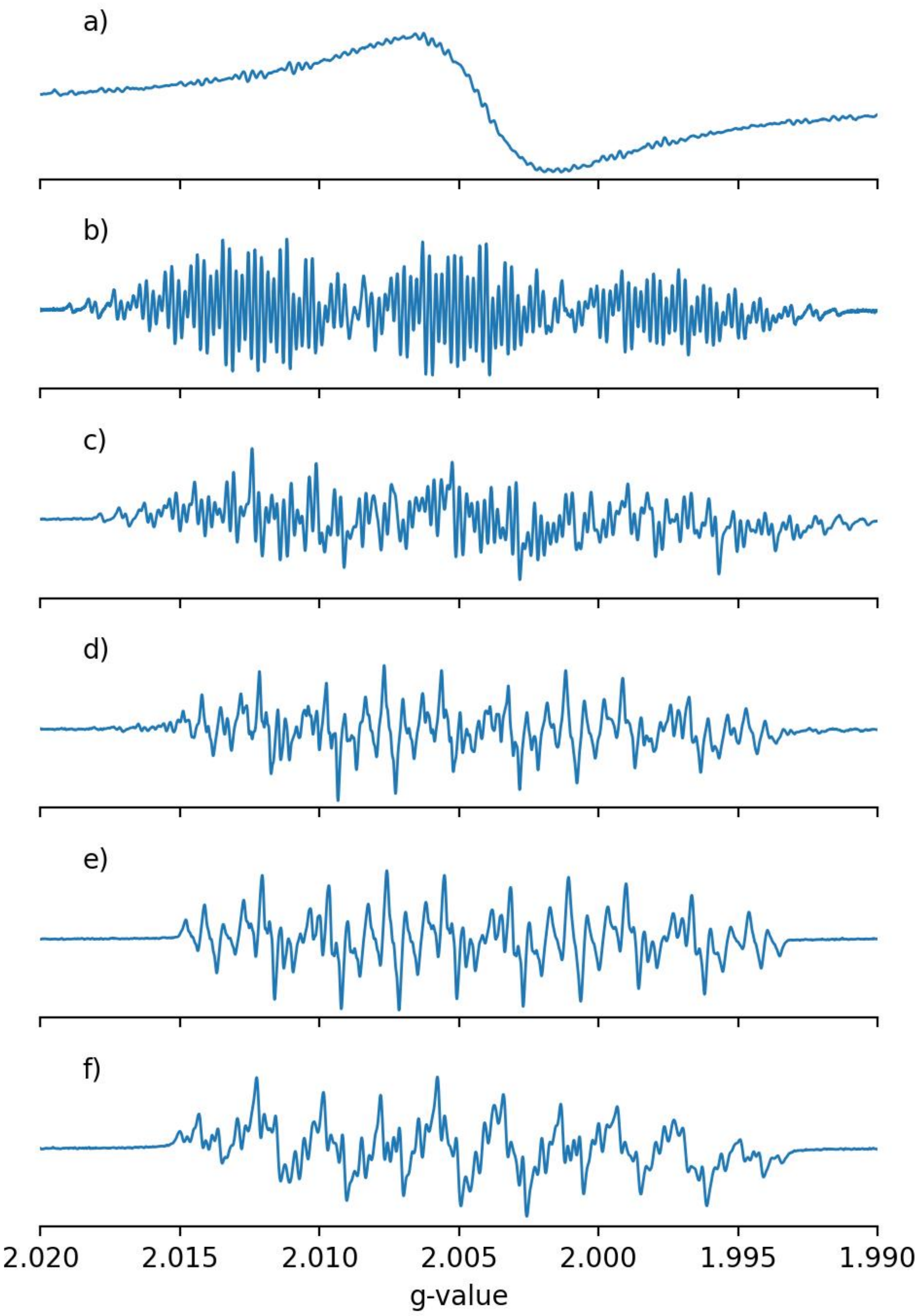

Figure S28. Comparison of selected normalised cw-EPR spectra of [**1**$^{\bullet-}$] formed with NaHMDS, at room temperature. a) 100 μL of a 7 mM solution of nitrobenzene in benzene were mixed with 12.5 μL of NatButOx 80 mM in benzene (molar ratio 1:0.5) – measured with 30 dB attenuation, 0.1 G modulation amplitude and 50 scans. b) 400 μL of a 15 mM solution of nitrobenzene in benzene were mixed with 300 μL of NaHMDS 100 mM in benzene (ratio 1:1) – measured with 30 dB attenuation, 0.1 Gauss modulation amplitude and 1 scan. The fit of this spectrum is presented in Figure S11 c) 100 μL of a 40 mM solution of nitrobenzene in THF were mixed with 50 μL of NaHMDS 80 mM in THF (molar ratio 1:1) – measured with 20 dB attenuation, 0.1 G modulation amplitude and 5 scans. The fit of this spectrum is presented in Figure S10. d) 100 μL of a 40 mM solution of nitrobenzene in THF were mixed with 50 μL of NaHMDS 80 mM in THF (molar ratio 1:1) and 50 μL of 15-crown ether 80 mM in THF – measured with 20 dB attenuation, 0.1 Gauss modulation amplitude and 4 scans. e) 100 μL of a 40 mM solution of nitrobenzene in THF were mixed with 25 μL of NAHMDS 80 mM in THF (molar ratio 1:0.5) and 50 μL of 15-crown ether 80 mM in THF – measured with 20 dB attenuation, 0.1 Gauss modulation amplitude and 3 scans. The fit of this spectrum is presented in Figure **S12**. f) 100 μL of a 40 mM solution of nitrobenzene in THF were

mixed with 50 μL of NAHMDS 80 mM in THF (molar ratio 1:1) and 50 μL of [2.2.2]-Cryptand 80 mM in THF – measured with 20 dB attenuation, 0.1 Gauss modulation amplitude and 3 scans. The fit of this spectrum is presented in Figure **S13**.

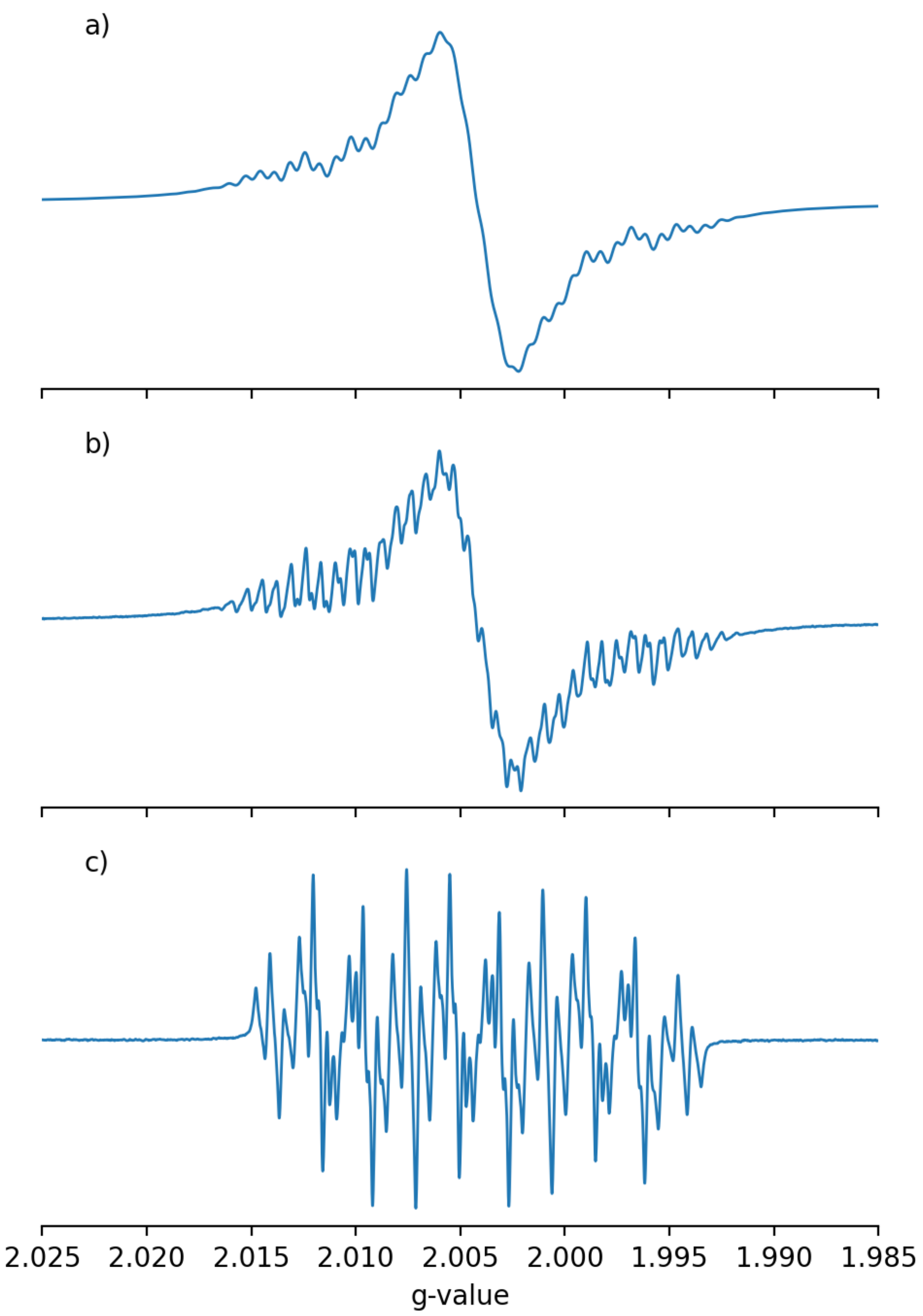

Figure S29. Comparison of selected normalised cw-EPR spectra of [$\mathbf{1}^{\bullet-}$] formed with Na(^{iPr}Cp), at room temperature. a) 100 μL of a 40 mM solution of nitrobenzene in THF were mixed with 50 μL of Na(^{iPr}Cp) 80 mM in THF (molar ratio 1:1) – measured with 20 dB attenuation, 1 G modulation amplitude and 1 scan. b) 100 μL of a 40 mM solution of nitrobenzene in THF were mixed with 50 μL of Na(^{iPr}Cp) 80 mM in THF (molar ratio 1:1) – measured with 20 dB attenuation, 0.1 G modulation amplitude and 5 scans. c) 100 μL of a 40 mM solution of nitrobenzene in THF were mixed with 12.5 μL of Na(^{iPr}Cp) 80 mM in THF (molar ratio 1:0.25) and 50 μL of 15-crown ether 80 mM in THF – measured with 20 dB attenuation, 0.1 Gauss modulation amplitude and 10 scans. The fit of this spectrum is presented in Figure S14.

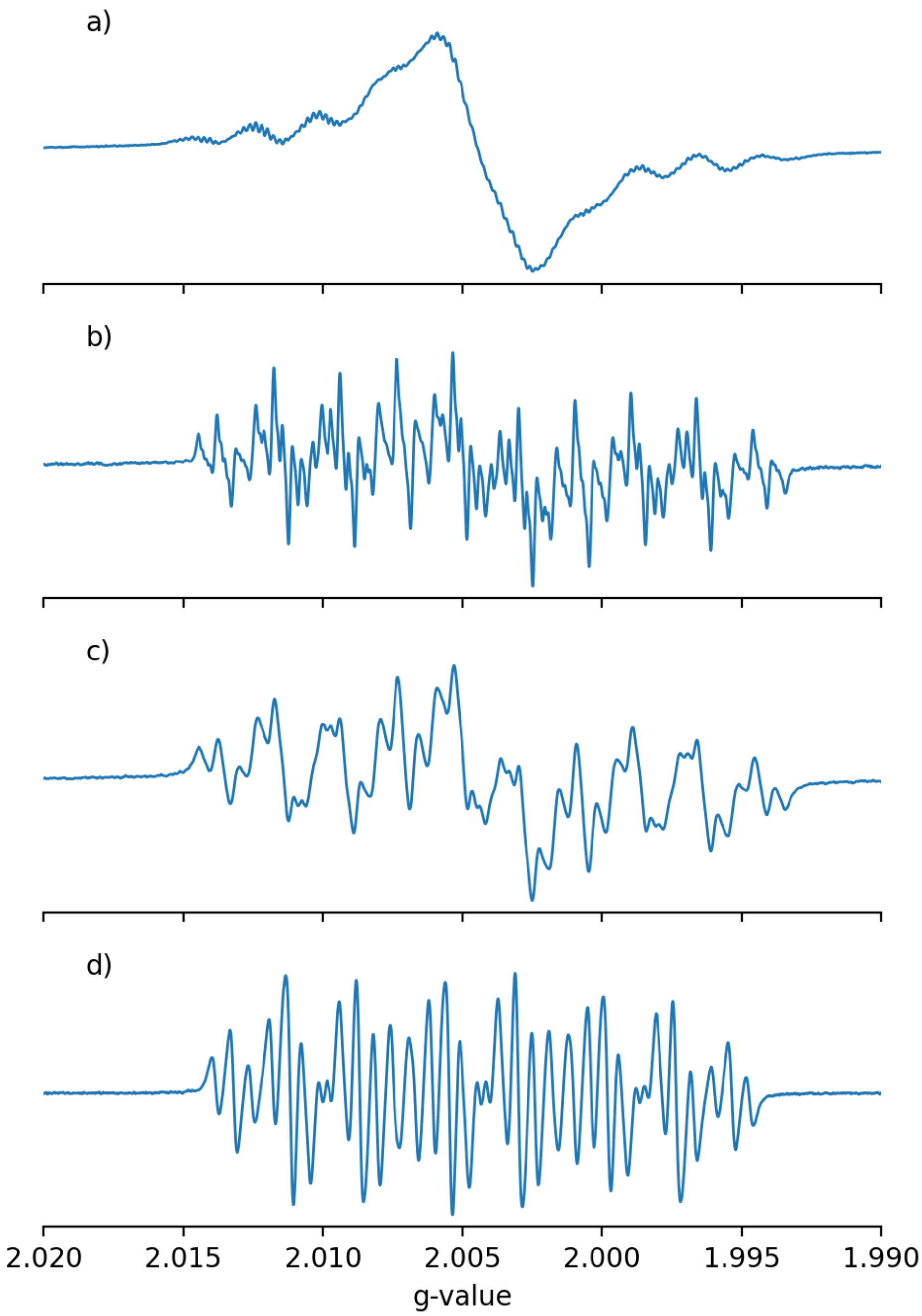

Figure S30. Comparison of selected normalised cw-EPR spectra of [**1**$^{\bullet-}$] formed with KO^{t}Bu, at room temperature. a) 100 μL of a 40 mM solution of nitrobenzene in THF were mixed with 50 μL of KO^{t}Bu 80 mM in THF (molar ratio 1:1) – measured with 20 dB attenuation, 0.1 G modulation amplitude and 5 scans. The fit of this spectrum is presented in Figure **S15**. b) 100 μL of a 5 mM solution of nitrobenzene in THF were mixed with 6.25 μL of KO^{t}Bu 80 mM in THF (molar ratio 1:1) – measured with 25 dB attenuation, 0.1 G modulation amplitude and 15 scans. The fit of this spectrum is presented in Figure S16. c) 100 μL (95 μL of THF plus 5 μL of DMSO) of a 10 mM solution of nitrobenzene were mixed with 12.5 μL of KO^{t}Bu 80 mM in THF (molar ratio 1:1) – measured with 25 dB attenuation, 0.1 G modulation amplitude and 15 scans. The fit of this spectrum is presented in Figure **S17**. d) 100 μL of a 10 mM solution of nitrobenzene in THF were mixed with 12.5 μL of [2.2.2.]-Cryptand 80 mM in THF and 12.5 μL of KO^{t}Bu 80 mM in THF (molar ratio 1:1) – measured with 20 dB attenuation, 0.1 G modulation amplitude and 1 scan. The fit of this spectrum is presented in Figure S18.

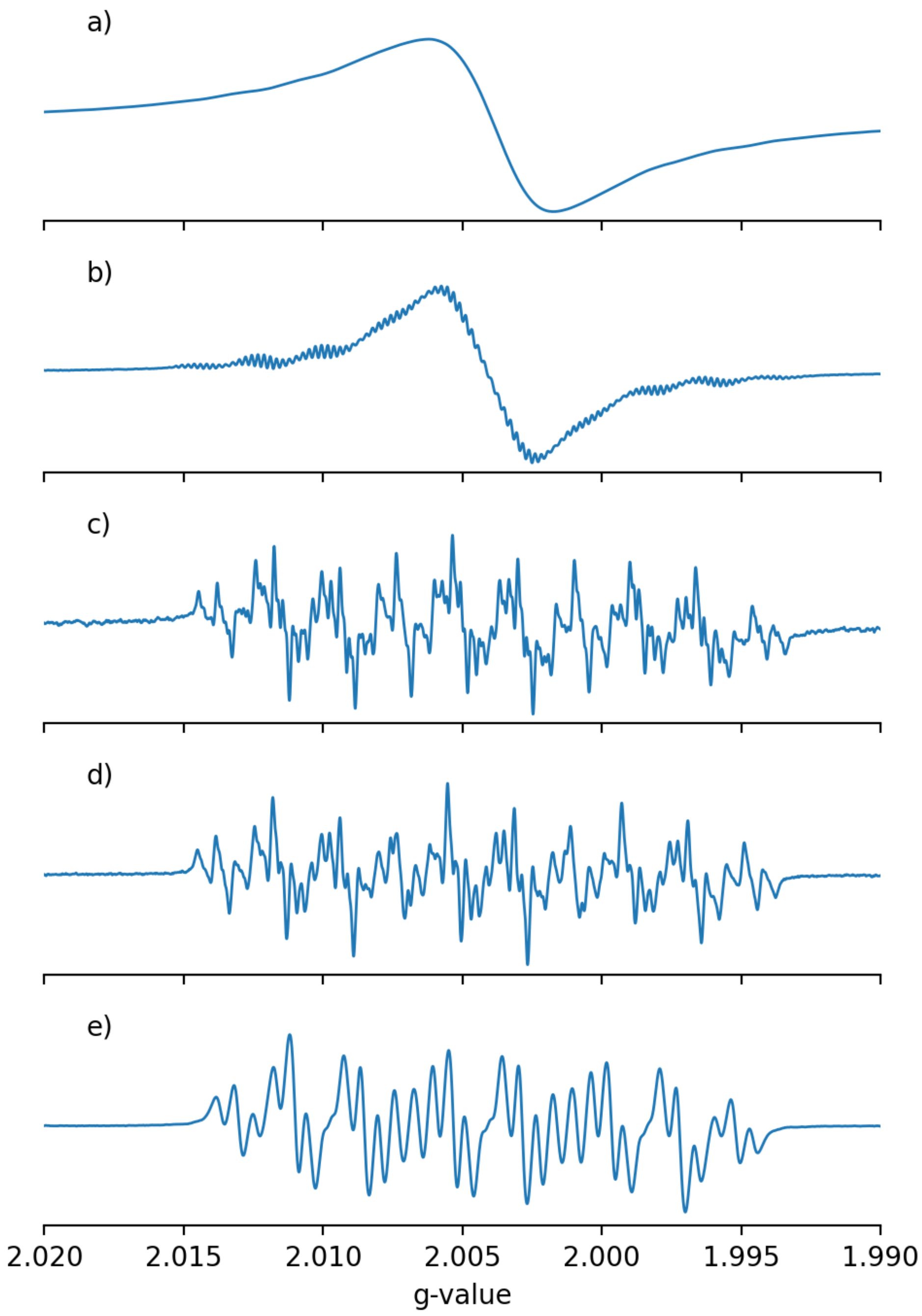

Figure S31. Comparison of selected normalised cw-EPR spectra of [$1^{•-}$] formed with KHMDS, at room temperature. a) 100 μL of a 40 mM solution of nitrobenzene in benzene were mixed with 50 μL of KHMDS 80 mM in benzene (molar ratio 1:1) – measured with 20 dB attenuation, 1 G modulation amplitude and 1 scan. b) 100 μL of a 40 mM solution of nitrobenzene in THF were mixed with 50 μL of KHMDS 80 mM in THF (molar ratio 1:1) – measured with 20 dB attenuation, 0.1 G modulation amplitude and 5 scans. The fit of this spectrum is presented in Figure **S19**. c) 100 μL of a 5 mM solution of nitrobenzene in THF were mixed with 6.25 μL of KHMDS 80 mM in THF (molar ratio 1:1) – measured with 25 dB attenuation, 0.1 G modulation amplitude and 30 scans. The fit of this spectrum is presented in Figure S20. d) 100 μL of a 10 mM solution of nitrobenzene in THF were mixed with 12.5 μL of 15-Crown ether 80 mM in THF and 12.5 μL of KHMDS 80 mM in THF (molar ratio 1:1) – measured with 20 dB attenuation, 0.1 G modulation amplitude and 3 scans. The fit of this spectrum is presented in Figure S21. e) A solution used to obtain crystals, which consisted of a 35 mM solution of **[1]** mixed in an equimolar ratio with 80 mM Crypt-222, and 80 mM KHMDS 80, was diluted into 10 mM. From this, we took 100 μL. Measured with 25 dB attenuation, 0.1 G modulation amplitude and 30 scans. The fit of this spectrum is presented in Figure S22.

Table S1. Comparison of model spin Hamiltonian parameters of $[\mathbf{1}^{\bullet-}]([\mathbf{X}^{+}])$ formed by different methods and conditions. When two solvents are given, values in parenthesis indicate %. Temperature (T), nitrobenzene concentration ([**1**]) and hyperfine coupling constants (A_x) are given in °C, milliMolar and Gauss, respectively. “Molar ratio” refers to the relative molar proportion of nitrobenzene and base. “Ratio” refers to the relative proportion of different species used in the fit. AcCN, DMF, DME, NH3, tButOH, Ace, HMPA, THF, C6H6 and DMSO stand for acetonitrile, Dimethyl formamide, 1,2 dimethyl ethane, liquid ammonia, *t*-butyl alcohol, Acetone, hexamethylphosphoramide, tetrahydrofuran, benzene and dimethyl sulfoxide, respectively. All fits have been obtained with Xenon Bruker software enabling second order effects and variable linewidths.

**Method**	**Solvent**	**Metal**	**Base**	***T***	**[1]**	**Molar ratio**	***g***	A_N	A_{H_p}	A_{H_o}	A_{H_m}	A_M	**Ratio**	**Ref**
Electrolytic	AcCN	n/a	n/a	23	-	-	2.0032	10.32	3.97	3.39	1.09	n/a	-	1
	DMF	n/a	n/a	23	-	-	n/a	9.70	4.03	3.36	1.07	n/a	-	2
Metal reduction	DME	K	n/a	23	-	-	n/a	10.33	3.86	3.46	1.13	n/a	-	3
	DME	Li	n/a	0	-	-	n/a	11.55	3.94	3.49	1.135	0.125	-	4
			n/a	-20	-	-	n/a	11.40	3.90	3.49	1.135	0.145	-	
		Na	n/a	23	-	-	n/a	11.60	4.08	3.56	1.12	0.40	-	
			n/a	-60	-	-	n/a	11.25	4.12	3.54	1.12	0.19	-	
		K	n/a	23	-	-	n/a	11.05	4.13	3.55	1.12	0.23	-	
			n/a	-60	-	-	n/a	10.60	4.16	3.55	1.12	0.18	-	
		Cs	n/a	0	-	-	n/a	10.80	4.16	3.54	1.12	2.95	-	
			n/a	-60	-	-	n/a	10.50	4.24	3.54	1.12	2.62	-	
	NH3	Li	n/a	-50	-	-	n/a	10.40	3.83	3.25	1.03	n/a	-	5
		Na	n/a	-50	-	-	n/a	10.63	3.82	3.24	1.03	n/a	-	
		K	n/a	-50	-	-	n/a	10.62	3.84	3.23	1.03	n/a	-	
	DME	Li	n/a	23	-	-	n/a	11.62	3.90	3.65	1.16	n/a	-	6
		Na	n/a	23	-	-	n/a	11.06	3.95	3.52	1.12	0.42	-	
		K	n/a	23	-	-	n/a	10.67	4.20	3.43	1.09	0.23	-	
		Rb	n/a	23	-	-	n/a	10.51	3.99	3.51	1.10	1.10^x, 3.73^y	-	
		Cs	n/a	23	-	-	n/a	10.41	4.07	3.45	1.07	2.99	-	
	*t*ButOH	Na	n/a	23	-	-	n/a	13.07	3.71	3.41	1.15	0.44	-	

		K	n/a	23	-	-	n/a	12.98	3.72	3.41	1.14	0.14	-	
		Rb	n/a	23	-	-	n/a	12.86	3.94	3.41	1.14	0.60[x], 2.06[y]	-	
		Cs	n/a	23	-	-	n/a	12.82	3.94	3.41	1.14	1.39	-	
	AcCN	Na	n/a	23	-	-	n/a	11.62	3.82	3.43	1.13	0.40	-	
	Ace	Na	n/a	23	-	-	n/a	11.65	3.91	3.35	1.12	0.55	-	
	HMPA (*α* Species)	Li	n/a	23	-	-	n/a	8.48	4.22	3.34	1.01	n/a	-	
		Na	n/a	23	-	-	n/a	8.48	4.20	3.33	1.01	n/a	-	
		K	n/a	23	-	-	n/a	8.49	4.21	3.33	1.00	n/a	-	7
	HMPA (*β* Species)	Li	n/a	23	-	-	n/a	10.85	4.10	3.40	1.09	<0.20	-	
		Na	n/a	23	-	-	n/a	10.90	4.10	3.40	1.09	0.44	-	
		K	n/a	23	-	-	n/a	9.80	4.10	3.40	1.09	0.25	-	
	THF	K	n/a	23	-	-	n/a	10.08	4.07	3.39	1.05	n/a	-	8
		K	n/a	23	-	-	n/a	9.23	4.15	3.38	1.01	n/a	-	
Anionic base reduction	DMSO	n/a	K*ᵗ*Bu	25	-	-	n/a	not reported						9
	DMSO	n/a	K*ᵗ*Bu	25	-	-	n/a	9.9	4.0	3.3	1.07	n/a	-	10
	THF:Hexane (75:25)	n/a	*n*-ButLi	25		-	n/a	not reported						11
	*t*ButOH:DMSO (80:20)	n/a	K*ᵗ*Bu	25		-	n/a	10.90	3.70	3.28	1.06	n/a	-	
	C_6H_6:DMSO (85:15)	n/a	*ᵗ*BuLi	20	40	1:1	2.0038	11.547	3.826	3.407	1.121	n/a	n/a	Figure S1
	THF:DMSO (75:25)	n/a	LDA	20	40	1:1	2.0040	11.414	3.874	3.419	1.123	n/a	n/a	Figure S2
	C_6H_6	n/a	LiO*ᵗ*Bu	20	40	1:5	2.0041	12.815	3.766	3.436	1.142	0.517	n/a	Figure S3
	C_6H_6:DMSO (95:5)	n/a	LiO*ᵗ*Bu	20	40	1:1	2.0041	12.666	3.812	3.446	0.980	0.496		Figure S4

C_6H_6	n/a	LiHMDS	20	40	1:5	2.0041	13.066	3.729	3.428	1.038	0.527	n/a	Figure S5
C_6H_6:DMSO (95:5)	n/a	LiHMDS	20	40	1:0.5	2.0040	11.455	3.888	3.425	1.122	n/a	n/a	Figure S6
C_6H_6	n/a	NaO^{t}Bu	20	40	1:1	2.0039	14.455	n/a	n/a	n/a	n/a	n/a	Figure S7
	n/a	NaHMDS	20	15	1:5	2.0051	11.915	3.802	3.401	1.115	1.508	n/a	Figure S11
THF	n/a	NaO^{t}Bu	20	40	1:1	2.0040	12.045	3.791	3.401	1.120	1.306	n/a	Figure S8
	n/a	NaO^{t}Bu	20	40*	1:0.5	2.0041	10.832	3.976	3.402	1.092	0.190	n/a	Figure S9
	n/a	NaHMDS	20	40	1:1	2.0040	11.909	3.802	3.397	1.116	1.463	n/a	Figure S10
	n/a	NaHMDS	20	40*	1:0.5	2.0041	10.826	3.955	3.412	1.102	0.190	n/a	Figure S12
	n/a	NaHMDS	20	40**	1:1	2.0042	10.693	3.934	3.412	1.107	0.368	n/a	Figure S13
	n/a	Na^{iPr}Cp	20	40*	1:0.25	2.0041	10.830	3.951	3.410	1.098	0.189	n/a	Figure S14
THF	n/a	KO^{t}Bu	20	40	1:1	2.0040	11.384	3.729	3.445	1.052	n/a	1	Figure S15
						2.0043	n/a	n/a	n/a	n/a	n/a	14.7	
THF:DMSO (95:5)	n/a	KO^{t}Bu	20	10	1:1	2.0039	10.660	3.911	3.362	1.086	0.196	1	Figure S16
						2.0042	n/a	n/a	n/a	n/a	n/a	2.1	
THF:DMSO (98:2)	n/a	KO^{t}Bu	20	5	1:1	2.0039	10.623	3.927	3.378	1.091	0.216	1	Figure S17
						2.0044	n/a	n/a	n/a	n/a	n/a	3.1	
THF	n/a	KO^{t}Bu	20	10**	1:1	2.0042	9.443	4.086	3.352	1.048	n/a	n/a	Figure S18
THF	n/a	KHMDS	20	40	1:1	2.0040	11.266	3.733	3.417	1.093	0.369	1	Figure S19
						2.0041	n/a	n/a	n/a	n/a	n/a	44.4	
	n/a	KHMDS	20	5	1:1	2.0039	10.624	3.926	3.376	1.090	0.233	1	Figure S20
						2.0041	n/a	n/a	n/a	n/a	n/a	4.1	

**Method**	**Solvent**	**Metal**	**Base**	T	**[1]**	**Molar ratio**	g	A_N	A_{H_p}	A_{H_o}	A_{H_m}	A_M	**Ratio**	**Ref**
		n/a	KHMDS	20	10*	1:1	2.0041	10.388	3.975	3.378	1.082	0.203	n/a	Figure S21
		n/a	KHMDS	20	10**	1:1	2.0041	9.430	4.074	3.340	1.042	n/a	n/a	Figure S22

x = Splitting due to ^{85}Rb, y = Splitting due to ^{87}Rb. * and ** indicate that the presence of a chelating agent – 15-crown ether and [2.2.2]-cryptand, respectively. Added in a 1:1 molar ratio to nitrobenzene.

Section S2: Single crystal XRD data.

Inside a glovebox, nitrobenzene (15 mg, 0.12 mmol) was mixed with a stoichiometric amount of L_1 (45.5 mg, 0.12 mmol) in 3.5 mL of THF; 0.5 mL of potassium bis(trimethylsilyl)amide (17 mg, 0.12 mmol) in THF were then carefully added to the mixture. The solution was kept at room temperature for one hour to ensure the completion of the reaction. The resulting deep-coloured solution was carefully covered with a layer of dry hexane (2.0 mL) and kept for 7 days at -35 $^{\circ}$C in the freezer. Relatively large crystals formed on the walls at the intersection between the two solvents.

A suitable crystal of $[\mathbf{1}^{\bullet-}]$:$[K(L_1)]^+$ was selected and mounted on MiTeGen polymer loop using FOMBLING oil. Single-Crystall X-ray diffracton experiments were performed on a Bruker D8 Venture diffractometer equipped with Kα radiation (0.71073 Å) at 150 K. The data reduction was performed with the APEX4 software and the absorption correction was done with SADABS. The estructure was solve and refined using SHELXT[12] and SHELXL[12] packages implemented in the Olex2[13] software. All non-H atoms were refined anisotropically, and H atoms were placed in calculated positions and refined with idealized geometries.

Table S2. Crystal data and structure refinement for $[\mathbf{1}^{\bullet-}]$:$[K(L_1)]^+$

Sample	$[\mathbf{1}^{\bullet-}]$:$[K(L_1)]^+$
Empirical formula	$C_{24}H_{41}KN_3O_8$
Formula weight	538.70
Temperature/K	150.00
Crystal system	monoclinic
Space group	*P*2/*n*
a/Å	15.7863(4)
b/Å	10.1204(2)
c/Å	17.4697(4)
α/°	90
β/°	98.0340(10)
γ/°	90
Volume/Å^3	2763.63(11)
Z	4
ρ_{calc}g/cm^3	1.295
μ/mm^{-1}	0.242
F(000)	1156.0
Crystal size/mm^3	0.1 × 0.08 × 0.05
Radiation	MoKα (λ = 0.71073)
2Θ range for data collection/°	4.664 to 52.766
Index ranges	$-19 \leq h \leq 19$<br>$-12 \leq k \leq 12$<br>$-21 \leq l \leq 21$
Reflections collected	41600
Independent reflections	5654 [R_{int} = 0.0332, R_{sigma} = 0.0172]
Data/restraints/parameters	5654/0/325
Goodness-of-fit on F^2	1.059
Final R indexes [I>=2σ (I)]	R_1 = 0.0331, wR_2 = 0.0883
Final R indexes [all data]	R_1 = 0.0389, wR_2 = 0.0954
Largest diff. peak/hole / e Å^{-3}	0.40/-0.32

Section S3: DFT calculations & reaction pathways.

All the reaction schemes investigated are presented below. A summary of the calculated reaction energies ΔG are given in Table S3.

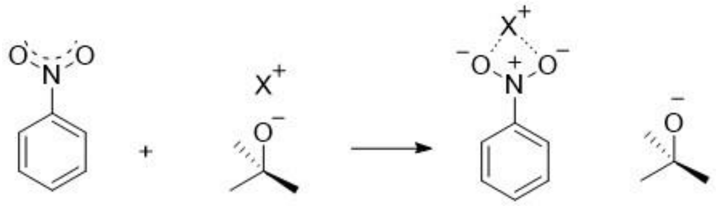

Scheme S1. Nitrobenzene cation formation by transfer of metal ion from tert-butoxide salts (X=Li, Na, K).

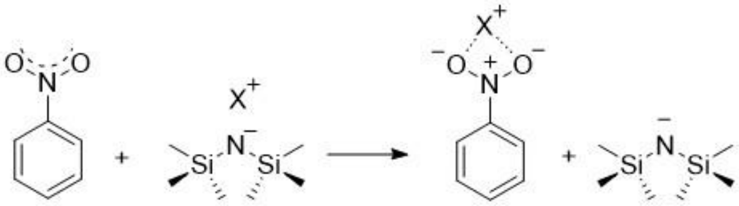

Scheme S2. Nitrobenzene cation formation by transfer of metal ion from bis(trimethylsilyl)amide salts (X=Li, Na, K).

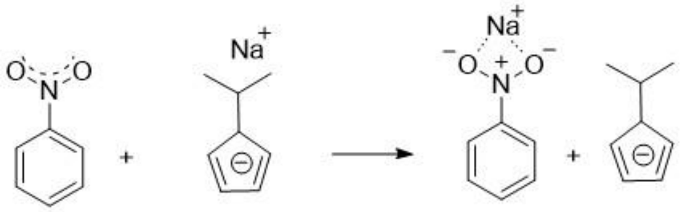

Scheme S3. Nitrobenzene cation formation by transfer of metal ion from sodium isopropylcyclopentadienide.

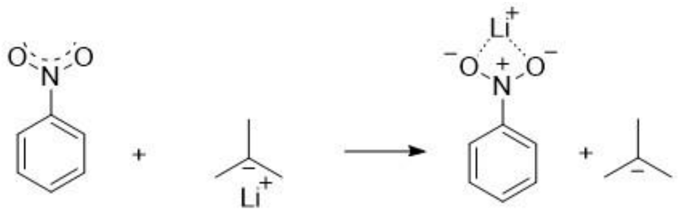

Scheme S4. Nitrobenzene cation formation by transfer of Lithium ion from tert-butyllithium.

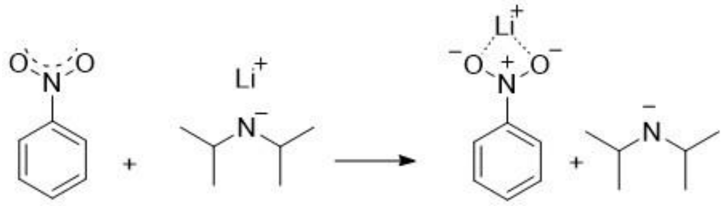

Scheme S5. Nitrobenzene cation formation by transfer of Lithium ion from Lithium diisopropylamide.

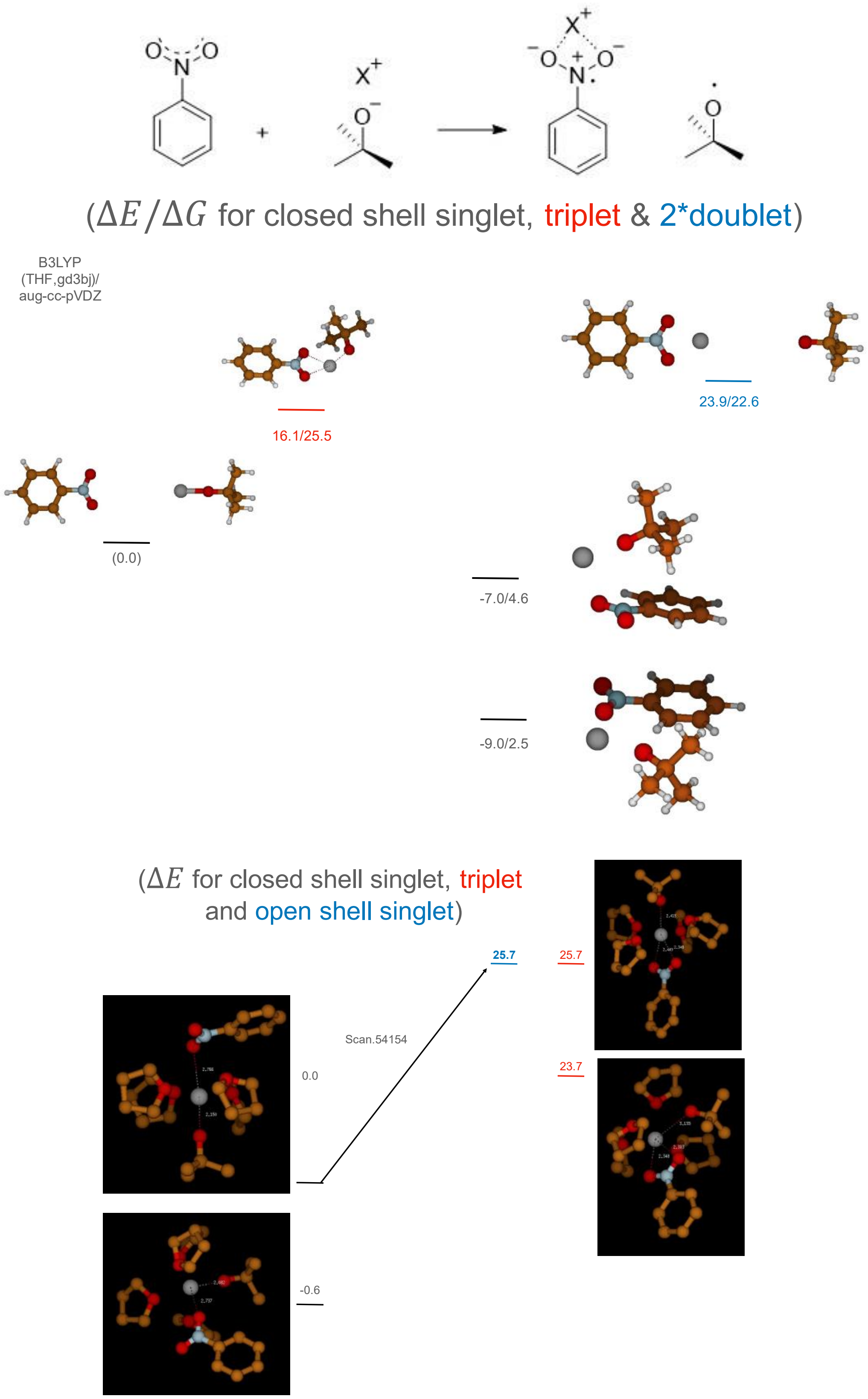

Scheme S6. Top: Nitrobenzene radical formation by tert-butoxide salts (X=Li, Na, K). Middle: DFT-calculated reaction profile for the formation of $[\mathbf{1}^{\bullet-}][\mathbf{Na}^{+}]$ with a PCM solvation model for THF. Values in kcal/mol. Bottom: DFT-calculated reaction profile for the formation of $[\mathbf{1}^{\bullet-}][\mathbf{Na}^{+}]$ with explicity consideration of THF solvation. ΔE and ΔG values are given in kcal/mol.

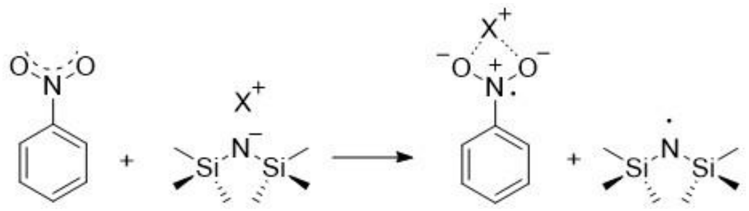

Scheme S7. Nitrobenzene radical formation by bis(trimethylsilyl)amide salts (X=Li, Na, K).

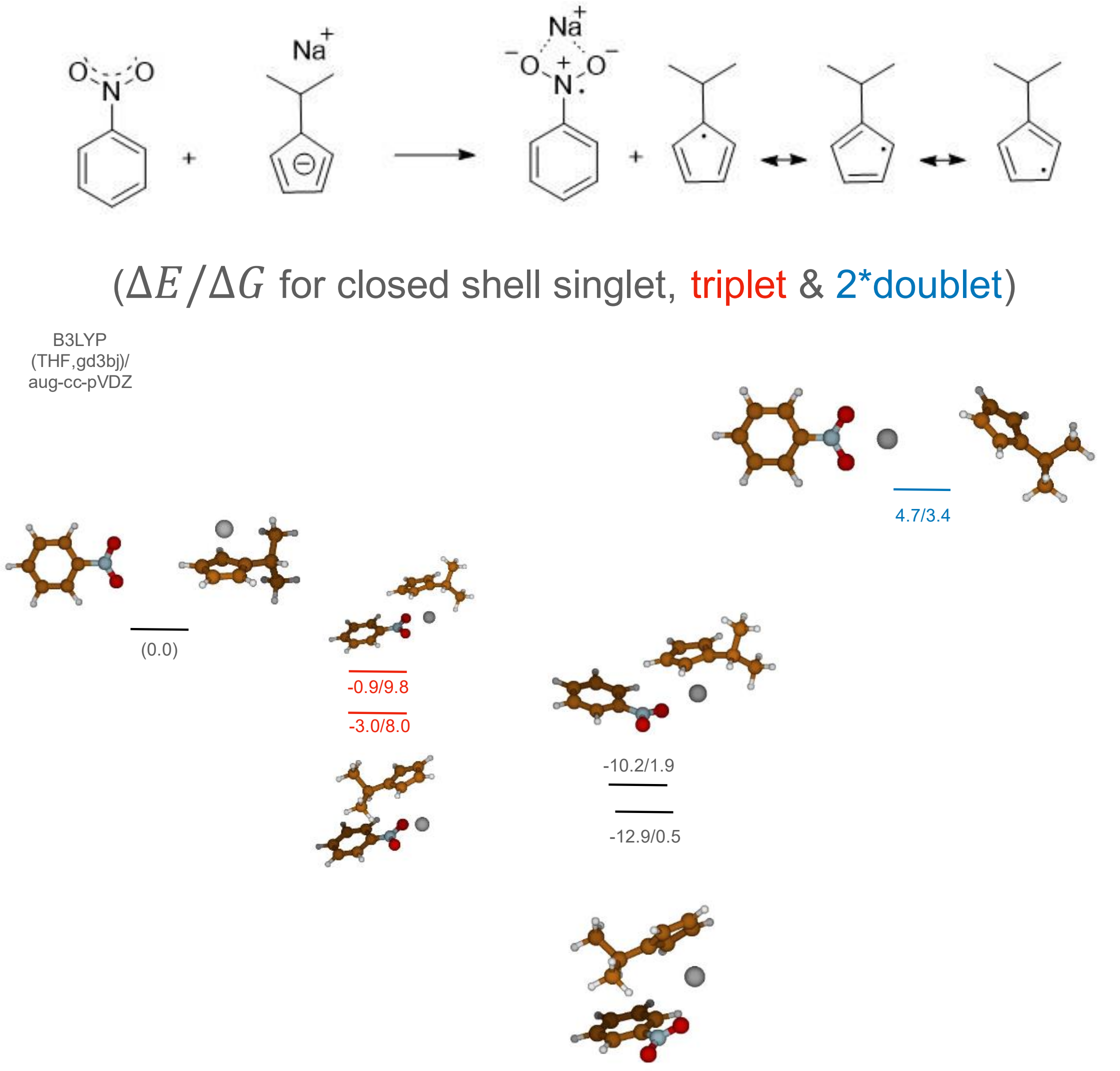

Scheme S8. Top: Nitrobenzene radical formation by sodium isopropylcyclopentadienide. Bottom: DFT-calculated reaction profile for the formation of [$1^{\bullet-}$][Na^+] with a PCM solvation model for THF. Values in kcal/mol.

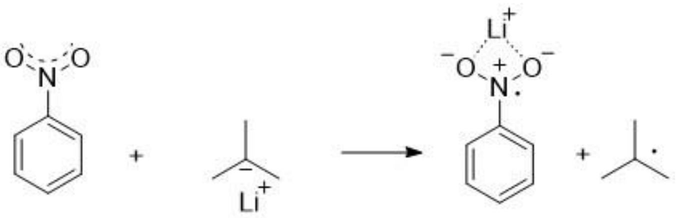

Scheme S9. Nitrobenzene radical formation by tert-butyllithium.

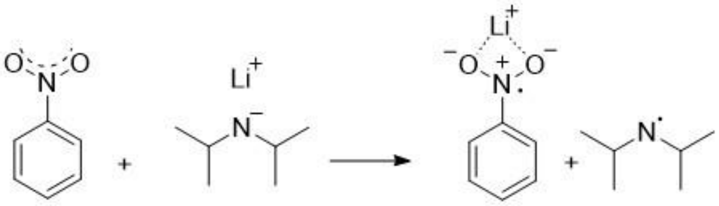

Scheme S10. Nitrobenzene radical formation by Lithium diisopropylamide.

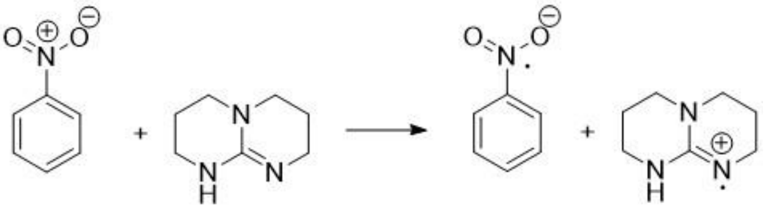

Scheme S11. Nitrobenzene radical formation by triazabicyclodecene.

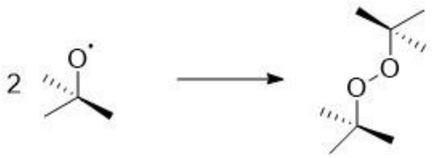

Scheme S12. Dimerization of tert-butoxide radical to form di-tert-butyl peroxide.

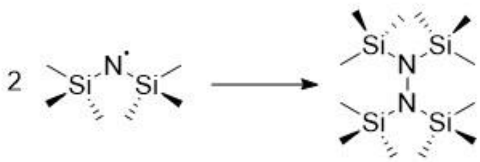

Scheme S13. Dimerization of bis(trimethylsilyl)amide radical to form tetrakis (trimethylsilyl)hydrazine.

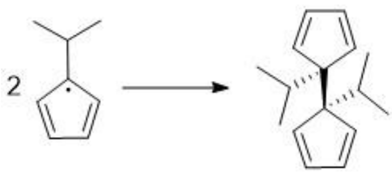

Scheme S14. Dimerization of isopropylcyclopentadienide radical.

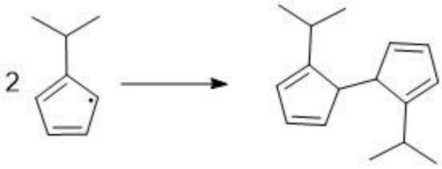

Scheme S15. Dimerization of isopropylcyclopentadienide radical.

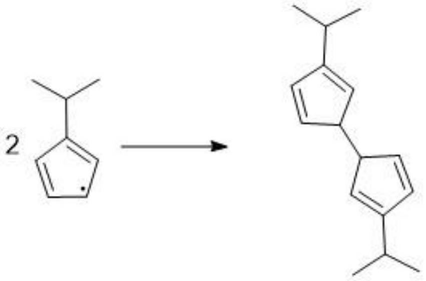

Scheme S16. Dimerization of isopropylcyclopentadienide radical.

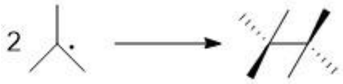

Scheme S17. Dimerization of tert-butyl radical.

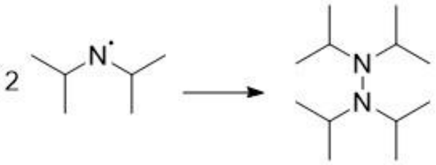

Scheme S18. Dimerization of diisopropylamide radical.

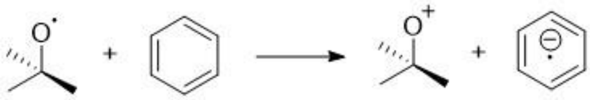

Scheme S19. Electron transfer from tert-butoxide radical to benzene.

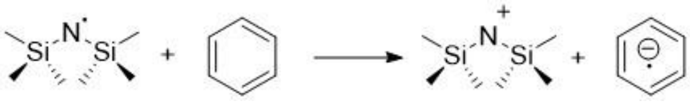

Scheme S20. Electron transfer from bis(trimethylsilyl)amide radical to benzene.

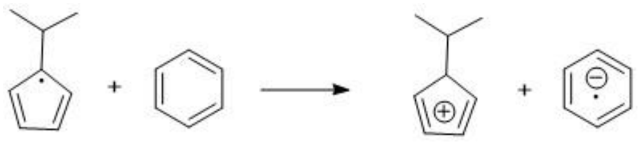

Scheme S21. Electron transfer from isopropylcyclopentadienide radical to benzene.

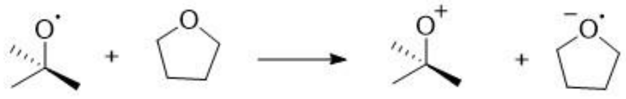

Scheme S22. Electron transfer from tert-butoxide radical to THF.

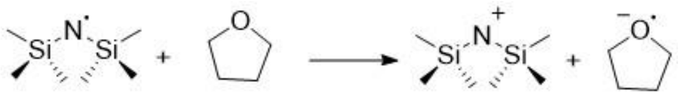

Scheme S23. Electron transfer from bis(trimethylsilyl)amide radical to THF.

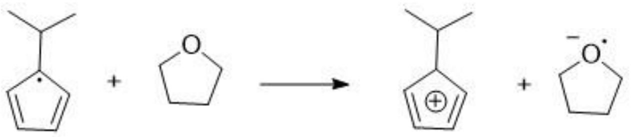

Scheme S24. Electron transfer from isopropylcyclopentadienide radical to THF.

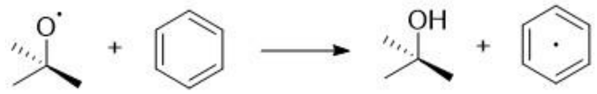

Scheme S25. Hydrogen atom transfer (HAT) from benzene to tert-butoxide radical.

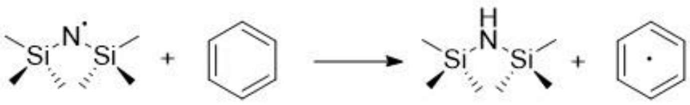

Scheme S26. HAT from benzene to bis(trimethylsilyl)amide radical.

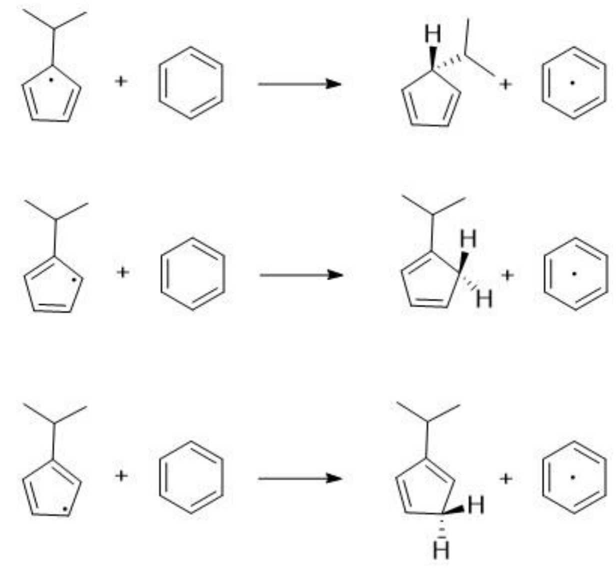

Scheme S27. HAT from benzene to isopropylcyclopentadienide radical.

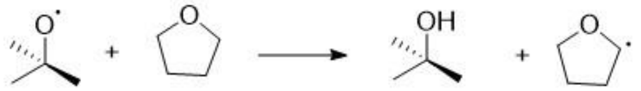

Scheme S28. HAT from position 1 of THF to tert-butoxide radical.

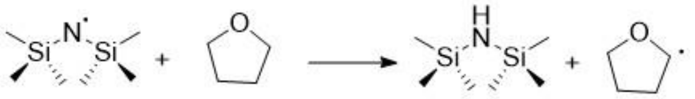

Scheme S29. HAT from position 1 of THF to bis(trimethylsilyl)amide radical.

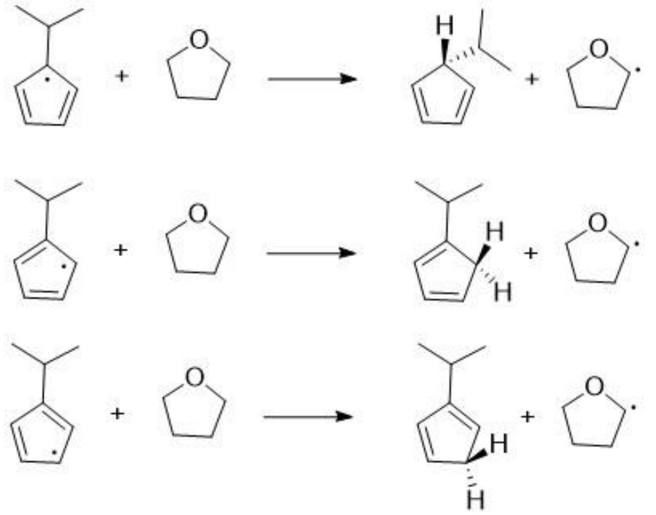

Scheme S30. HAT from position 1 of THF to isopropylcyclopentadienide radical.

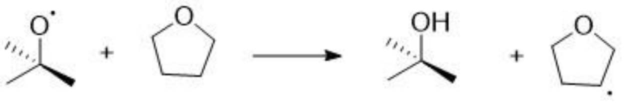

Scheme S31. HAT from position 2 of THF to tert-butoxide radical.

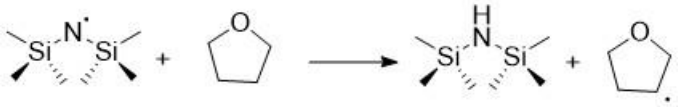

Scheme S32. HAT from position 2 of THF to bis(trimethylsilyl)amide radical.

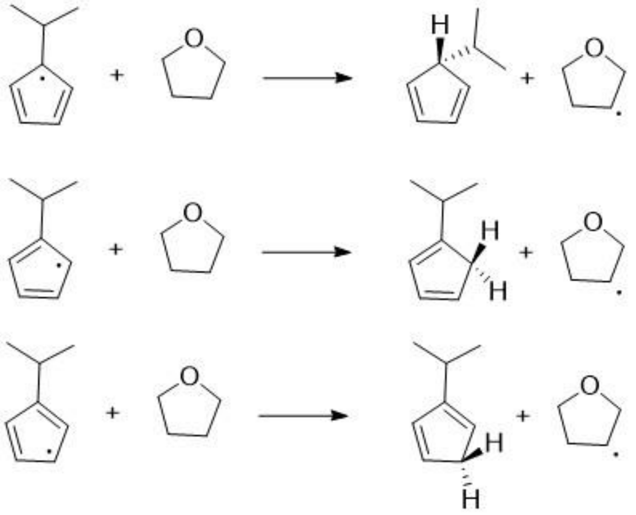

Scheme S33. HAT from position 2 of THF to isopropylcyclopentadienide radical.

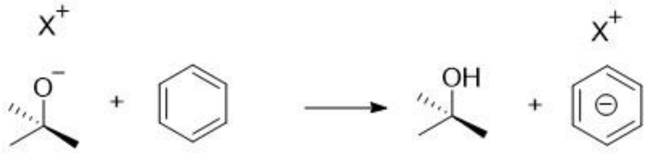

Scheme S34. Proton transfer from benzene to Li/Na/K (X) salt of tert-butoxide to form Li/Na/K (X) salt of benzene anion and tert-butanol.

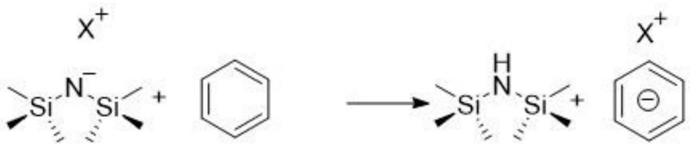

Scheme S35. Proton transfer from benzene to Li/Na/K (X) salt of bis(trimethylsilyl)amide to form Li/Na/K (X) salt of benzene anion and bis(trimethylsilyl)amine.

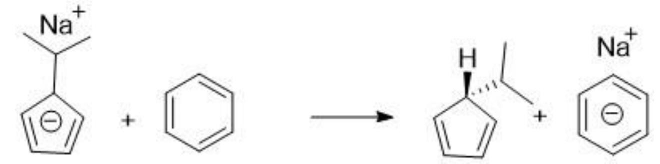

Scheme S36. Proton transfer from benzene to sodium isopropylcyclopentadienide to form sodium salt of benzene anion and 5-isopropylcyclopenta-1,3-diene.

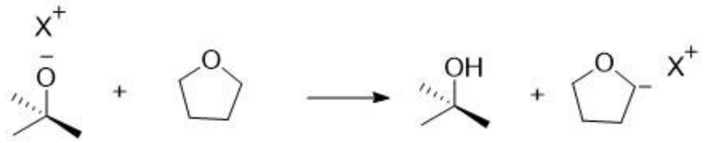

Scheme S37. Proton transfer from position 1 of THF to Li/Na/K (X) salt of tert-butoxide to form Li/Na/K (X) salt of THF-1 anion and tert-butanol.

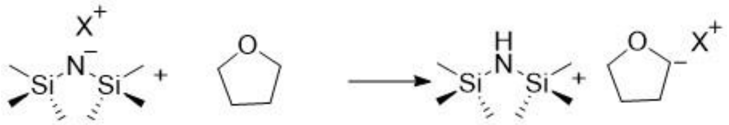

Scheme S38. Proton transfer from position 1 of THF to Li/Na/K (X) salt of bis(trimethylsilyl)amide to form Li/Na/K (X) salt of THF-1 anion and bis(trimethylsilyl)amine.

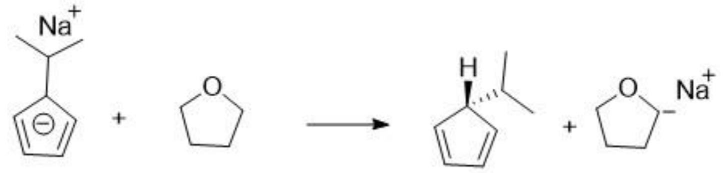

Scheme S39. Proton transfer from position 1 of THF to sodium isopropylcyclopentadienide to form sodium salt of THF-1 anion and 5-isopropylcyclopenta-1,3-diene.

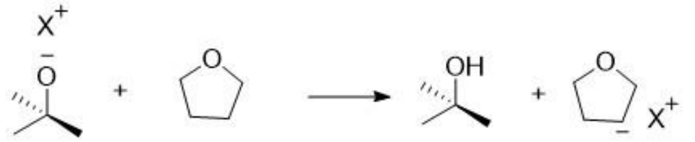

Scheme S40. Proton transfer from position 2 of THF to Li/Na/K (X) salt of tert-butoxide to form Li/Na/K (X) salt of THF-2 anion and tert-butanol.

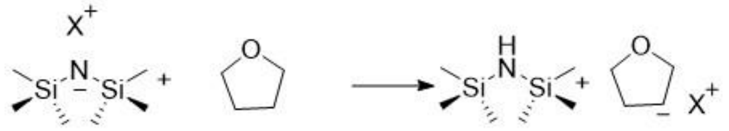

Scheme S41. Proton transfer from position 2 of THF to Li/Na/K (X) salt of bis(trimethylsilyl)amide to form Li/Na/K (X) salt of THF-2 anion and bis(trimethylsilyl)amine.

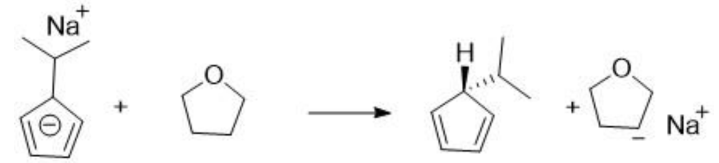

Scheme S42. Proton transfer from position 2 of THF to sodium isopropylcyclopentadienide to form sodium salt of THF-2 anion and 5-isopropylcyclopenta-1,3-diene.

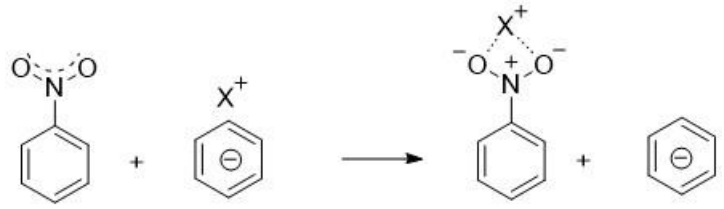

Scheme S43. Nitrobenzene cation formation by transfer of metal ion from Li/Na/K (X) salts of benzene anion.

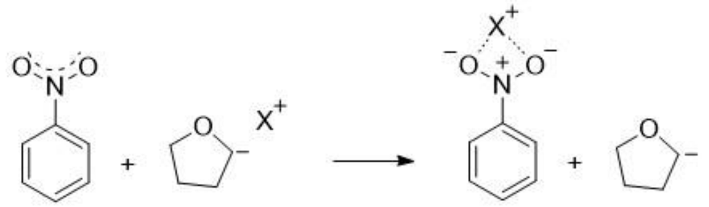

Scheme S44. Nitrobenzene cation formation by transfer of metal ion from Li/Na/K (X) salts of THF-1 anion.

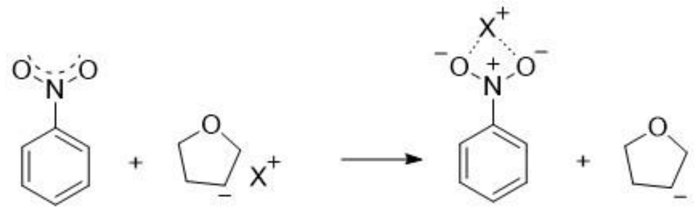

Scheme S45. Nitrobenzene cation formation by transfer of metal ion from Li/Na/K (X) salts of THF-2 anion.

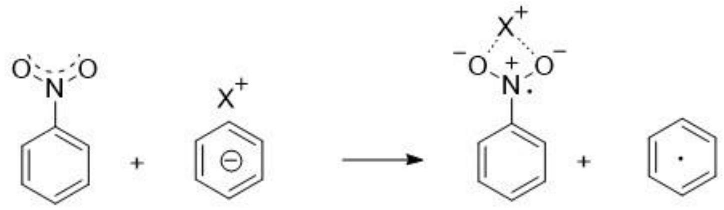

Scheme S46. Nitrobenzene radical formation by Li/Na/K (X) salts of benzene anion.

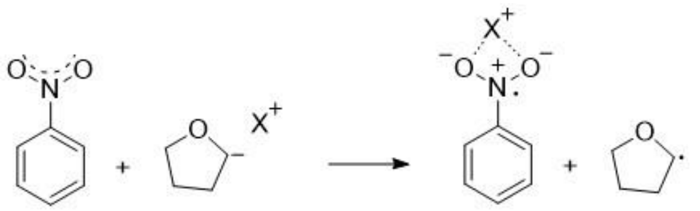

Scheme S47. Nitrobenzene radical formation by Li/Na/K (X) salts of THF-1 anion.

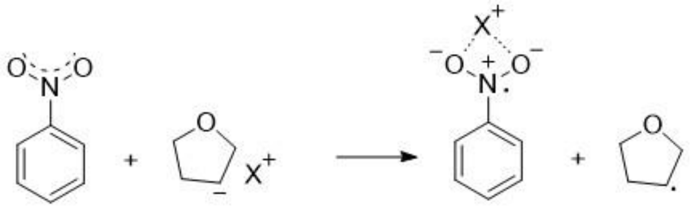

Scheme S48. Nitrobenzene radical formation by Li/Na/K (X) salts of THF-2 anion.

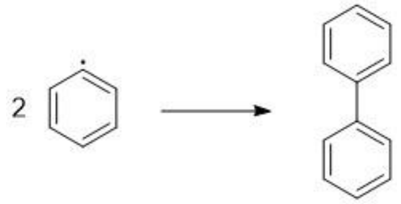

Scheme S49. Dimerization of benzene radical.

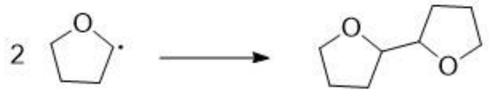

Scheme S50. Dimerization of THF radical at position 1.

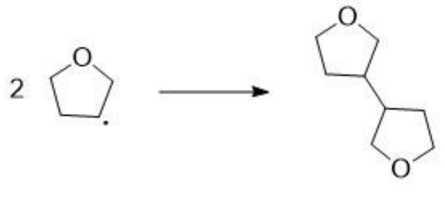

Scheme S51. Dimerization of THF radical at position 2.

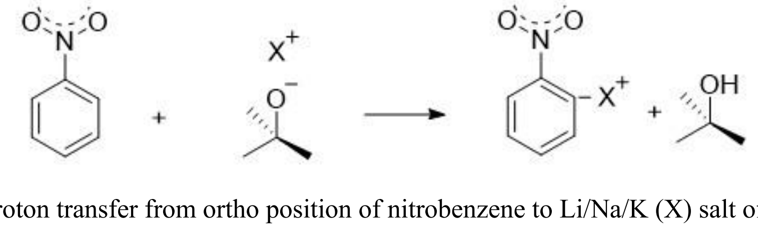

Scheme S52. Proton transfer from ortho position of nitrobenzene to Li/Na/K (X) salt of tert-butoxide to form Li/Na/K (X) salt of nitrobenzene ortho anion and tert-butanol.

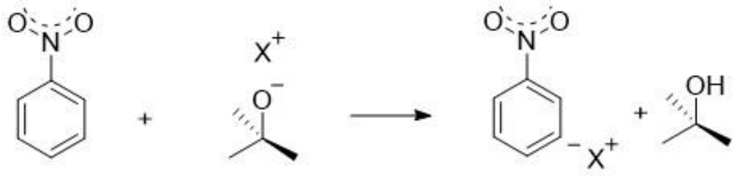

Scheme S53. Proton transfer from meta position of nitrobenzene to Li/Na/K (X) salt of tert-butoxide to form Li/Na/K (X) salt of nitrobenzene meta anion and tert-butanol.

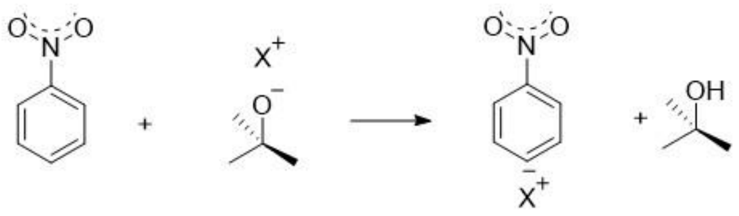

Scheme S54. Proton transfer from para position of nitrobenzene to Li/Na/K (X) salt of tert-butoxide to form Li/Na/K (X) salt of nitrobenzene para anion and tert-butanol.

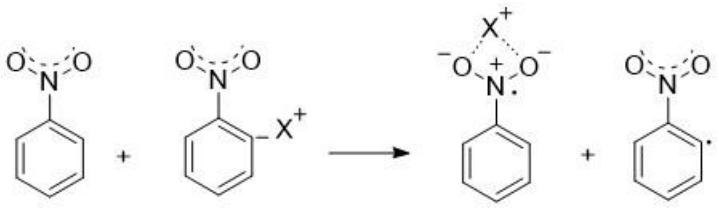

Scheme S55. Nitrobenzene radical formation by Li/Na/K (X) salts of nitrobenzene ortho anion.

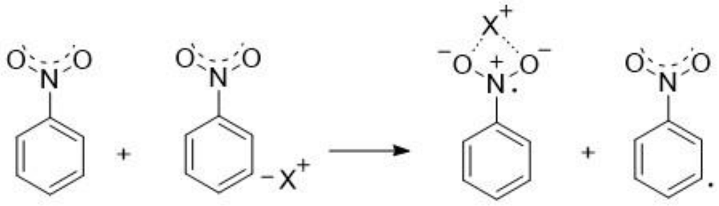

Scheme S56. Nitrobenzene radical formation by Li/Na/K (X) salts of nitrobenzene meta anion.

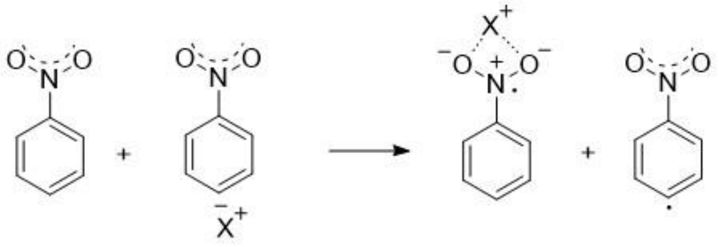

Scheme S57. Nitrobenzene radical formation by Li/Na/K (X) salts of nitrobenzene para anion.

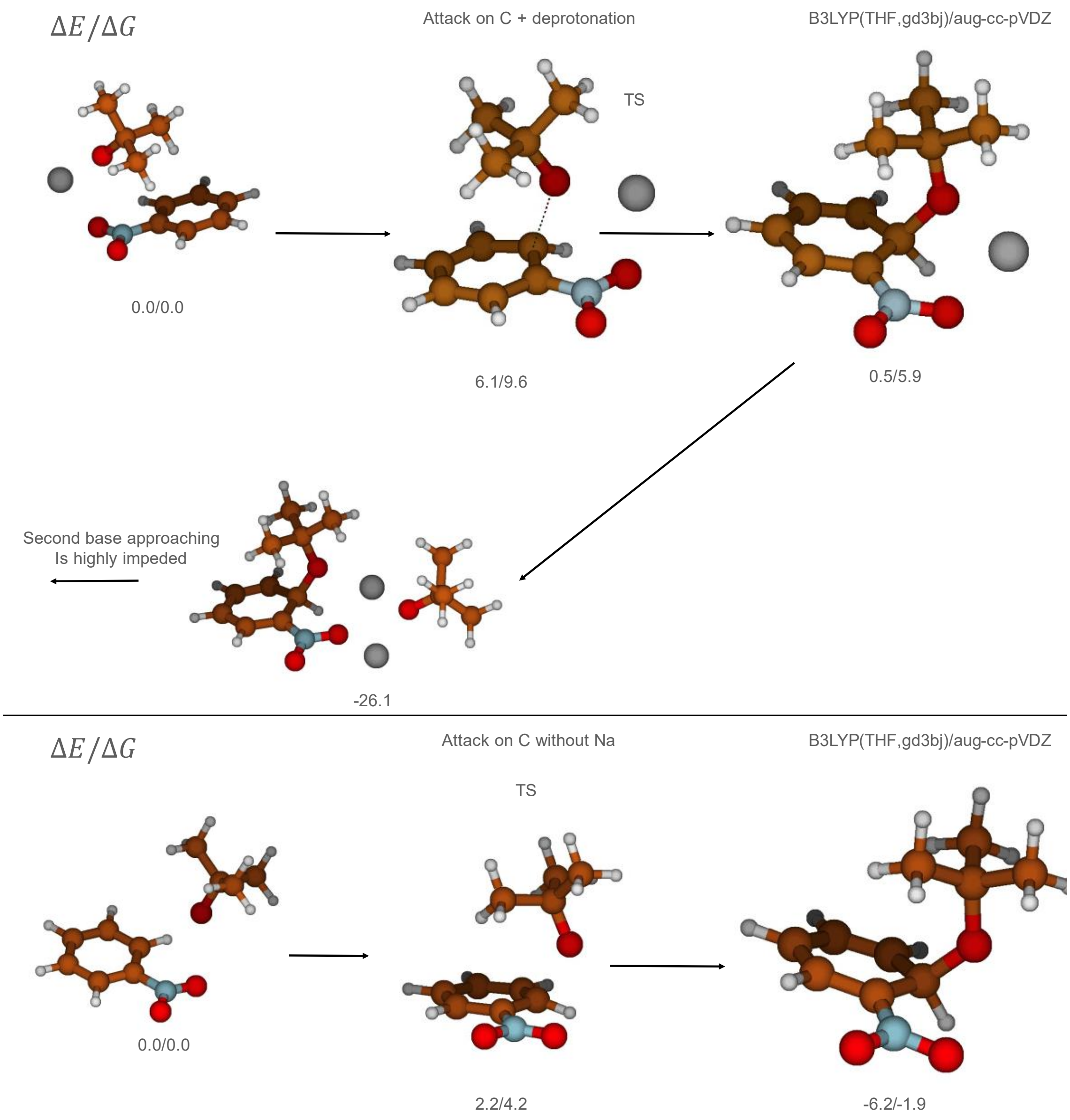

Scheme S58. DFT-calculated reaction profile of Guthrie's proposed [**1**$^{\bullet-}$][*Na*$^{+}$] formation with an implicit PCM model for THF. ΔE and ΔG values are given in kcal/mol. Top: Considering Na^{+} ion. Bottom: Na^{+} ion not considered.

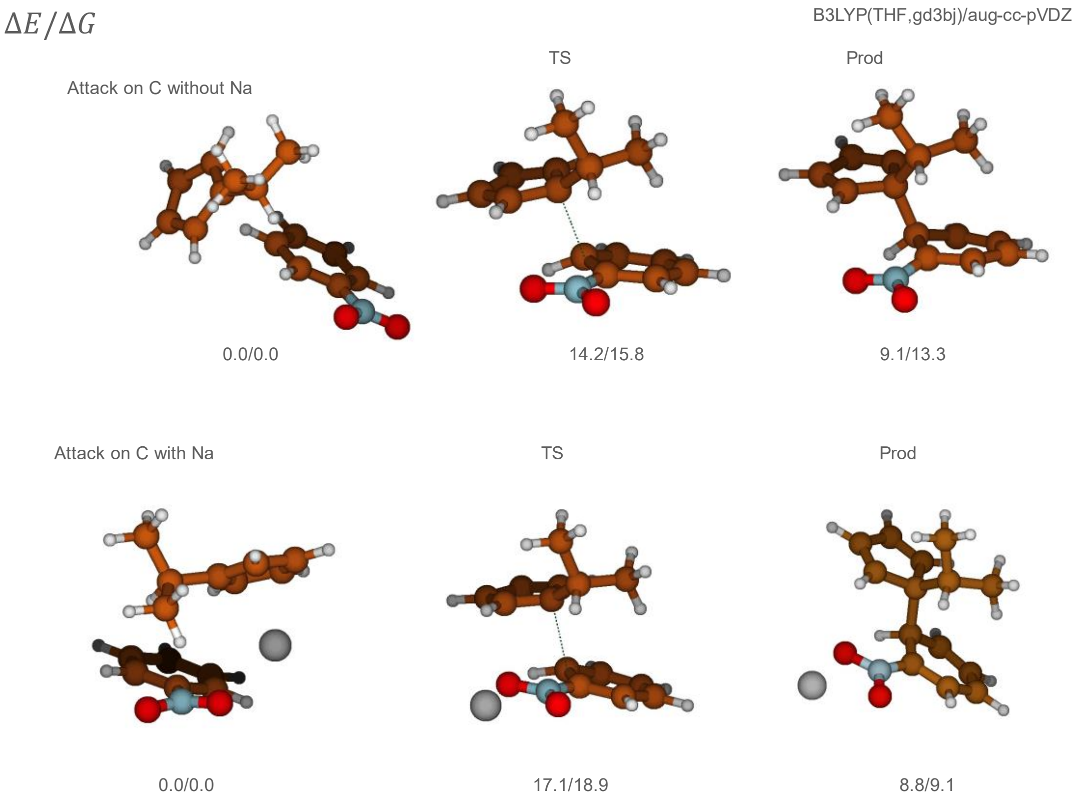

Scheme S59. DFT-calculated reaction profile of Guthrie's proposed [**1**$^{\bullet-}$][**Na**$^{+}$] formation with $^{i\mathrm{Pr}}\mathrm{Cp}^{-}$ anion and an implicit PCM model for THF. ΔE and ΔG values are given in kcal/mol.

The considered reaction profiles can be grouped by the following types:

- Scheme S1–Scheme S5 describe $[\mathbf{1}^{\bullet -}][\mathbf{X}^{+}]$ formation by a non-bonded single electron transfer mechanism from the oxidised base, in the following order: XO^{t}Bu (X=Li, Na, K), XHMDS (X=Li, Na, K), Na^{iPr}Cp, tButLi and LDA.

- Scheme S6–Scheme S11 describe $[\mathbf{1}^{\bullet -}][\mathbf{X}^{+}]$ formation by a bonded single electron transfer mechanism from the oxidised base, in the following order: XO^{t}Bu (X=Li, Na, K), XHMDS (X=Li, Na, K), Na^{iPr}Cp, tButLi, LDA and TBD.

- Scheme S12–Scheme S18 describe the dimerization of the oxidised base, in the following order: O^{t}Bu, HMDS, ^{iPr}Cp (through position 1), ^{iPr}Cp (through position 2), ^{iPr}Cp (through position 3), tBut and LDA.

- Scheme S19–Scheme S24 describe the single electron transfer from the oxidised base to a solvent molecule, in the following order O^{t}Bu, HMDS and ^{iPr}Cp to solvents benzene (first three schemes) and THF (next three schemes).

- Scheme S25–Scheme S33 describe the hydrogen atom transfer (HAT) from solvent molecule to the oxidised base, in the following order: benzene to O^{t}Bu, HMDS and ^{iPr}Cp (Scheme S25-Scheme S27), THF (from position 1) to O^{t}Bu, HMDS and ^{iPr}Cp (S28-S30); THF (from position 2) to O^{t}Bu, HMDS and ^{iPr}Cp (Scheme S31-Scheme S33). For ^{iPr}Cp energies for HAT to three different non-equivalent positions of the oxidised base are provided.

- Scheme S34–Scheme S42 describe the deprotonation of a solvent molecule by the base, in the following order XO^{t}Bu (X=Li, Na, K), XHMDS (X=Li, Na, K) and Na^{iPr}Cp forming corresponding salts of benzene anion (Scheme S34-Scheme S36), THF-1 anion (Scheme S37-Scheme S39) and THF-2 anion (Scheme S40-Scheme S42).

- Scheme S43–Scheme S45 describe $[\mathbf{1}^{\bullet -}\mathbf{X}^{+}]$ formation by a non-bonded single electron transfer mechanism from the deprotonated anion solvent, in the following order Li/Na/K salts of benzene anion, THF-1 anion and THF-2 anion.

- Scheme S46–Scheme S48 describe $[\mathbf{1}^{\bullet -}\mathbf{X}^{+}]$ formation by a bonded single electron transfer mechanism from the deprotonated anion solvent, in the following order Li/Na/K salts of benzene anion, THF-1 anion and THF-2 anion.

- Scheme S49–Scheme S51 describe the dimerization of the radical-containing solvent molecule, in the following order Benzene radical, THF-1 radical and THF-2 radical.

- Scheme S52–Scheme S54 describe the deprotonation of **[1]** by the XO^{t}Bu (X=Li, Na, K) in ortho, meta and para positions.

- Scheme S55–Scheme S57 describe the reduction of **[1]** by **[1]**$^{-}$ in ortho, meta and para positions.

- Scheme S58–Scheme S59 $[\mathbf{1}^{\bullet -}]([\mathbf{Na}^{+}])$ formation following Guthrie's mechanism.

Table S3. Energy reactions ΔG (kcal/mol) calculated for the different scheme numbers (#). For a given scheme number, three or two values refer to Li, Na, and K or Na and K cations, respectively, except for # 27, 30 and 33, which represent the three possible positions for HAT. The different types of reactions are highlighted in alternating grey and white backgrounds.

#	ΔG	#	ΔG	#	ΔG	#	ΔG	#	ΔG	#	ΔG
Scheme ***S1***	303.4<br>107.6<br>98.0	Scheme ***S11***	130.0	Scheme ***S21***	189.7	Scheme ***S31***	-6.6	Scheme ***S41***	30.3<br>28.1<br>31.9	Scheme ***S51***	-65.1
Scheme ***S2***	290.6<br>93.3<br>83.6	Scheme ***S12***	-19.5	Scheme ***S22***	147.8	Scheme ***S32***	-9.7	Scheme ***S42***	47.3	Scheme **S52**	11.0<br>7.9<br>10.9
Scheme ***S3***	97.8	Scheme ***S13***	-44.4	Scheme ***S23***	142.4	Scheme ***S33***	20.3<br>16.9<br>16.7	Scheme ***S43***	111.5<br>104.5<br>90.7	Scheme **S53**	33.7<br>23.3<br>25.8
Scheme ***S4***	108.6	Scheme ***S14***	-11.0	Scheme ***S24***	189.8	Scheme ***S34***	39.1<br>29.0<br>33.2	Scheme ***S44***	123.6<br>111.3<br>99.1	Scheme **S54**	34.2<br>23.7<br>26.4
Scheme ***S5***	106.4	Scheme ***S15***	-27.9	Scheme ***S25***	8.9	Scheme ***S35***	28.7<br>24.7<br>28.8	Scheme ***S45***	116.4<br>107.7<br>94.2	Scheme **S55**	24.2<br>23.4<br>21.2
Scheme ***S6***	23.4<br>19.5<br>20.3	Scheme ***S16***	-29.7	Scheme ***S26***	5.8	Scheme ***S36***	43.9	Scheme ***S46***	-6.7<br>-0.6<br>-4.0	Scheme **S56**	-0.4<br>6.1<br>4.3
Scheme S*7*	16.2<br>18.4<br>19.0	Scheme ***S17***	-50.7	Scheme ***S27***	35.8<br>32.4<br>32.1	Scheme ***S37***	34.6<br>29.8<br>32.4	Scheme ***S47***	-21.7<br>-20.9<br>-22.7	Scheme **S57**	-1.0<br>5.5<br>3.6
Scheme ***S8***	7.5	Scheme ***S18***	-17.8	Scheme ***S28***	-10.6	Scheme ***S38***	24.2<br>25.4<br>28.0	Scheme ***S48***	-23.9<br>-19.5<br>-22.6		
Scheme ***S9***	-36.4	Scheme ***S19***	147.7	Scheme ***S29***	-13.7	Scheme ***S39***	44.7	Scheme ***S49***	-99.6		
Scheme ***S10***	-9.2	Scheme ***S20***	142.3	Scheme ***S30***	16.3<br>12.9<br>12.7	Scheme ***S40***	40.7<br>32.8<br>36.3	Scheme ***S50***	-61.4		

Table S4. Model spin Hamiltonian parameters calculated for [**1**$^{•-}$]([**X**$^+$]).

		[**1**$^{•-}$]([**Li**$^+$])	[**1**$^{•-}$]([**Na**$^+$])	[**1**$^{•-}$]([**K**$^+$])
$\boldsymbol{g}$	$\boldsymbol{g_{xx}, g_{yy}, g_{zz}}$	2.002, 2.005, 2.006	2.002, 2.005, 2.006	2.002, 2.006, 2.006
	$\boldsymbol{g_{iso}}$	2.004	2.005	2.005
$\boldsymbol{A_N}$	$\boldsymbol{A_{xx}, A_{yy}, A_{zz}}$	-0.208, -0.212, 27.429	-0.474, -0.490, 25.925	-0.605, -0.626, 24.861
	$\boldsymbol{A_{iso}}$	9.012	8.325	7.881
$\boldsymbol{A_M}$	$\boldsymbol{A_{xx}, A_{yy}, A_{zz}}$	-0.166, -1.513, -1.691	-1.070, -1.382, -2.190	-0.238, -0.374, -0.473
	$\boldsymbol{A_{iso}}$	-1.124	-1.547	-0.362
$\boldsymbol{A_o}$	$\boldsymbol{A_{xx}, A_{yy}, A_{zz}}$	-2.271, -4.385, -4.429	-2.230, -4.123, -4.259	-2.251, -4.082, -4.228
	$\boldsymbol{A_{iso}}$	-3.696	-3.537	-3.521

A_m	A_{xx}, A_{yy}, A_{zz}	0.882, 1.205, 2.384	0.791, 1.129, 2.256	0.787, 1.127, 2.249
	A_{iso}	1.491	1.392	1.388
A_p	A_{xx}, A_{yy}, A_{zz}	-1.539, -3.925, -6.454	-1.511, -3.816, -6.312	-1.542, -3.832, -6.360
	A_{iso}	-3.975	-3.880	-3.912

Table S5. Model spin Hamiltonian parameters calculated for $[\mathbf{1}^{\bullet-}]([\mathbf{X}^+])$ with a PCM solvation model to describe THF.

		$[\mathbf{1}^{\bullet-}]([\mathbf{Li}^+])$	$[\mathbf{1}^{\bullet-}]([\mathbf{Na}^+])$	$[\mathbf{1}^{\bullet-}]([\mathbf{K}^+])$
g	g_{xx}, g_{yy}, g_{zz}	2.002, 2.006, 2.004	2.002, 2.006, 2.005	2.002, 2.006, 2.006
	g_{iso}	2.004	2.005	2.005
A_N	A_{xx}, A_{yy}, A_{zz}	-0.253, -0.255, 27.598	-0.508, -0.518, 26.053	-0.657, -0.673, 24.880
	A_{iso}	9.030	8.342	7.854
A_X	A_{xx}, A_{yy}, A_{zz}	-0.011, -1.368, -1.632	-0.976, -1.057, -1.841	-0.198, -0.306, -0.400
	A_{iso}	-1.004	-1.291	-0.301
A_o	A_{xx}, A_{yy}, A_{zz}	-2.230, -4.262, -4.346	-2.213, -4.045, -4.205	-2.215, -3.978, -4.146
	A_{iso}	-3.613	-3.487	-3.447
A_m	A_{xx}, A_{yy}, A_{zz}	0.810, 1.155, 2.277	0.753, 1.102, 2.190	0.724, 1.081, 2.152
	A_{iso}	1.414	1.348	1.319
A_p	A_{xx}, A_{yy}, A_{zz}	-1.519, -3.878, -6.352	-1.505, -3.789, -6.230	-1.525, -3.792, -6.252
	A_{iso}	-3.917	-3.841	-3.857

Table S6. Energy values of reactants and products for $[\mathbf{1}^{\bullet-}][\mathbf{Na}^+]$ formation by NaO^{t}Bu (Scheme S6) calculated with different functionals.

Functional	Reactant free energy (in Hartree)		Product free energy (in Hartree)		ΔG (kcal/mol)
	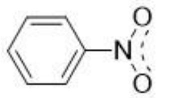	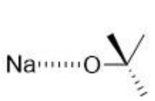	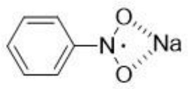	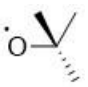	
B3LYP	-436.751520	-395.344292	-599.112513	-232.952216	19.5
CAM- B3LYP	-436.539751	-395.190618	-598.881704	-232.820192	17.9
LC-WPBE	-436.436782	-395.106894	-598.719567	-232.795780	17.8
WB97XD	-436.590517	-395.240503	-598.930678	-232.866992	20.9
LC-WPBE Dispersion	-436.443105	-395.115405	-598.727038	-232.802335	18.3
LC-WHPBE Dispersion	-436.441923	-395.114177	-598.725376	-232.801667	18.2
WB97XD Dispersion	-436.590889	-395.239378	-598.930400	-232.866490	20.9

Section S4: Uv-Vis absorption spectra.

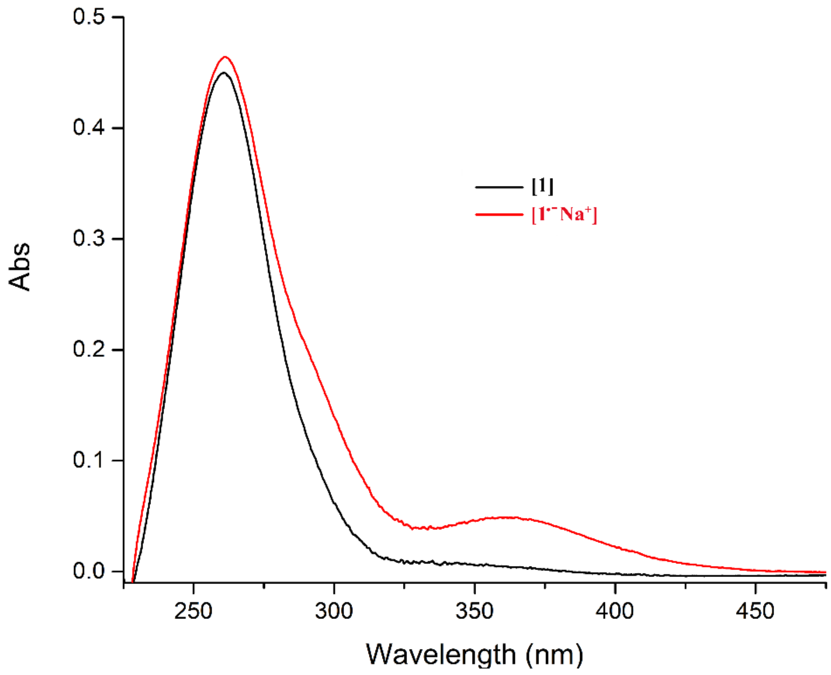

Figure S32. Absorption spectra of **[1]** and **[1•-][Na+]** in THF.

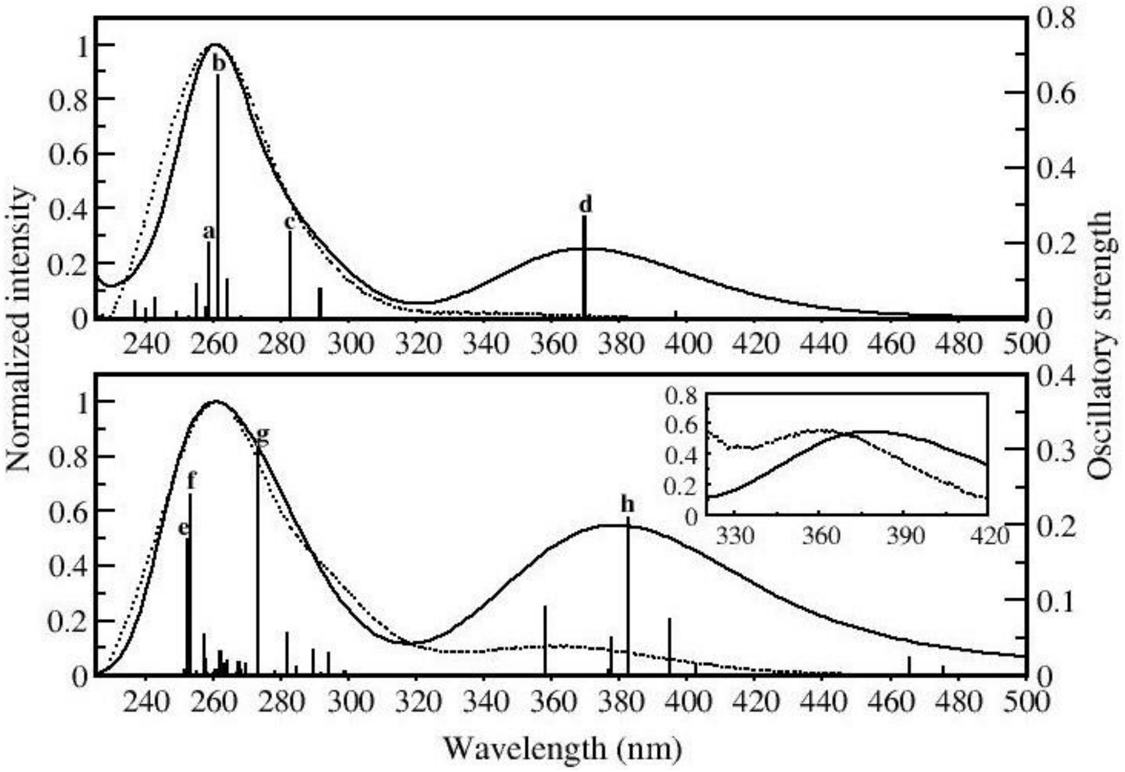

Figure S33. Experimental (dashed line) and theoretical (solid line) absorption spectra of **[1]** (above) and **[1•-Na+]** (below). The intensities are normalized to highest value of one. The theoretical curves are horizontally shifted by 76.2 nm and 86.2 nm respectively for singlet and doublet spectrums. In the inset of the figure below the experimental curve increased by 5.25 time and the theoretical curve near the second maxima are shown. The figures also show theoretical oscillatory strength values as vertical lines. Some

these lines which show prominent values are marked and natural transition orbital calculations were performed for these states (results shown below).

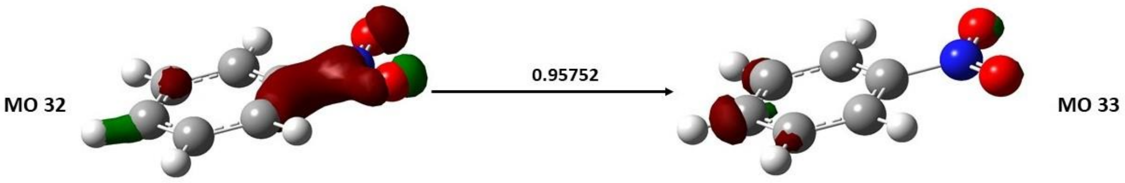

Figure S34. Natural transition orbitals with coefficient value for state **a** in Figure S33.

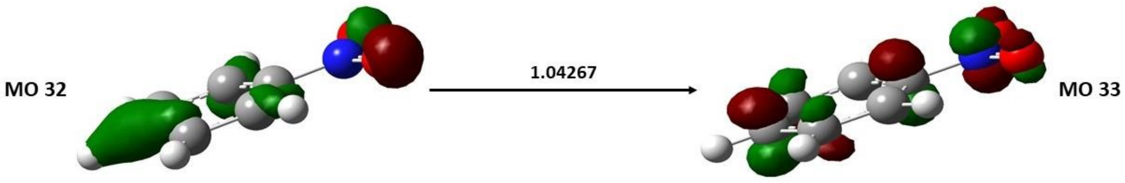

Figure S35. Natural transition orbitals with coefficient value for state **b** in Figure S33.

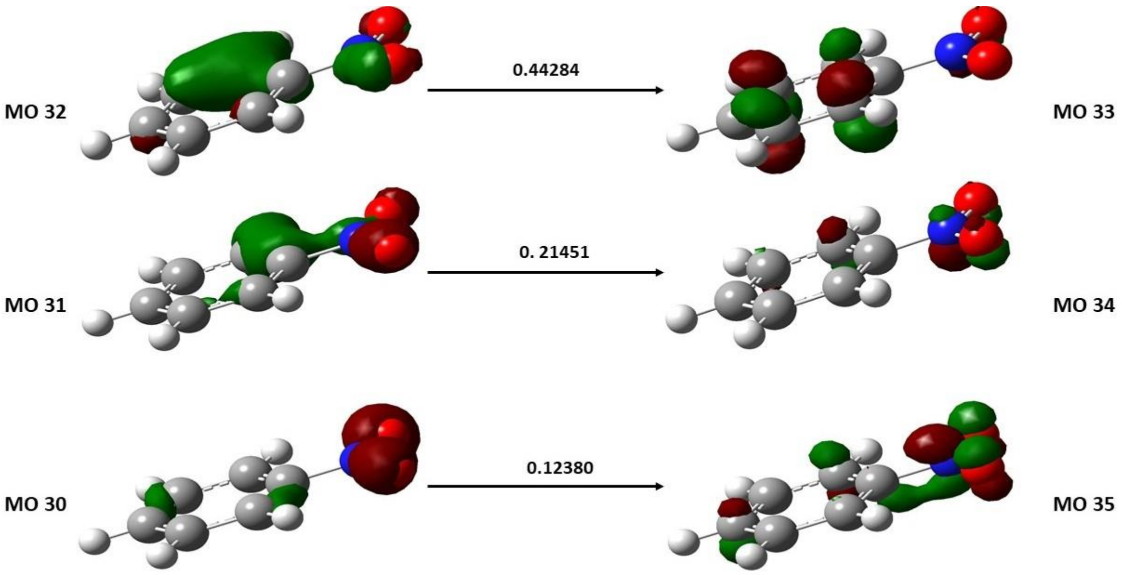

Figure S36. Natural transition orbitals with coefficient values for state **c** in Figure S33.

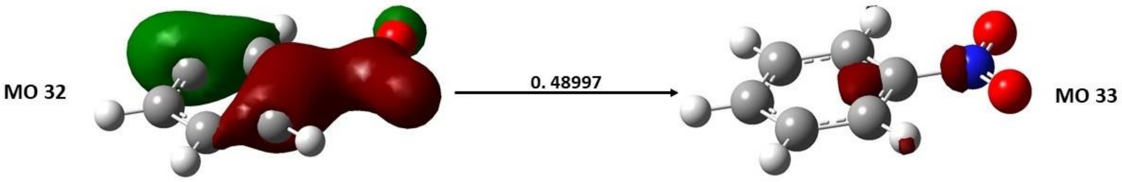

Figure S37. Natural transition orbitals with coefficient value for state **d** in Figure S33.

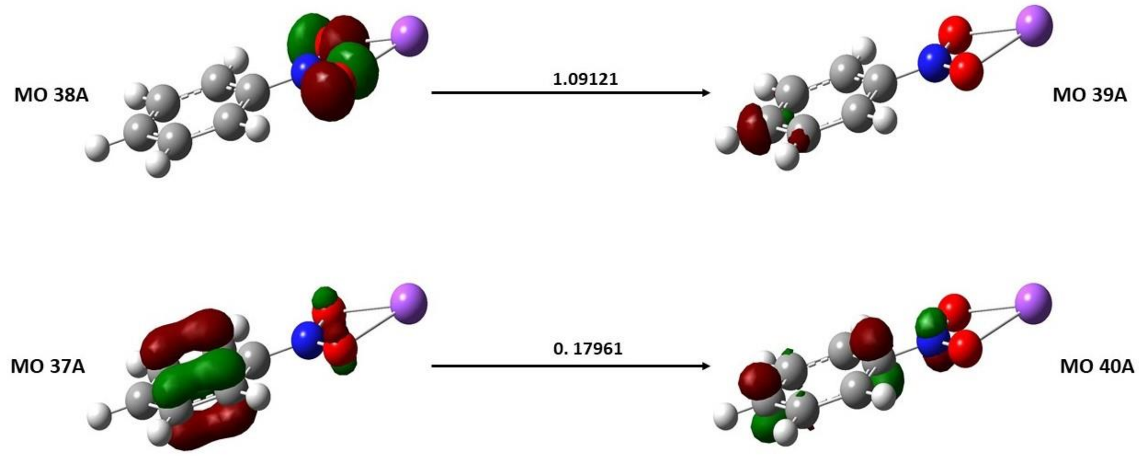

Figure S38. Natural transition orbitals (for alpha transition) with coefficients value for state **e** in Figure S33.

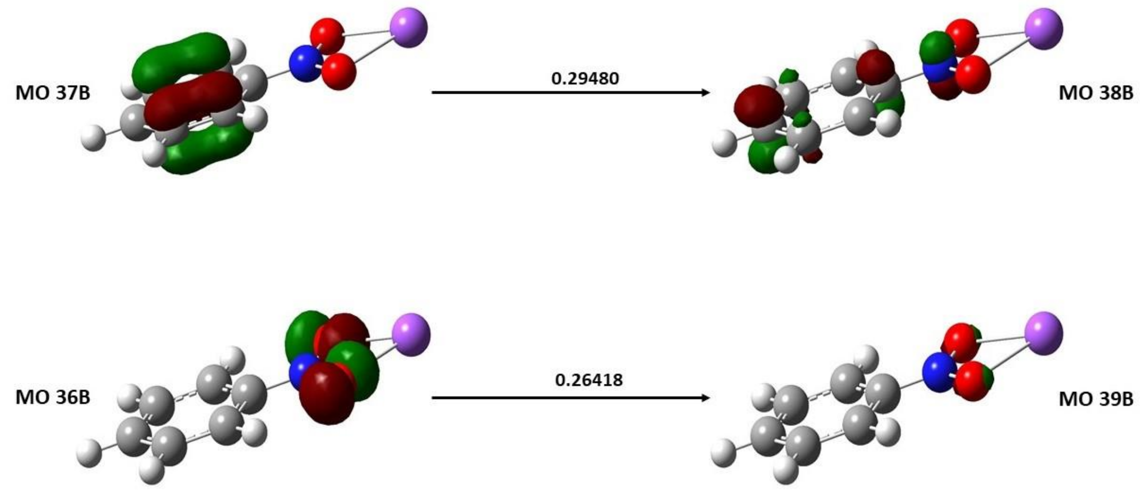

Figure S39. Natural transition orbitals (for beta transition) with coefficients value for state **e** in Figure S33.

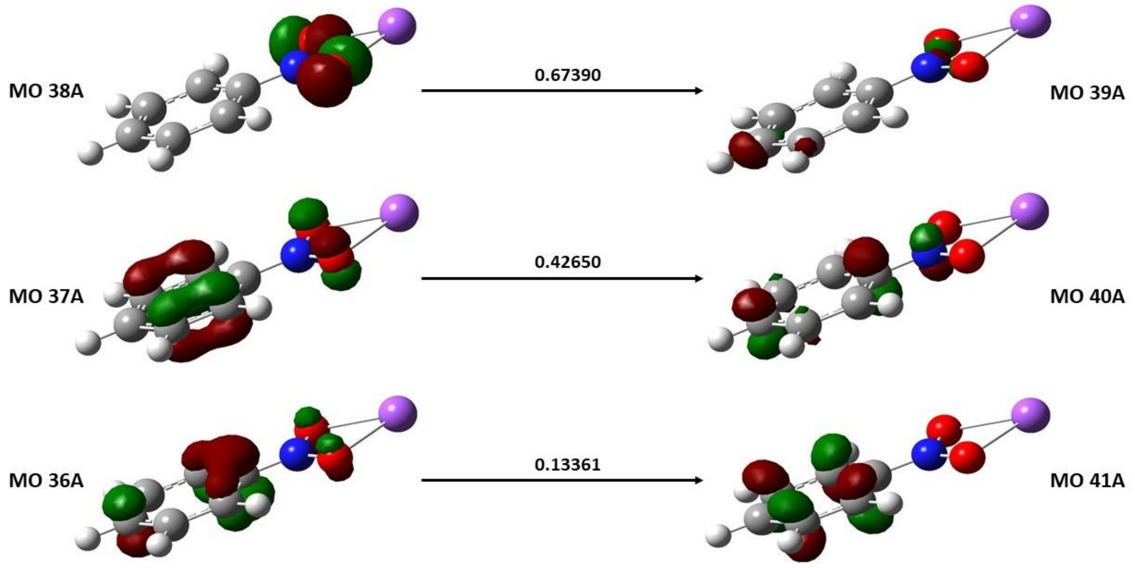

Figure S40. Natural transition orbitals (for alpha transition) with coefficients value for state **f** in Figure S33.

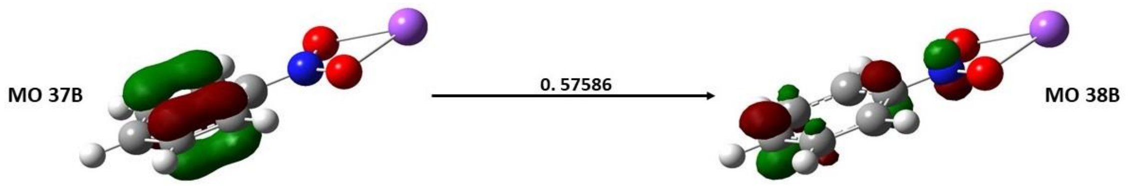

Figure S41. Natural transition orbitals (for beta transition) with coefficients value for state **f** in Figure S33.

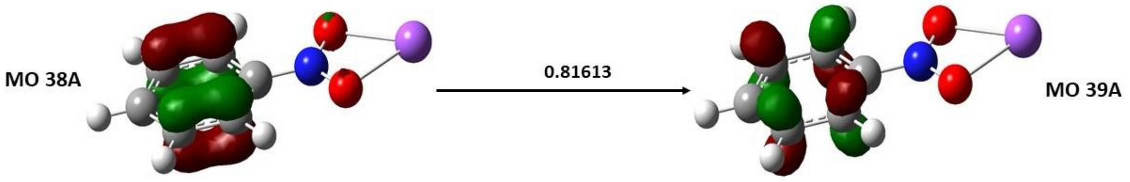

Figure S42. Natural transition orbitals (for alpha transition) with coefficients value for state **g** in Figure S33.

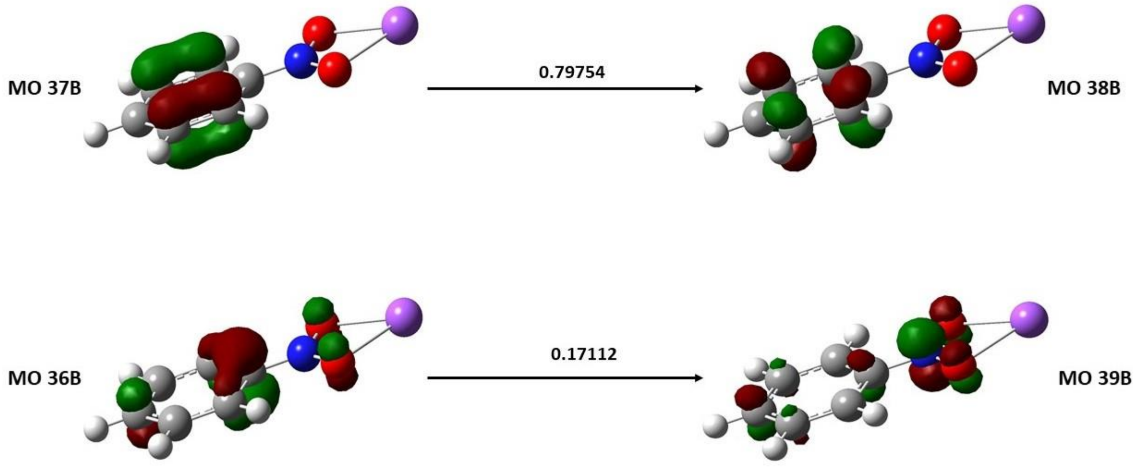

Figure S43. Natural transition orbitals (for beta transition) with coefficients value for state **g** in Figure S33.

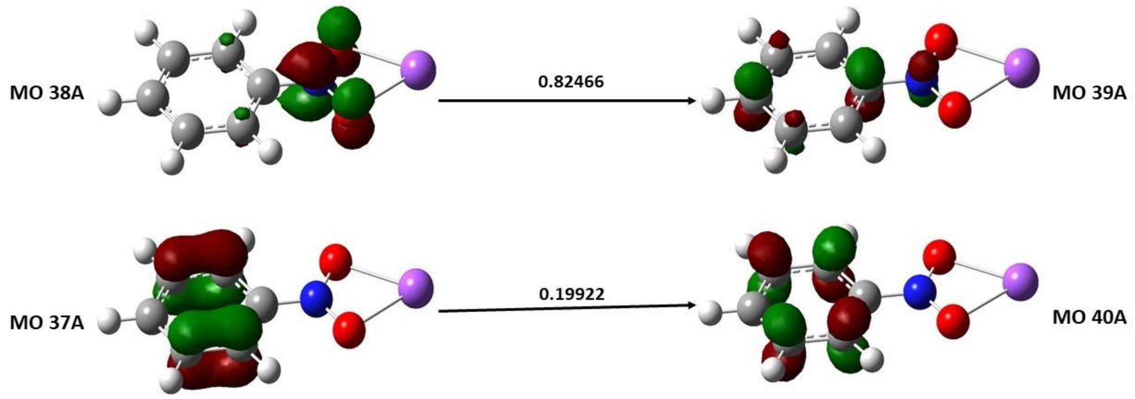

Figure S44. Natural transition orbitals (for alpha transition) with coefficients value for state **h** in Figure S33.

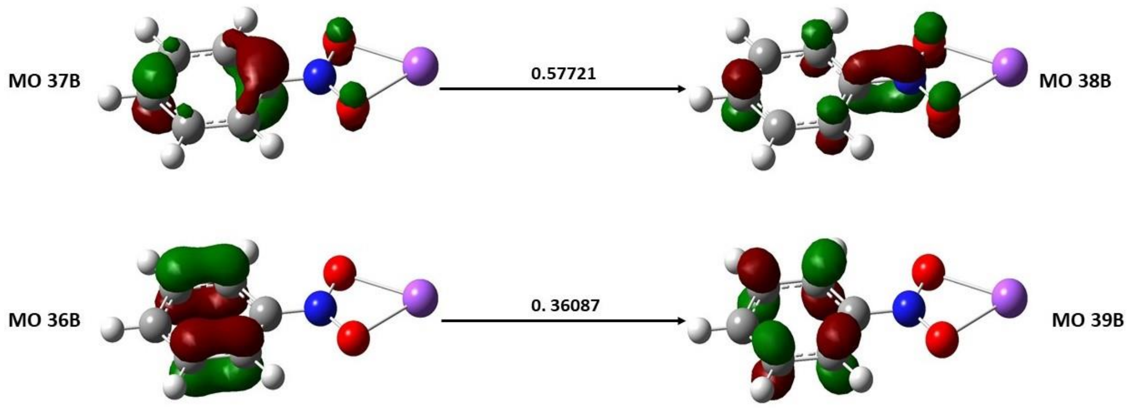

Figure S45. Natural transition orbitals (for beta transition) with coefficients value for state **h** in Figure S33.

Section S5: NMR spectra.

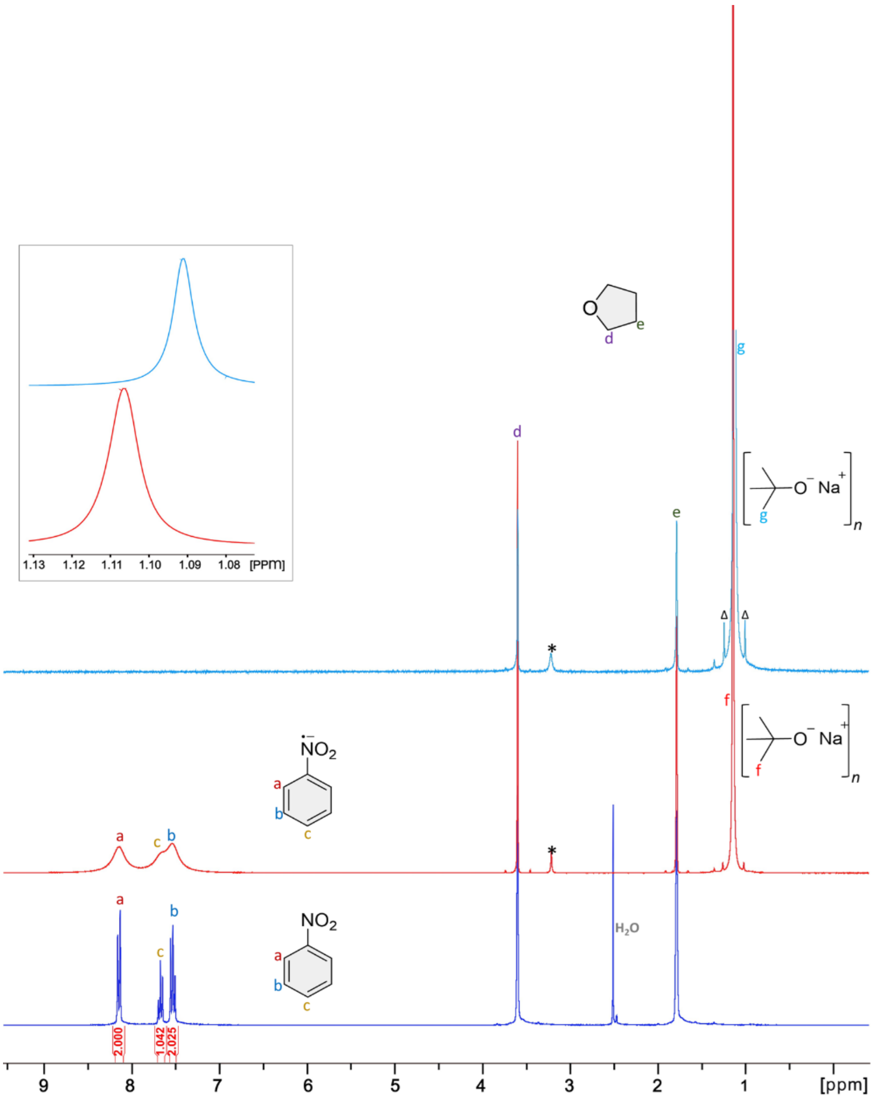

Figure S46. Comparison of a) NaO^{t}Bu, b) NaO^{t}Bu with **[1]** (equimolar ratio), and c) [**1**] ^{1}H NMR spectra in THF-d_8 solvent. * corresponds to methoxide impurity from NaO^{t}Bu. Triangles indicate satellite isotopic ^{13}C peaks.[14] The slight changes between g and f chemical shifts are assigned to differences in aggregation (n).

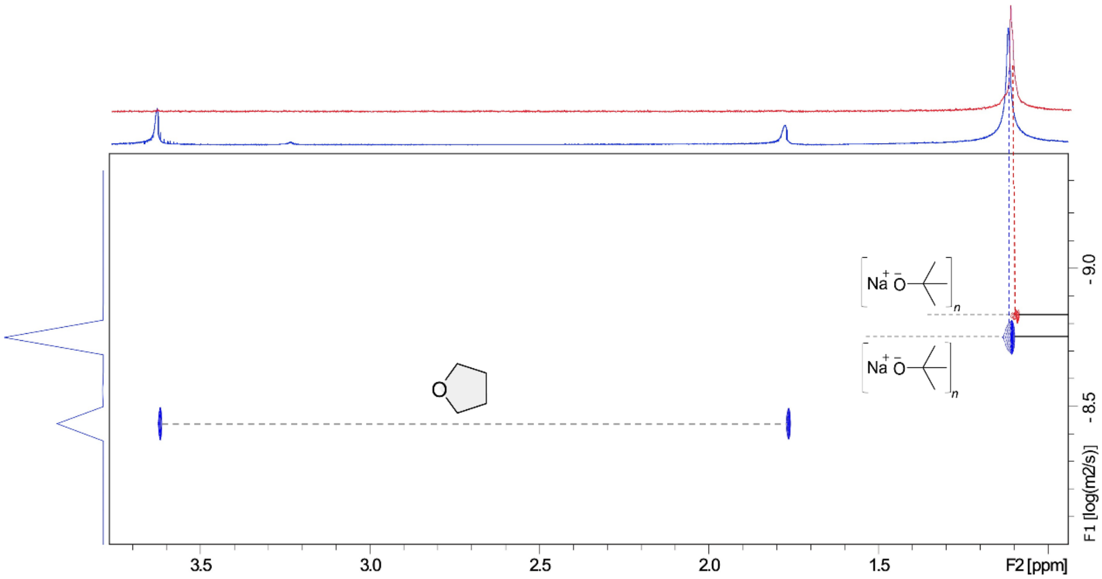

Figure S47. Comparison of NaO^{t}Bu (red traces) and NaO^{t}Bu with **[1]** (blue traces) in equimolar ratio ^{1}H DOSY spectra in THF-d_8 solvent. The slight changes between the NaO^{t}Bu chemical shifts are assigned to differences in aggregation (*n*).

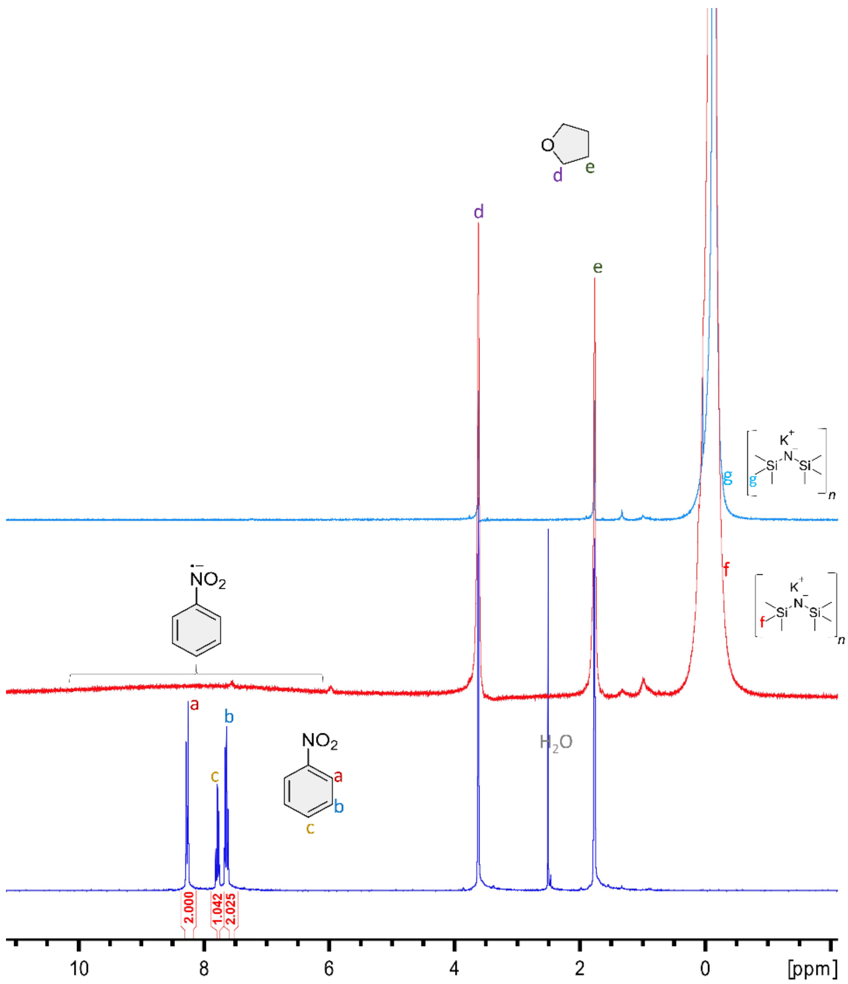

Figure S48. Comparison of a) KHMDS, b) KHMDS with **[1]** (equimolar ratio) ^{1}H NMR spectra in THF-d_8 solvent. The slight changes between g and f chemical shifts are assigned to differences in aggregation (n).

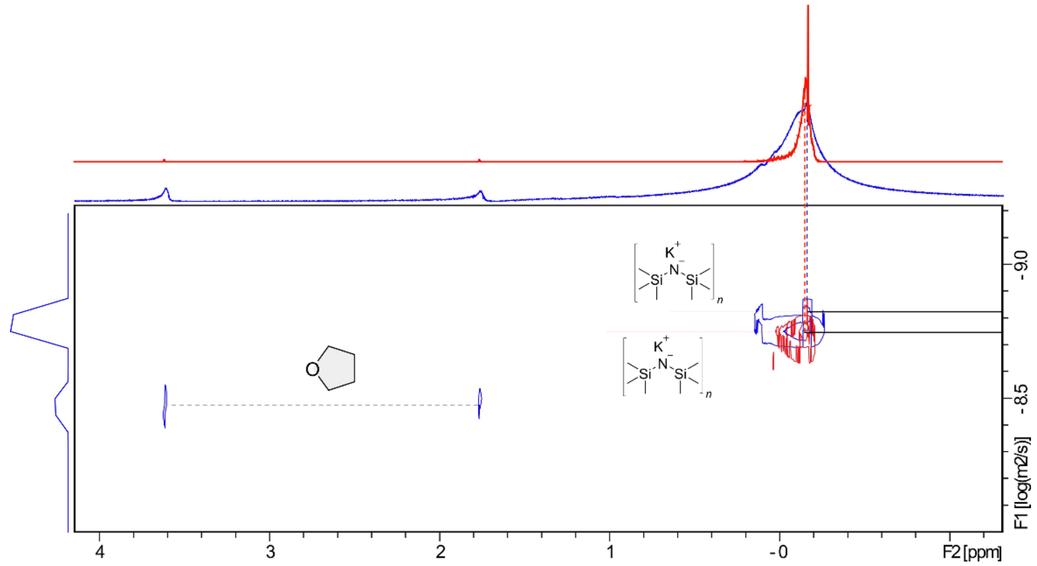

Figure S49. Comparison ^{1}H DOSY spectra KHMDS (red traces), KHMDS with **[1]** (blue traces) equimolar ratio in THF-d_8 solvent. The slight changes between the HMDS chemical shifts are assigned to differences in aggregation (n).

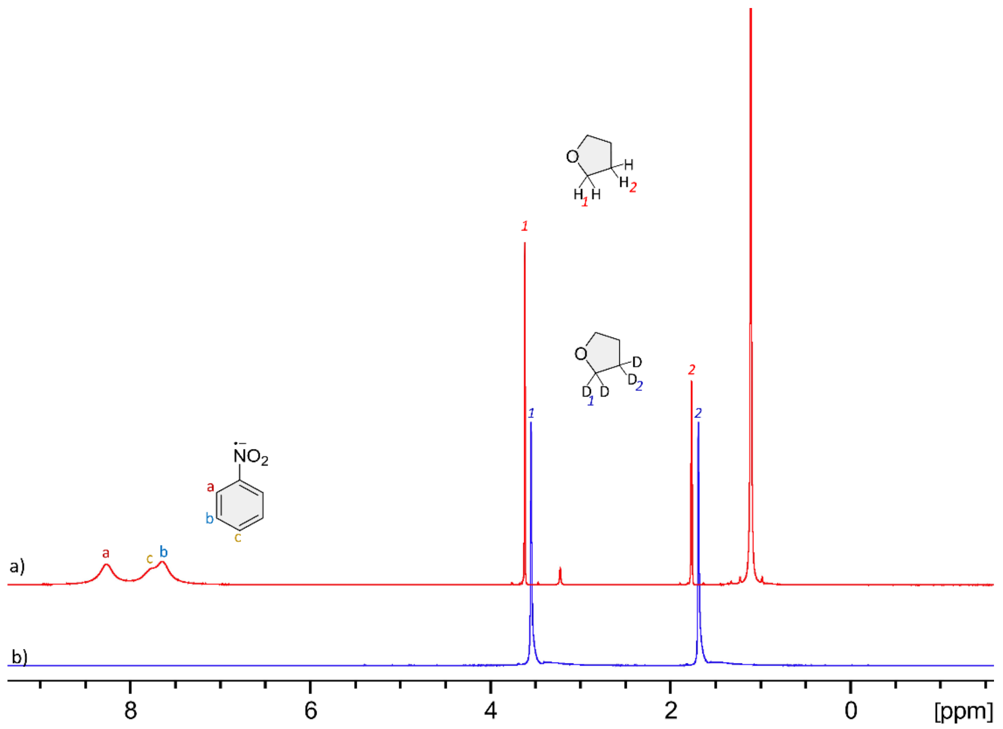

Figure S50. Comparison between a) ^{1}H NMR spectra of NaO^{t}Bu with **[1]** and b) ^{2}H NMR spectra of NaO^{t}Bu with **[1]** (equimolar ratio), in THF-d_8 solvent.

REFERENCES

1 D. H. Geske and A. H. Maki, *J Am Chem Soc*, 1960, **82**, 2671–2676.

2 P. H. Rieger and G. K. Fraenkel, *J Chem Phys*, 1963, **39**, 609–629.

3 R. L. Ward, *J Am Chem Soc*, 1961, **83**, 1296–1300.

4 C. Y. Ling and J. Gendell, *J Chem Phys*, 1967, **47**, 3475–3484.

5 F. J. Smentowski and C. D. Stevenson, *J Am Chem Soc*, 2002, **90**, 4661–4662.

6 J. M. Gross, J. D. Barnes and G. N. Pillans, *Journal of the Chemical Society A: Inorganic, Physical, Theoretical* , 1969, **109**, 109–112.

7 C. D. Stevenson, L. Echegoyen and L. R. Lizardi, *Journal of Physical Chemistry*, 1972, **76**, 1439–1442.

8 M. G. Davlieva, J. M. Lü, S. V. Lindeman and J. K. Kochi, *J Am Chem Soc*, 2004, **126**, 4557–4565.

9 R. D. Guthrie and D. E. Nutter, *J Am Chem Soc*, 1982, **104**, 7478–7482.

10 R. D. Guthrie, D. A. Hrovat, F. G. Prahl and J. Swan, *Journal of Organic Chemistry*, 1981, **46**, 498–501.

11 G. A. Russell, E. G. Janzen and E. T. Strom, *J Am Chem Soc*, 1962, **84**, 4155–4157.

12 G. M. Sheldrick, *urn:issn:2053-2733*, 2015, **71**, 3–8.

13 O. V. Dolomanov, L. J. Bourhis, R. J. Gildea, J. A. K. Howard and H. Puschmann, *J Appl Crystallogr*, 2009, **42**, 339–341.

14 P. Moutzouri, P. Kiraly, A. R. Phillips, S. R. Coombes, M. Nilsson and G. A. Morris, *Anal Chem*, 2017, **89**, 11898–11901.
